# Water-soluble trehalose glycolipids show superior Mincle binding and signaling but impaired phagocytosis and IL-1β production

**DOI:** 10.3389/fmolb.2022.1015210

**Published:** 2022-11-24

**Authors:** M. A. Thathsaranie P. Manthrirathna, Emma M. Dangerfield, Shigenari Ishizuka, Aodhamair Woods, Brenda S. Luong, Sho Yamasaki, Mattie S. M. Timmer, Bridget L. Stocker

**Affiliations:** ^1^ School of Chemical and Physical Sciences, Victoria University of Wellington, Wellington, New Zealand; ^2^ Centre for Biodiscovery, Victoria University of Wellington, Wellington, New Zealand; ^3^ Department of Molecular Immunology, Research Institute for Microbial Diseases, Osaka University, Osaka, Japan; ^4^ Laboratory of Molecular Immunology, Immunology Frontier Research Center, Osaka University, Osaka, Japan; ^5^ Division of Molecular Immunology, Medical Institute of Bioregulation, Kyushu University, Fukuoka, Japan; ^6^ Division of Molecular Immunology, Medical Mycology Research Center, Chiba University, Chiba, Japan

**Keywords:** Mincle, glycolipid, chemical immunology, synthesis, trehalose glycolipid, C-type lectin, adjuvant, inflammasome

## Abstract

The tremendous potential of trehalose glycolipids as vaccine adjuvants has incentivized the study of how the structures of these ligands relate to their Mincle-mediated agonist activities. Despite this, structure-activity work in the field has been largely empirical, and less is known about how Mincle-independent pathways might be affected by different trehalose glycolipids, and whether Mincle binding by itself can serve as a proxy for adjuvanticity. There is also much demand for more water-soluble Mincle ligands. To address this need, we prepared polyethylene glycol modified trehalose glycolipids (PEG-TGLs) with enhanced water solubility and strong murine Mincle (mMincle) binding and signaling. However, only modest cytokine and chemokine responses were observed upon the treatment of GM-CSF treated bone-marrow cells with the PEG-TGLs. Notability, no IL-1β was observed. Using RNA-Seq analysis and a representative PEG-TGL, we determined that the more water-soluble adducts were less able to activate phagocytic pathways, and hence, failed to induce IL-1β production. Taken together, our data suggests that in addition to strong Mincle binding, which is a pre-requisite for Mincle-mediated cellular responses, the physical presentation of trehalose glycolipids in colloidal form is required for inflammasome activation, and hence, a strong inflammatory immune response.

## 1 Introduction

**FIGURE 1:**
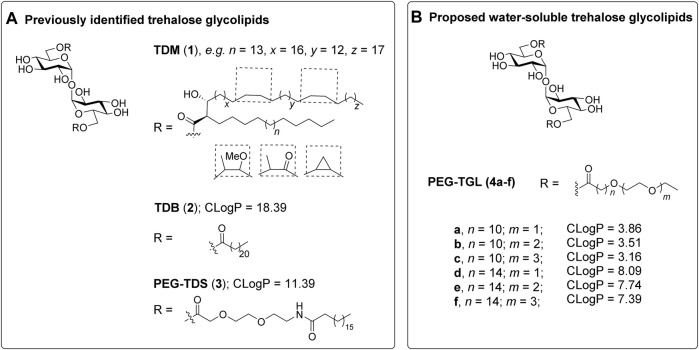
Trehalose glycolipids as Mincle-adjuvants. **(A)**. The previously reported trehalose glycolipids TDM (**1**) (Ishikawa et al., 2009) and TDB (**2**) (Schoenen et al., 2010) exhibit good Mincle-mediated adjuvanticity, however water-soluble adduct, PEG-TDS (**3**) (Huber et al., 2016) exhibits only modest adjuvanticity, which was attributed to poor mMincle binding. **(B)** We proposed that PEGylated trehalose glycolipids **4a-f** would retain good Mincle binding due to the positioning of the PEG groups at the terminus of the lipid. The calculated LogP values provide an indication of water solubility.

In recent years there has been much interest in the development of synthetic Macrophage Inducible C-type lectin (Mincle, Clec4e, or Clecsf9) agonists (Braganza et al., 2018; Williams, 2017; O’Hagan et al., 2020; Cramer, 2021). In particular, trehalose glycolipids have shown much promise as Mincle-mediated adjuvants (Foster et al., 2018; Ryter et al., 2020; Foster et al., 2020; Rasheed et al., 2020; Lynch et al., 2021; Ryter et al., 2021; Dangerfield et al., 2022). Trehalose dimycolate (TDM, **1**, Figure 1A), a heterogenous pathogen associated molecular pattern (PAMP) isolated from the cell wall of *Mycobacterium tuberculosis*, and trehalose 6,6′-dibehenate (TDB, **2**), a linear C22-synthetic derivative thereof, were the first non-proteinaceous Mincle ligands identified (Ishikawa et al., 2009; Schoenen et al., 2010). Following binding of TDM or TDB to Mincle, induction of the FcRγ-Syk-Card9-Bcl10-Malt1 signaling axis occurs, which leads to the NFκB-mediated expression of cytokines, chemokines, and small molecule mediators. (Ishikawa et al., 2009; Werninghaus et al., 2009; Schoenen et al., 2010; Kodar et al., 2017). Ultimately, these cellular mediators influence the adaptive immune response and T-helper cell differentiation. TDB has been formulated into a variety of dimethyldioctadecylammonium bromide (DDA)-containing liposomes, with or without other pathogen-associated molecular patterns (Pedersen et al., 2018). These liposomes have found wide application in several different vaccination models (Pedersen et al., 2018), with the DDA/TDB adjuvant system, CAF01, being used in clinical trials for HIV and TB vaccination (Kaufmann et al., 2010; Fomsgaard et al., 2011).

In addition to introducing new vaccine adjuvants, determining how changes to the structure of the trehalose glycolipid influences the ensuing immune response generates valuable mechanistic knowledge about those features of the trehalose glycolipid that are required for optimal agonist activity (Bird et al., 2018; Foster et al., 2018; Khan et al., 2018; Ryter et al., 2020; Dangerfield et al., 2022). As part of such studies, there has been interest in the development of TDB-analogues with improved water solubility to facilitate the formulation and delivery of this class of ligands (Jacobsen et al., 2015; Huber et al., 2016; O’Hagan et al., 2020). Lang and co-workers synthesized 6,6′-bis [8-(stearoylamido)-3,6-dioxaoctanoyl]-trehalose (PEG-TDS **3**), which has a hydrophilic polyethylene glycol (PEG) group between the sugar moiety and the acyl chain (Huber et al., 2016). Unfortunately, PEG-TDS **3**) exhibited modest adjuvanticity, both *in vitro* using bone marrow derived macrophage (BMDM) and bone marrow derived dendritic cell (BMDC) assays, and *in vivo* immunization assays using PEG-TDS/DDA liposomes with an antigen from *Chlamydia trachomatis*. The author’s attributed the limited immunostimulatory ability of PEG-TDS **3**) to a lower binding affinity to Mincle (Huber et al., 2016).

**FIGURE 2:**
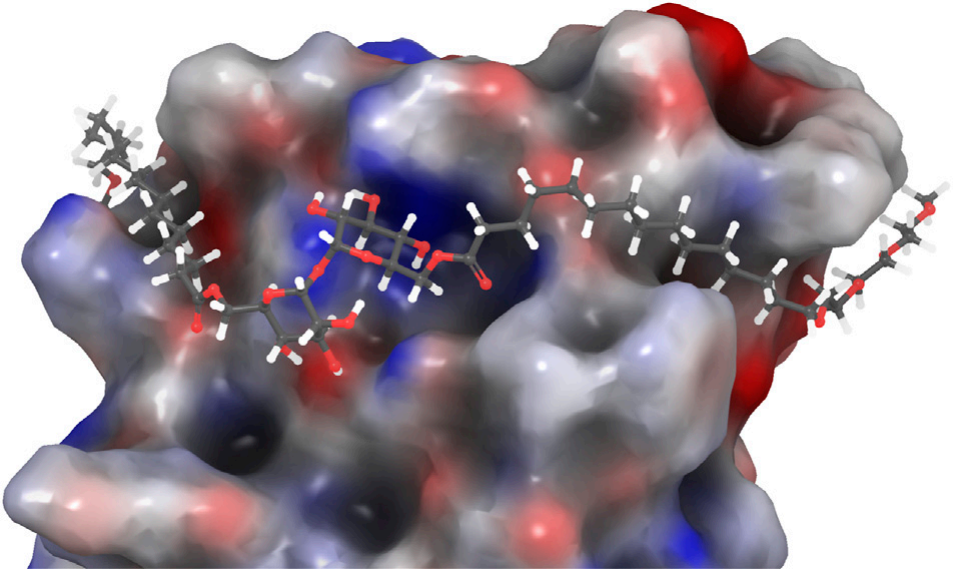
PEG-TGL **4f** (*m* = 3, *n* = 14) docked in the binding site of hMincle (3WH2). The hydrophobic portion of the lipid binds to the hydrophobic groove of Mincle, while the hydrophilic PEG groups are found outside this binding groove and can increase the water solubility of the ligand (Maestro, 2021).

Despite these observations, we were interested in determining whether a more water-soluble trehalose glycolipid with improved Mincle-binding, and hence, agonist-activity, could be developed. We reasoned that the placement of the water-soluble PEG spacer towards the end of an acyl chain would provide a portion of hydrophobic lipid that would be able to interact with the hydrophobic groove of Mincle (Feinberg et al., 2013; Furukawa et al., 2013; Feinberg et al., 2016), while the terminal PEG would improve the water solubility of the ligand. Molecular docking of PEG-modified TGLs showed that aliphatic chain lengths of around 12 carbons would be ideal to span Mincle’s lipophilic groove (Figure 2) (Maestro, 2021). This in turn allows for the incorporation of ethyleneglycol units at the lipid termini to provide for a more hydrophilic ligand. Thus, PEGylated trehalose glycolipids (PEG-TGLs, **4a-f**) with differing lipophilic acyl chain lengths (*n* = 10 and 14) and number of PEG groups (*m* = 1, 2 or 3) were designed and the CLogP values calculated to provide an indication of lipophilicity (Leo et al., 1971), and hence, water solubility (Figure 1B).

However, during our studies, we unexpectedly observed that Mincle binding affinity does not correlate to the immunomodulatory activity of the ligands, though does correlate to Mincle signaling. Moreover, our mechanistic studies suggest that this lack of functional immune response results from the inability of the water-soluble ligands to activate the inflammasome. Due to the importance of Mincle ligands as a burgeoning class of vaccine adjuvant (Williams, 2017; Braganza et al., 2018), our findings provide much needed guidance in the field of Mincle agonist design and shed light on the mechanisms involved in immune cell activation through Mincle- and non-Mincle-mediated pathways.

## 2 Materials and methods

### 2.1 Ethics

C57BL/6 wild-type and Mincle−/− mice were bred and housed in a conventional animal facility at the Malaghan Institute of Medical Research, Wellington, New Zealand. The experimental procedures used in this study were approved by the Victoria University Animal Ethics Committee (ethics approval number 26793) and by the Animal Care and Use Committee of the Research Institute for Microbial Diseases, Osaka University (Biken-AP-R03-17-0).

### 2.2 Chemical synthesis and analysis

PEG-TGLs **4a-f** were synthesized from commercially available starting materials. TDB was prepared according to literature procedures (Khan et al., 2011). Full experimental procedures and compound characterization data are provided in the supporting information. Critical micelle concentrations were determined by dynamic light scattering (DSL) with a ZetasizerNano ZS (Malvern Instruments Ltd., Malvern, Worcestershire, United Kingdom) at 20°C. Data was analyzed using Malvern Dispersion Technology Software. All synthesized glycolipids were confirmed to be endotoxin free at a sensitivity level of ≤ 0.25 EU/mL by using the ToxinSensor^™^ Gel Clot Endotoxin Assay Kit (Gen Script) prior to biological evaluation. CLogP values were calculated using ChemDrawStd v 11.0.

### 2.3 Preparation of ligand-coated plates

Synthesized PEGylated glycolipids (**4a-f**) and TDB (**2a**) were dissolved in CHCl_3_:MeOH (2:1) to make stock solutions of 1 mM. The stock solutions were diluted to the appropriate concentration in isopropanol then added to 96-well plates (20 μL/well), and the solvent evaporated in a sterile hood.

### 2.4 NFAT-GFP Mincle reporter assays

2B4 T cells expressing NFAT-GFP along with mouse Mincle + FcRγ, human Mincle + FcRγ, or FcRγ only were maintained as previously described (Yamasaki et al., 2008). The 2B4 T cells transfected with NFAT-GFP were cultured in complete Roswell Park Memorial Institute medium [RPMI-1640 supplemented with 2 mM Glutamax (Gibco), 10% (v/v) fetal bovine serum (Gibco) and 1% (v/v) penicillin-streptomycin (Gibco)]. Cells (4 × 10^5^ cells/mL, 100 μL/well) were then incubated with ligands coated on plates (0.1 or 1 nmol/well) for 18 h. The reporter cells were then harvested, stained with 4′,6-diamidino-2-phenylindole (DAPI), and NFAT-GFP expression monitored by flow cytometry (FACS Canto II). Expression of GFP by reporter cells were given as a percentage of total live, single cells.

### 2.5 GM-CSF treated bone-marrow cell assay with ligand-coated plates

Bone-marrow cells were collected from femurs and tibias of wild type (C57BL/6) or C57BL/6 Mincle^−/−^ mice and the cells cultured as previously reported (50 ng/ml of GM-CSF) (Stocker et al., 2019). On day 8, all media together with non-adherent cells were removed by aspirating. Cells were harvested using Acutase (0.5 ml/well, 10–15 min) and resuspended at 1 × 10^6^ cells/mL in cRPMI and were added (200 μL/well) to 96 well plates coated with TDB or trehalose glycolipids **4a-f** (0.1 or 1 nmol/well). A positive control with 100 ng/ml LPS and a negative control with isopropanol were used. Stimulated cells were incubated for 24 h at 37°C before supernatants were collected and analyzed for cytokine or chemokine production. In an additional experimental set-up, bone-marrow cells from wild type (C57BL/6) mice were harvested and treated in the same manner as noted above except that 20 ng/ml of GM-CSF was added to the cell culture.

### 2.6 GM-CSF treated bone-marrow cell assay with solubilized ligands

Bone-marrow cells were collected from femurs and tibias of wild type (C57BL/6) or Mincle^−/−^ mice and the cells cultured as previously reported (50 ng/ml of GM-CSF) (Stocker et al., 2019). On day 8, all media together with non-adherent cells were removed and 0.25 ml fresh media was added to each well (48 well plate). Stock solutions of TGLs **4a-f** and TDB (1 mM, with 2% DMSO in sterile water) were prepared and kept sterile. From stock solutions, 1 or 10 μL were added to each well, to give a final ligand concentration of 4 or 40 μM in a total well volume of 250 μL. A positive control with 100 ng/ml LPS and a negative control with untreated cells were used. Stimulated cells were incubated for the indicated time (24 or 48 h) before supernatants were analyzed for cytokine production.

### 2.7 Cytokine and chemokine analysis

Levels of IL-1β (R&D systems), IL-6 (BD Biosciences), IL-10 (R&D systems), and MIP-2 (R&D systems) were determined using sandwich ELISA according to the manufacturer’s instructions.

### 2.8 mMincle binding assay

Trehalose glycolipids (**4c** and **4f**) were coated on plates and incubated with mMincle-Ig or Ig alone [at a concentration of 3 μg/ml in binding buffer (1% BSA/TSM, BSA: bovine serum albumin; TSM buffer (pH7.0): 20 mM Tris-HCl, 1 mM CaCl_2_, 150 mM NaCl and 2 mM MgCl_2_)]. The preparation of Mincle fusion proteins was undertaken as described previously (Yamasaki et al., 2008). TDM (Sigma-Aldrich) and TDB were used as positive controls. Ligand bound protein mMincle-Ig was then detected using HRP-labelled anti-human Ig antibody *via* ELISA by optical density (OD) measurement at 450 nm [Multiskan JX and Ascent Software version 2.6 (Thermo Fisher Scientific)] and analyzed using Microsoft Excel (Microsoft).

### 2.9 mMincle competition assay

Glycolipids **4c** or **4f** (0, 0.01, 0.1 or 1 nmol/well) were co-plated with or without TDM or TDB (0.01 nmol/well) and incubated with NFAT-GFP reporter cells. GFP expression by the reporter cells, as determined using a flow cytometer (Attune NxT Flow Cytometer, Thermo Fisher), was used as a readout to measure the ability of the analogues to compete with TDM or TDB for recognition by Mincle.

### 2.10 RNA-Sequence protocol

Bone-marrow cells were collected from femurs and tibias of wild type (C57BL/6) mice and cells were cultured in RPMI-1640 supplemented with 10% (v/v) fetal bovine serum (NICHIREI), 2-mercaptoethanol (DS Pharma), penicillin G (Sigma-Aldrich), streptomycin (MP Biomedicals), and 20 ng/ml of mouse GM-CSF (BioLegend). On day 3, 10 ml of GM-CSF-containing media was added, and on day 9, cells were harvested and used for stimulation. 1 × 10^6^ cells were stimulated with 0.6 nmol/well of indicated stimulants coated on a 24-well plate for 4 h. Cells were lysed with QIAsol (QIAGEN) to extract total RNA from the cells according to the manufacturer’s instructions. Library preparation was performed by TruSeq standard mRNA sample prep kit (Illumina) and whole transcriptome sequencing was applied to the RNA samples by using Illumina HiSeq 2,500 platform in 75-base single-end mode. The Illumina Casava ver.1.8.2 software was used for base calling. Sequenced reads were mapped to the mouse reference genome sequences (mm10) using TopHat ver.2.0.13 in combination with Bowtie2 ver.2.2.3 and SAMtools ver.0.1.19. The number of fragments per kilobase of exon per million mapped fragments was calculated using Cufflinks ver.2.2.1. Differential expression and pathway analysis of the RNA-Seq data was performed using iDEP (Ge et al., 2018), and KEGG pathway enrichment analysis and visualizations were generated using Pathview Web (Luo et al., 2017).

## 3 Results

### 3.1 Synthesis of PEGylated trehalose glycolipids

**FIGURE 3:**
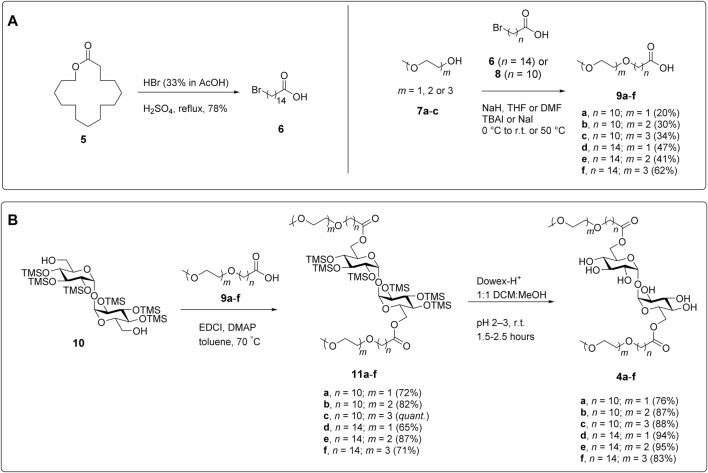
Synthesis of PEG-TGLs. **(A)** Preparation of the carboxylic acids required to install the PEGylated side chain. **(B)** Conjugation of the suitably functionalized trehalose scaffold (Khan et al., 2011) to the carboxylic acid side chains and deprotection to give the target PEG-TGLs (**4a-f**).

To synthesize the polyethyleneglycol modified trehalose glycolipids (PEG-TGLs) **4a-f**, we first prepared a series of PEGylated carboxylic acids for subsequent coupling to trehalose. While 11-bromoundecanoic acid (**8**) is commercially available, 15-bromopentadecanoic acid (**6**) was synthesized from pentadecanolide (**5**) in a single step (78% yield), by treatment with HBr and H_2_SO_4_ (Davey and Hayman, 1998; Carter et al., 2012) (Figure 3A). Both bromides were then used in Williamson ether syntheses with mono-, di- and triethyleneglycol monomethyl ethers **7a-c** to afford carboxylic acids **9a-f** in 20–63% yield. For each ether, the formation of the linkage between bromo-carboxylic acids and ethylene glycol was confirmed by the observation of an HMBC between the terminal methylene protons of the alkyl chain (ca. δ 3.43 ppm) and the first carbon of the glycol moiety (ca. δ 70.1 ppm). During the ether syntheses, we observed the undesired β-elimination of the bromine atom in the starting carboxylic acid to give a terminal alkene in ca. 10% yield along with other unidentified by-products. Unfortunately, changes to the order and equivalents of reagents added, reaction temperatures, and solvent did not improve the yields. That said, it should be noted that similar yields have been reported for related compounds (Baker et al., 2013), illustrating the difficulties of undertaking Williamson ether syntheses using long lipophilic chains.

With the PEGylated side chains in hand, α,α′-D-trehalose was per-silylated under the agency of *N,O*-bis(trimethylsilyl)acetamide (BSA) and catalytic tetra-*N*-butylammonium fluoride (TBAF), followed by treatment with K_2_CO_3_ to afford TMS-protected trehalose **10** (Figure 3B) according to previously published procedures (Johnson, 1992; Khan et al., 2011). An EDCI-mediated esterification reaction between diol **10** and carboxylic acids **9a-f** gave diesters **11a-f**. Subsequent removal of the TMS groups under the agency of Dowex-H^+^ resin afforded the target PEG-TGLs **4a-f** in good overall yield. When performing the final deprotection step it is important to ensure that the compounds are properly neutralized before storage otherwise they are prone to hydrolysis. We speculate that this might be due to the ability of the PEG-TGLs to co-ordinate to cations (e.g., H^+^, Na^+^) in much the same way that crown-ethers coordinate cations to augment base- or acid-catalyzed hydrolysis reactions.

### 3.2 PEGylated trehalose glycolipids exhibit robust mincle signaling but induce moderate chemokine and cytokine production by GM-CSF treated bone marrow cells

**FIGURE 4:**
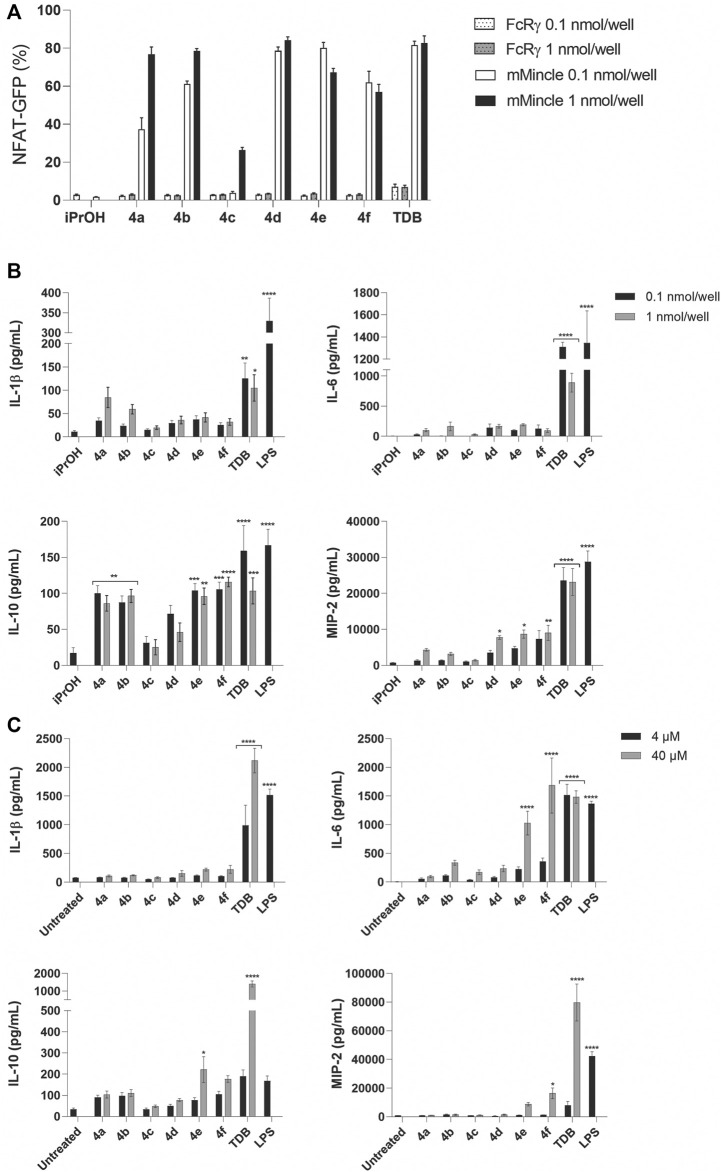
PEGylated TGLs (**4a-f**) led to strong mMincle signaling but modest cytokine production. **(A)** Reporter cells expressing mMincle + FcRγ or FcRγ only were stimulated with ligands coated on plates (0.1 and 1 nmol/well). NFAT-GFP expression by the harvested cells after 18 h was measured using flow cytometry. Data represents the mean of two independent experiments performed in duplicate (mean ± SEM). **(B)** WT C57 GM-CSF treated BMCs were stimulated with TDB or **4a-f** (0.1 or 1 nmol/well) coated on plates, or LPS (100 ng/ml), and IL-1β, IL-6, IL-10 and MIP-2 were measured by ELISA from the supernatants collected after 24 h. Data represents the mean of three independent experiments performed in triplicate (mean ± SEM). Statistical significance was calculated in comparison to iPrOH control using two-way ANOVA (Dunnett’s multiple comparison test), *p ≤ 0.05; **p ≤ 0.01; ***p ≤ 0.001; ****p ≤ 0.0001. **(C)** GM-CSF treated BMCs were stimulated with TDB or trehalose glycolipids **4a-f** (4 µM or 40 μM; solubilized in 2% DMSO in H_2_O), or LPS (100 ng/ml). IL-1β, IL-6, IL-10 and MIP-2 were measured by ELISA from the supernatants collected after 48 h. Data are representative of three independent experiments performed in triplicate (mean ± SEM). Statistical significance was calculated in comparison to untreated control using two-way ANOVA (Dunnett’s multiple comparison test), *p ≤ 0.05; **p ≤ 0.01; ***p ≤ 0.001; ****p ≤ 0.0001.

The PEGylated glycolipids **4a–f** were tested for their ability to signal through Mincle using a NFAT-GFP reporter cell assay employing cells expressing mouse Mincle + FcRγ, or FcRγ only, at two different concentrations (0.1 and 1 nmol/well) of glycolipid. Analogues **4a** (CLogP = 3.86) and **4b** (CLogP = 3.51) with a shorter hydrophobic acyl length (*n* = 10) and with 1 or 2 glycol groups (m = 1 or 2, respectively), led to Mincle-mediated signaling in a dose dependent manner (Figure 4A). However, at the lower ligand concentration, the ability of **4a** to signal through Mincle was slightly reduced compared to analogues **4d-f**, which have longer lipid length (*n* = 14) and therefore greater CLogP values (i.e., CLogP = 8.09–7.39). The most water-soluble adduct, **4c** (*n* = 10, *m* = 3, CLogP = 3.16) exhibited only a modest ability to signal through mMincle, and only at the highest ligand concentration tested (1 nmol/well). For the adducts containing a longer lipophilic portion (i.e., **4d-f**, *n* = 14), there was little difference in the percentage of GFP-producing reporter cells at the two different ligand concentrations.

The PEGylated glycolipids **4a-f** were then evaluated for their ability to generate a functional immune response, as determined by the production of the cytokines IL-1β, IL-6, and IL-10, and chemokine MIP-2, by GM-CSF-treated bone marrow cells (BMCs) in response to the ligands (Figure 4B). The GM-CSF promoted differentiation of cultured BMCs produces a heterogenous mixture of monocytes/macrophages, granulocytes, and dendritic cells (DCs) (Na et al., 2016; Sun et al., 2018). Using a high concentration of GM-CSF (50 ng/ml) favors a high proportion of monocytes/macrophages (Sun et al., 2018). Plates were coated with the ligands at concentrations of 0.1 and 1 nmol/well and cytokine and chemokine production measured at 24 h. As anticipated, TDB led to significant levels of IL-1β, IL-6, IL-10, and MIP-2, and the most water-soluble adduct **4c**, which led to poor Mincle signaling, did not elicit a functional immune response. In contrast, the PEGylated glycolipids **4e** and **4f** led to statistically significant levels of IL-10 and MIP-2 but did not lead to the production of IL-6 or IL-1β, while **4a** and **4b** led to only the production of IL-10. All responses were Mincle-dependent, as demonstrated using Mincle−/− GM-CSF-treated BMCs (Supplementary Figure S1). In addition, we measured the response of BMCs treated with 20 ng/ml of GM-CSF, followed by stimulation with the glycolipids. This concentration of GM-CSF should also lead to a high proportion of monocytes/macrophages (Sun et al., 2018). With lower levels of GM-CSF added to the culture media, TDB, but not the other PEGylated adducts, led to significant levels of IL-1β, IL-10 and MIP-2 (Supplementary Figure S2). The small differences that were observed between the assay setups may be due to minor differences in the heterogeneity of the cell culture, or it could be due to the effect of GM-CSF itself (Bergamini et al., 2000).

To determine whether the activity of the PEGylated adducts could be improved by altering their physical presentation (Stocker et al., 2019), we added **4a-f** to PBS (Khan et al., 2011; Stocker et al., 2014; Stocker et al., 2019) rather than using the ligand-coated format and measured the production of IL-1β, IL-6, IL-10 and MIP-2 by BMCs at 24 h (Supplementary Figure S3) and 48 h (Figure 4C). As anticipated, greater cytokine production was observed at 48 h (Khan et al., 2011), with levels of IL-6 being significantly enhanced by **4f** and being comparable to those induced by TDB. However, no IL-1β was observed in response to any of the PEGylated glycolipids. To confirm the solubility of compound **4f**, we analyzed both **4f** and TDB using dynamic light scattering (DLS), and found that while TDB formed micelles at concentrations above 1.0 μM, no aggregates were observed for **4f**.

### 3.3 PEGylated TGL 4f binds more strongly to mMincle than TDB but is not a competitive inhibitor

**FIGURE 5:**
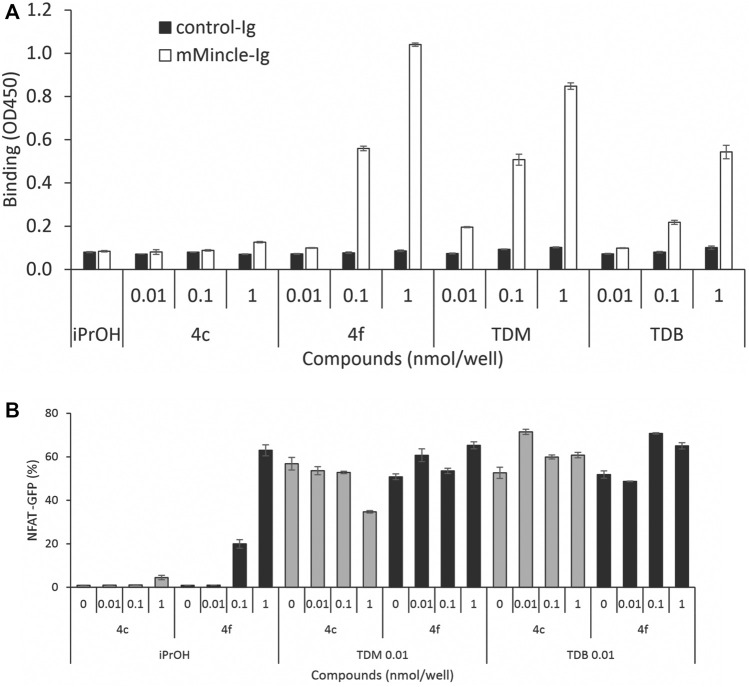
Water-soluble analogue **4f** is a better ligand for mMincle compared to TDB and TDM but does not competitively inhibit the binding of TDB or TDM to mMincle. **(A)** Ligand coated plates (0.01, 0.1 or 1 nmol/well) were incubated with mMincle-Ig or Ig alone (3 μg/ml) for 14 h, and ligand bound protein determined using ELISA. Data obtained in a representative experiment performed in triplicate. **(B)** 2B4-NFAT-GFP reporter cells expressing mMincle were stimulated with **4c** or **4f** coated on plates (0, 0.01, 0.1 or 1 nmol/well) with TDM or TDB (0.01 nmol/well). GFP expression by the reporter cells was measured using flow cytometry after 18 h. Data obtained in a representative experiment performed in triplicate.

To investigate whether cytokine production was related to Mincle binding affinity, we determined the mMincle binding affinity of two representative compounds with the same number of PEG groups (*m* = 3) but with acyl chain portions of *n* = 10 (**4c**) or *n* = 14 (**4f**). The compounds were assessed for their binding affinity to mMincle-Ig at 0.01, 0.1, and 1.0 nmol/well, with ligand bound mMincle-Ig being detected through HRP-labelled anti-human Ig antibody using ELISA. Analogue **4c** with the shorter hydrophobic portion (*n* = 10, CLogP = 3.16) showed no mMincle-Ig binding affinity, while **4f**, with the longer hydrophobic portion (*n* = 14, CLogP = 7.39), bound mMincle with a higher affinity than TDB (**2**, CLogP = 18.39) at concentrations of 0.1 and 1 nmol/well (Figure 5A). Moreover, the mMincle binding affinity of **4f** was slightly greater than that of TDM (**1**) at 1 mol/well, and much greater than that of TDB (**2**). Molecular docking of TDB and PEG-TGLs **4c** and **4f** into the hMincle binding site supported this observation with PEG-TGL **4f** possessing the ideal number of CH_2_ units to span the Mincle hydrophobic groove (*cf.* Figure 2). The strong mMincle binding affinity of **4f** prompted us to explore whether **4f** might be a competitive inhibitor of TDB, and hence perhaps able to reduce the strong inflammatory response induced by TDM. However, **4f** was not able to inhibit reporter cell activation by TDB or TDM at any of the concentrations tested (Figure 5B).

### 3.4 TDB and **4f** activate similar immune cell signalling pathways although differences include pathways related to phagocytosis

**FIGURE 6:**
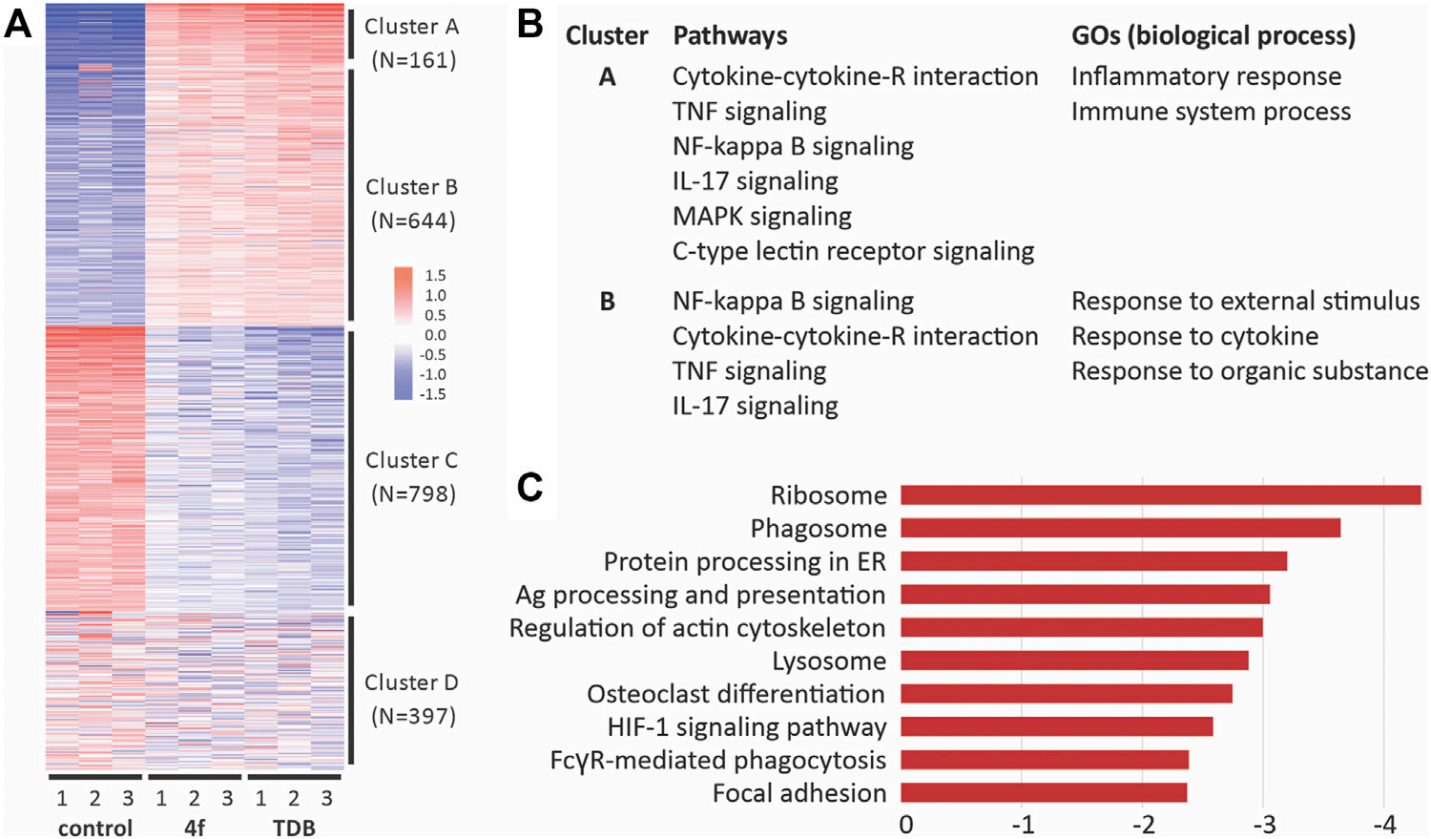
RNA-Seq analysis of gene pathway following the activation of GM-CSF treated BMCs with **4f** and TDB reveals similarities in immune cell signaling but also differences in phagocytic pathways. **(A)**. Hierarchical clustering of differentially expressed genes. **(B)**. Functional enrichment of differentially expressed genes (DEGs) in response to **4f** and TDB. **(C)**. KEGG pathway enrichment analysis showing the top 10 down-regulated pathways between **4f** vs TDB control samples.

We used RNA sequencing to analyze the transcriptome control of immune cell activation pathways upon the stimulation of GM-CSF treated BMCs with TDM or **4f** (Figure 6). A total of 13,797 genes were detected, of which 947 and 656 were differentially upregulated with statistical significance (FDR >0.05, and fold change [FC] > 2) in the TDB and **4f**-treated cells, respectively. Further hierarchical clustering analysis of the differentially expressed genes (DEGs) revealed four clusters of expression patterns (A–D) (Figures 6A,B) (Ge et al., 2018). KEGG pathway enrichment of cluster A and B revealed 15 significantly enriched pathway terms in each cluster. The majority of the pathway terms were linked to inflammation and immunity, including cytokine-cytokine receptor interaction, TNF signaling pathway, NF-κB signaling pathway, IL-17 signaling pathway, MAPK signaling pathway and C-type lectin receptor signaling pathway (Figure 6B). Further analysis of DEGs between the **4f** and TDB treated groups revealed the relative down-regulation of pathways associated with phagocytosis (Figure 6C), including ribosome, phagosome (including FcRγ-mediated phagocytosis) and lysosome pathways, along with regulation of the actin cytoskeleton and focal adhesion (Luo et al., 2017).

## 4 Discussion

The desire to understand how the structure of trehalose glycolipids influences their immunomodulatory activity has been a topic of much interest in recent years (Foster et al., 2018; Ryter et al., 2020; Dangerfield et al., 2022). In particular, the poor solubility of long chain trehalose diesters potentially decreases their attractiveness as adjuvant candidates and has led to interest in more water-soluble adducts (Jacobsen et al., 2015; Huber et al., 2016; O’Hagan et al., 2020). One way to increase the water-solubility of trehalose glycolipids is to include polyethylene glycol (PEG) groups along the backbone of the lipid, with Lang and co-workers adopting this approach to produce 6,6′-bis [8-(stearoylamido)-3,6-dioxaoctanoyl]-trehalose (PEG-TDS **3**) (Huber et al., 2016). Unfortunately, this adduct exhibited only modest Mincle-binding, and was not a promising Mincle adjuvant.

We reasoned that by incorporating PEG groups at the terminus of the trehalose glycolipid acyl chains, we could develop Mincle ligands (PEG-TGLs) that would not only be more water-soluble but would also retain good Mincle binding since the lipophilic acyl portion of the PEG-TGL would be sufficiently apolar to interact with the hydrophobic groove of Mincle (Feinberg et al., 2013; Furukawa et al., 2013; Feinberg et al., 2016). To this end, we successfully synthesized six PEGylated trehalose glycolipids **4a-f** with improved water-solubility, as illustrated by the calculated LogP values of 3.16–8.09 (compared to TDB with a CLogP = 18.39), and these adducts were then tested for their ability to signal through mMincle using GFP reporter cells. All PEG-TGLs, except the most water-soluble adduct **4c**, exhibited good mMincle signaling, thus confirming our hypothesis that through the judicious placement of the PEG groups on the acyl chain, more water-soluble trehalose glycolipids that maintain strong Mincle binding could be prepared. Both the hydrophilicity and length of the lipophilic portion engaging with Mincle affected the ensuing immune response with a longer lipophilic portion in the 6- and 6′-acyl chains (i.e., *n* = 14 vs. *n* = 10) tending to enhance Mincle signaling, while an increase in hydrophilicity (i.e., an increase in the number of ethylene glycol units) led to a slight decrease in Mincle signaling where *n* = 14.

In contrast, the poor ability of the PEG-TGLs to lead to the production of cytokines by GM-CSF treated BMCs was unexpected. While we anticipated that **4c**, which exhibited poor mMincle signaling, would elicit a poor functional response, as previously reported in other studies (Huber et al., 2016; Foster et al., 2018), we were surprised to observe that PEG-TGL **4f**, which had a stronger affinity for mMincle than TDB (**2**), also led to modest cytokine production. Knowing that assay set-up could affect the ensuing immune response (Stocker et al., 2019), we performed both ligand-coated and ligand solubilized assays. However, on the whole, the response to the PEG-TGLs was modest compared to TDB and less pro-inflammatory in nature. Notably, no IL-1β, a cytokine indicative of promising adjuvanticity (Nakae et al., 2001), was observed. Given that macrophage, rather than DC, are responsible for inflammatory activity the GM-CSF model (Erlich et al., 2019), we can assume that it is macrophage, and not DCs, that effect IL-1β production in our experimental set-up.

The distinct lack of IL-1β production in response to **4f** and the other PEG-TGLs prompted us to consider the mechanism by which **4f** and TDB lead to IL-1β production. Although we, and others, had previously determined that glycolipid presentation can influence the immune response to trehalose glycolipids (Kallerup et al., 2017; Stocker et al., 2019), the molecular mechanisms for this were not investigated. Moreover, we had previously observed that short-chain trehalose glycolipids led to a less inflammatory immune response (Khan et al., 2011; Khan et al., 2018), though had assumed that this might be related to poor Mincle binding. However, our data in this study indicated that lipid binding was not the only consideration for IL-1β production.

Two signals are required for inflammasome activation and IL-1β production. Mincle binding is a prerequisite for induction of the FcRγ-Syk-Card9 signaling axis which mediates the TDB- or TDM-induced signaling for NF-κB activation and the production of pro-inflammatory cytokines (Ishikawa et al., 2009; Werninghaus et al., 2009; Schoenen et al., 2010). Insomuch, this constitutes signal-1 for inflammasome activation. Signal-2 of NLPR3 inflammasome activation by TDB has been reported to require phagocytosis, lysosomal acidification, and cathepsin activity, with the particulate nature of TDB believed to be key in this process (Schweneker et al., 2013). While this may indicate that the induction of phagocytic pathways plays an essential role in the ability of trehalose glycolipids to generate an inflammatory immune response, there are many ways by which the inflammasome can be activated (Swanson et al., 2019; Zheng et al., 2020).

**FIGURE 7:**
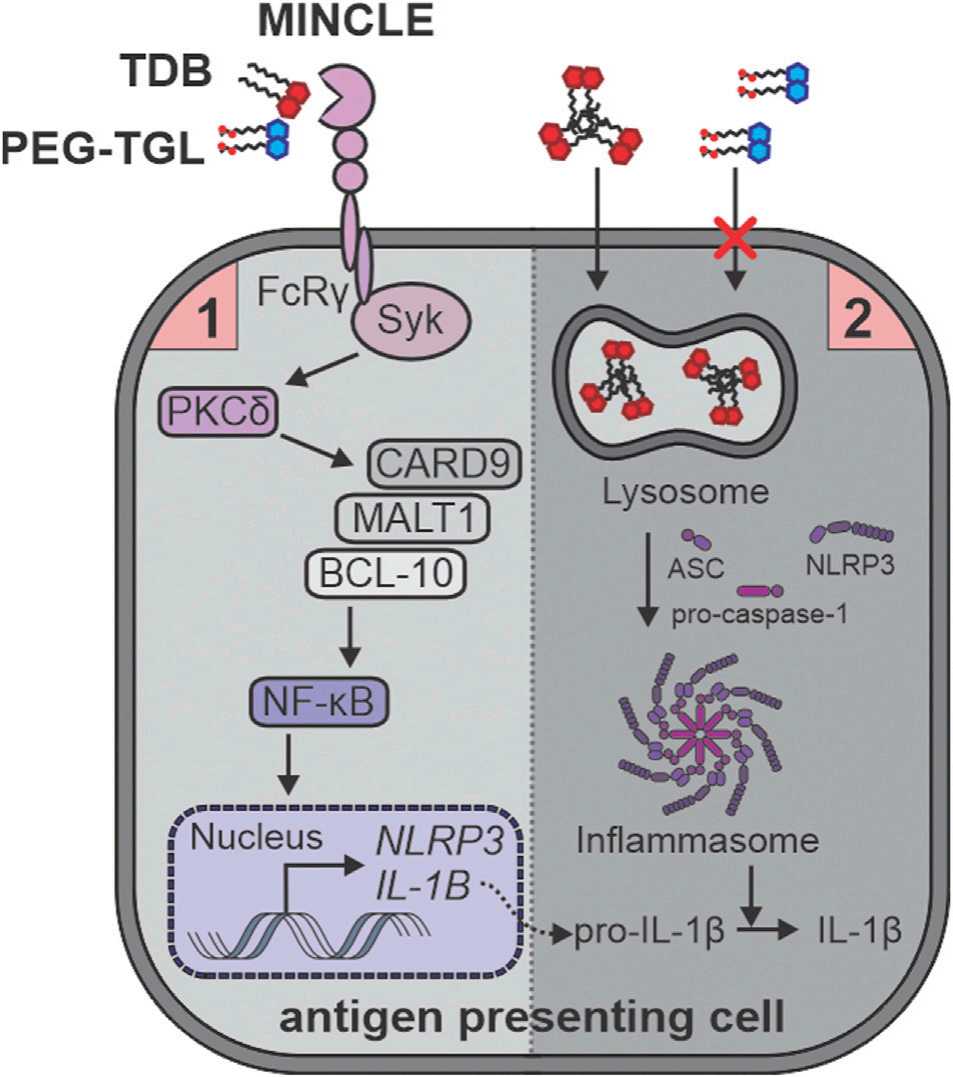
TDB (**2**), but not PEG-TGL (**4f**), leads to lysosome-mediated activation of the inflammasome and IL-1b production. TDB (**2**) and PEG-TGL (**4f**) are both bind to and signal *via* Mincle, thus providing the priming signal-1 for inflammasome activation. Only TDB (**2**) can sufficiently induce phagocytosis, and thus, signal-2 for inflammasome activation. This leads to TDB (**2**), but not PEG-TGL (**4f**), mediated IL-1b production.

To understand the mechanistic rationale behind the ability of TDB, and to a lesser extent, PEG-TGL **4f**, to activate GM-CSF treated BMCs, we used RNA sequencing to analyze the transcriptome control of immune cell activation pathways. Lang and co-workers had explored the transcriptome responses by wildtype and Mincle^−/−^ bone marrow derived macrophages (BMDMs) in response to TDM and TDB and revealed a variety of Mincle-dependent and Mincle-independent pathways that were associated with the innate immune responses and cell cycle regulation, respectively (Hansen et al., 2019). The Mincle-dependent pathways included strong enrichment for endo/lysosomal and transport proteins, a finding that we also observed upon the treatment of GM-CSF treated BMCs with TDB. In contrast, compared to TDB, PEG-TGL **4f** did not lead to similar upregulation of Fcγ-R-mediated phagocytosis or associated pathways such as phagosome, lysosome, and actin-cytoskeleton regulation. Taken together, this suggests that the water-soluble nature of PEG-TGL **4f** fails to initiate the phagocytosis-mediated signal-2 of inflammasome activation, which in turn, prevents the cleavage of pro-IL-1β into IL-1β (Figure 7).

With these findings in mind, it begs the question whether PEG-TGL **4f**, or indeed, any water-soluble trehalose glycolipid, would make a good adjuvant? Strong Mincle binding is clearly a pre-requisite for good Mincle-mediated cellular responses, and ligands need to be designed to ensure that the Mincle binding affinity is retained. Following this, understanding how Mincle ligands activate the inflammasome, and hence, the production of IL-1β, is more complex. It has been proposed that TDM and TDB lead to the multimerization of Mincle and other related CLRs, such as the macrophage C-type lectin (MCL), with this multimerization enhancing inflammatory gene responses and phagocytosis (Lobato-Pascual et al., 2013; Yamasaki, 2013). Mincle has also recently been shown to mediate self-lipid endocytosis (Kostarnoy et al., 2022). However, our data demonstrated that any such Mincle multimerization does not sufficiently engage the Fcγ-R-mediated phagocytotic pathway that is needed for IL-1β production in response to trehalose glycolipids, and that colloids, rather than the coordinated properties of discrete compounds, are required for a good pro-inflammatory response to Mincle ligands.

It is difficult to say whether water-solubility can be used as a proxy for inflammasome activation by trehalose glycolipids, however, this is something to be considered when designing new Mincle agonists. The lack of IL-1β production in response to short-chain trehalose glycolipids (CLogP = 8.61, for iso-C12 + 1) supports the notion that water-solubility is related to inflammasome activation (Khan et al., 2011; Khan et al., 2018), although the monoester of TDB (CLogP = 7.56) has been reported to lead to IL-1β by macrophages (Stocker et al., 2014). Notwithstanding, the immune responses towards the mono-esters are generally poorer than those elicited by their diester counterparts (Huber et al., 2016; Bird et al., 2018). Whether the delivery of water-soluble ligands with particulate adjuvants, such as alum (Eisenbarth et al., 2008), or in liposomal formulations (Kallerup et al., 2017), can be used to develop effective water-soluble Mincle ligands also remains to be seen.

With the tremendous amount of synthetic effort that is put into the design and synthesis of Mincle agonists (Williams, 2017; Braganza et al., 2018), it is of value to have more guidance as it relates to optimizing Mincle ligands for their potential adjuvanticity. To this end, we have demonstrated that both Mincle binding, and the ability of the ligands to activate the inflammasome, through mechanisms that are presumably related to the particulate nature of the substrates, are important. Indeed, it may well be that the amphiphilic nature of trehalose glycolipids, which is what makes them somewhat water insoluble and which can complicate the interpretation of experiments, plays a critical role in their effectiveness as adjuvants. There also appears to be a fine balance between improved water solubility and retention of the pro-inflammatory activity of this class of compound. Accordingly, when screening for Mincle agonists, it is suggested that Mincle ligand binding and signaling, along with the use of the appropriate functional assays, are undertaken to better allow for the identification of lead compounds.

## Data Availability

The datasets presented in this study can be found in online repositories. The names of the repository/repositories and accession number(s) can be found below: https://www.ncbi.nlm.nih.gov/, GSE210869.
