# Supplementary material for: Water-soluble trehalose glycolipids show superior Mincle binding and signaling but impaired phagocytosis and IL-1β production
Source: Front Mol Biosci. 2022 Nov 24;9:1015210. doi: 10.3389/fmolb.2022.1015210 (PMC9729344; doi:10.3389/fmolb.2022.1015210)
Supplement: Supplementary file 1 [file DataSheet1.PDF]

Supplementary Material

1 Chemical Synthesis

1.1 General Methods

Unless otherwise stated, all reactions were performed under an argon atmosphere. Distilled methanol was used in reactions. THF (Romil) was distilled over sodium/benzophenone under an atmosphere of argon prior to use in reactions. EtOAc (Pure Science), and petroleum ether (Pure Science) were distilled prior to use. Hydrobromic acid (33% in AcOH, Acros), H_2SO_4 (Pure Science), 60% or 90% NaH in mineral oil (Aldrich), TBAI (Riedel-de Haen), NaI (Pure Science), *N*, *O*-bis(trimethylsilyl)acetamine (Aldrich), TBAF (Acros), K_2CO_3 (Panreac), EDCI (Chem Impex), DMAP (Lab Supply), toluene (ROMIL), CH_2Cl_2 (Fischer Scientific), $CDCl_3$ (Aldrich) and CD_3OD (Cambridge Isotope Laboratories) were used as received. All solvents were evaporated under reduced pressure. Reactions were monitored by TLC-analysis using Macherey-Nagel silica gel pre-coated plastic sheets (0.20 mm, fluorescent indicator UV-254) and visualised under UV absorption (254 nm), dipping in 10% H_2SO_4 in EtOH followed by charring, dipping in ceric ammonium molybdate solution or dipping in $KMnO_4$ solution (2% in H_2O). Column chromatography was performed using Pure Science silica gel (40-63 µm). High resolution mass spectroscopic data were obtained on an Agilent 6530 Q-TOF mass spectrometer with a JetStream™ electrospray ionisation source in positive or negative mode. Optical rotations were recorded on an Autopol II (Rudolph Research Analytical) at 589 nm (sodium D-line). Infrared spectra were obtained for the compounds (as a thin film) using a Bruker Platinum ATR and are reported in wave numbers (cm^{-1}). Nuclear magnetic resonance spectra were recorded at 20 °C in $CDCl_3$ or CD_3OD using a JEOL JNM-ECZ spectrometer operating at 500 or 600 MHz. Chemical shift values given in ppm (δ) are relative to residual solvent peaks. Peak assignments in NMR spectra were made using 2D-NMR experiments: COSY, HSQC, and HMBC.

1.2 Synthetic procedures:

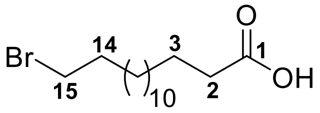

6

15-Bromopentadecanoic acid (6): To 15-cyclopentadecanolide (**5**, 2.02 g, 8.39 mmol) were added HBr (33% in AcOH, 7 mL) and H_2SO_4 (1 mL) and the mixture was refluxed for 5 hours. The resulting orange coloured reaction mixture was then cooled to room temperature before being diluted with water (30 mL) and Et_2O (50 mL). The organic layer was separated, and the aqueous layer was again extracted with Et_2O (50 mL). The combined organic layers were washed with brine (100 mL), dried over anhydrous $MgSO_4$, filtered, and concentrated *in vacuo*. The resulting yellow-white solid residue was recrystallized from CH_2Cl_2/Pet. Ether to give **6** as white crystals (2.09 g, 6.50 mmol, 78%). R_f = 0.59 (Pet. Ether:Et_2O, 7:3, *v/v*); ^{1}H NMR (500 MHz, $CDCl_3$) δ 3.41 (t, $J_{14,15}$ = 6.9 Hz, 2H, H-15), 2.35 (t, $J_{2,3}$ = 7.5 Hz, 2H, H-2), 1.88–1.82 (m, 2H, H-14), 1.63 (p, $J_{2,3}$ = $J_{3,4}$ = 7.5 Hz, 2H, H-3), 1.45–1.39 (m, 2H, H-13), 1.36–1.23 (m, 18H, H-4–H-12); ^{13}C NMR (125 MHz, $CDCl_3$) δ 179.7 (C-1), 34.2 (C-15), 34.1 (C-2), 33.0 (C-14), 29.73, 29.71, 29.67, 29.58, 29.56, 29.4, 29.2, 28.9 (C-4–C-12), 28.3 (C-13), 24.8 (C-3); IR (film): 3000 (broad), 2916, 2849, 1701, 1472, 1463, 1436, 1410, 1300, 1280, 1258, 1236, 1217, 1199, 909, 731, 720, 650 cm^{-1}; HRMS (ESI) *m/z* calcd. for $[C_{15}H_{29}BrO_2\text{-}H]^-$: 319.1278; obsd.: 319.1287. Data recorded matched that available in the literature [1].

General procedure for Williamson ether synthesis: To NaH (2.5 equiv.) in DMF or THF (1.5-2 mL/mmol) at 0 °C, was added the corresponding glycol ether (1 equiv.) in DMF or THF (2.5-3 mL/mmol) and the resulting suspension was stirred for 30 minutes. To the stirred mixture was added TBAI or NaI (0.1 equiv.), followed by the corresponding halo-carboxylic acid (1 equiv.) dissolved in DMF or THF (2.5-3 mL/mmol) and the resulting solution was allowed to slowly come to room temperature and/or slowly heated to 55 °C until the reaction is no longer vigorous and no more bubbling was observed. Completion of the reaction was confirmed, as gauged by TLC, and the reaction mixture was then quenched with water followed by careful addition of 1M HCl, and the solution was then concentrated under reduced pressure. The residue was separated between water and Et_2O or EtOAc. The organic layer was separated, and the aqueous phase was again extracted with Et_2O or EtOAc. The combined organic layers were dried over anhydrous $MgSO_4$, filtered and concentrated under reduced pressure.

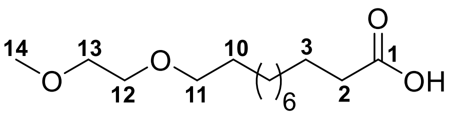

9a

11-(2-Methoxyethoxy)undecanoic acid (9a): Bromo-carboxylic acid **8** (0.500 g, 1.79 mmol) was subjected to the general procedure for Williamson ether synthesis with ethylene glycol monomethyl ether (**7a**, 0.14 mL, 1.79 mmol), 90% NaH (129 mg, 4.84 mmol) and NaI (270 mg, 0.180 mmol) in DMF. The reaction was deemed complete after 20 h at 55 °C, as gauged by TLC (Pet. Ether:EtOAc, 1:1, *v/v*, and AcOH 0.05 mL/10 mL). Following work-up (extraction with EtOAc) and purification using gradient silica gel flash chromatography (eluting with Pet. Ether:EtOAc, 1:0→7:3, *v/v*) the title compound **9a** was obtained as a colourless viscous oil (100 mg, 0.384 mmol, 20%). R_f = 0.55 (Pet. Ether:EtOAc, 1:1, *v/v*); ^{1}H NMR (500 MHz, $CDCl_3$) δ 3.59–3.53 (m, 4H, OCH_2CH_2O), 3.45 (t, $J_{10,11}$ = 6.9 Hz, 2H, H-11), 3.39 (s, 3H, H-14), 2.35 (t, $J_{2,3}$ = 7.5 Hz, 2H, H-2), 1.66–1.56 (m, 4H, H-3 and H-10), 1.34–1.25 (m, 12H, H-4–H-9); ^{13}C NMR (125 MHz, $CDCl_3$) δ 179.5 (C-1), 72.1 (C-13), 71.8 (C-11), 70.1 (C-12), 59.2 (C-14), 34.1 (C-2), 29.7 (C-10), 29.56, 29.52, 29.4, 29.3, 29.1, 26.2 (C-4–C-9), 24.8 (C-3); IR (film): 3000 (broad), 2924, 2854, 1735, 1707, 1457, 1412, 1357, 1283, 1242, 1198, 1106, 1031, 939, 849, 723 cm^{-1}; HRMS (ESI) *m/z* calcd. for $[C_{14}H_{28}O_4+H]^+$: 261.2060; obsd.: 261.2064.

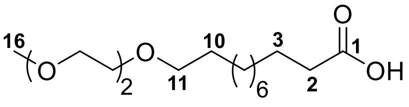

9b

11-(2-(2-Methoxyethoxy)ethoxy)undecanoic acid (9b): 11-Bromoundecanoic acid (**8**, 1.00 g, 3.77 mmol) was subjected to the general procedure for Williamson ether synthesis with diethylene glycol monomethyl ether (**7b**, 0.45 mL, 3.8 mmol) and 60% NaH (377 mg, 9.43 mmol) using DMF as a solvent. The reaction was deemed complete after stirring for 48 h at room temperature, as gauged by TLC (Pet. Ether:EtOAc, 1:1, *v/v* and AcOH 0.05 mL/10 mL). Following work-up (extraction with Et_2O) and purification using gradient silica gel flash chromatography (Pet. Ether:EtOAc, 1:0→3:2, *v/v*), the title compound **9b** was obtained as a colourless viscous oil (339 mg, 1.11 mmol, 30%). R_f = 0.42 (Pet. Ether:EtOAc, 2:3, *v/v*); ^{1}H NMR (500 MHz, $CDCl_3$) δ 3.65–3.61 (m, 4H, OCH_2CH_2O), 3.60–3.57 (m, 2H, OCH_2CH_2O), 3.56–3.54 (m, 2H, OCH_2CH_2O), 3.44 (t, $J_{10,11}$ = 6.8 Hz, 2H, H-11), 3.37 (s, 3H, H-16), 2.32 (t, $J_{2,3}$ = 7.5 Hz, 2H, H-2), 1.63–1.53 (m, 4H, H-3 and H-10), 1.35–1.26 (m, 12H, H-4–H-9); ^{13}C NMR (125 MHz, $CDCl_3$) δ 179.6 (C-1), 72.1(C-15), 71.6 (C-11), 70.7, 70.6 (C-13 and C-14, 70.1 (C-12), 59.1 (C-16), 34.1 (C-2), 29.7, 29.6, 29.5, 29.4, 29.3, 29.1, 26.1 (C-4–C-10), 24.8 (C-3); IR (film): 3000 (broad), 2924, 2854, 1734, 1708, 1458, 1352, 1284, 1243, 1199, 1103, 1028, 933, 849, 722 cm^{-1}; HRMS (ESI) *m/z* calcd. for $[C_{16}H_{32}O_5+NH_4]^+$: 322.2588; obsd.: 322.2587.

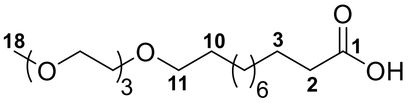

9c

11-(2-(2-(2-Methoxyethoxy)ethoxy)ethoxy)undecanoic acid (9c): 11-Bromoundecanoic acid (**8**, 190 mg, 0.716 mmol), triethylene glycol monomethyl ether (**7c**, 0.113 mL, 0.716 mmol) and 60% NaH (86 mg, 2.2 mmol) were subjected to the general procedure for the Williamson ether synthesis using THF as the solvent. The reaction was deemed complete after stirring for 22 h at room temperature, as gauged by TLC (Pet. Ether:EtOAc, 1:1, *v/v*, and AcOH 0.05 mL/10 mL). Following work-up (extraction with EtOAc) and purification via silica gel flash column chromatography using gradient elution (Pet. Ether:EtOAc, 1:0→2:3, *v/v*), the title compound **9c** was obtained as a colourless viscous oil (85.7 mg, 0.244 mmol, 34%). R_f = 0.16 (Pet. Ether:EtOAc, 1:1, *v/v*); ^{1}H NMR (500 MHz, $CDCl_3$) δ 3.65–3.61 (m, 8H, OCH_2CH_2O), 3.57–3.52 (m, 4H, OCH_2CH_2O), 3.42 (t, $J_{10,11}$ = 6.8 Hz, 2H, H-11), 3.36 (s, 3H, H-18), 2.30 (t, $J_{2,3}$ = 7.5 Hz, 2H, H-2), 1.63–1.56 (m, 2H, H-3), 1.57–1.51 (m, 2H, H-10), 1.32–1.25 (m, 12H, H-4–H-9); ^{13}C NMR (125 MHz, $CDCl_3$) δ 179.3 (C-1), 72.0 (C-17), 71.6 (C-11), 70.66, 70.63, 70.5 (C-13–C-16), 70.1 (C-12), 59.1 (C-18), 34.1 (C-2), 29.6 (C-10), 29.54, 29.47, 29.39, 29.26, 29.1, 26.1 (C-4–C-9), 24.8 (C-3); IR (film): 3150 (broad), 2924, 2854, 1734, 1708, 1458, 1351, 1285, 1245, 1199, 1104, 942, 850, 722 cm^{-1}; HRMS (ESI) *m/z* calcd. for $[C_{18}H_{36}O_6+H]^+$: 349.2585; obsd.: 349.2596.

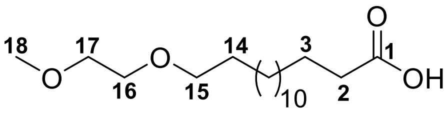

9d

15-(2-Methoxyethoxy)pentadecanoic acid (9d): NaH (90%, 74 mg, 2.8 mmol), 2-methoxyehtanol (**7a**, 0.09 mL, 1.1 mmol), TBAI (42 mg, 0.11 mmol) and 15-bromopentadecanoic acid (**6**, 259 mg, 0.806 mmol, co-evaporated with 2 × 3 mL of DMF), were subjected to the general procedure for the Williamson ether synthesis reaction in DMF at 55 °C. The reaction was deemed complete by TLC after 3 days (Pet. Ether:EtOAc, 3:2, *v/v*, and AcOH 0.05 mL/10 mL). Following work-up (extraction with Et_2O) and purification via gradient silica gel flash column chromatography (Pet. Ether:EtOAc, 1:0→3:1, *v/v*), the title compound **9d** was obtained as a white solid (119 mg, 0.376 mmol, 47%). R_f = 0.46 (Pet. Ether:EtOAc, 3:2, *v/v*); ^{1}H NMR (500 MHz, $CDCl_3$) δ 3.59–3.53 (m, 4H, OCH_2CH_2O), 3.45 (t, $J_{14,15}$ = 6.9 Hz, 2H, H-15), 3.39 (s, 3H, H-18), 2.34 (t, $J_{2,3}$ = 7.5 Hz, 2H, H-2), 1.66–1.56 (m, 4H, H-3 and H-14), 1.34–1.25 (m, 20H, H-4–H-13); ^{13}C NMR (125 MHz, $CDCl_3$) δ 179.2 (C-1), 72.1 (C-17), 71.8 (C-15), 70.1 (C-16), 59.2 (C-18), 34.0 (C-2), 29.72, 29.69, 29.66, 29.61, 29.5, 29.3, 29.2, 26.2 (C-4–C-14), 24.8 (C-3); IR (film): 3000 (broad), 2914, 2849, 1691, 1448, 1332, 1314, 1272, 1251, 1210, 1142, 947, 718 cm^{-1}; HRMS (ESI) *m/z* calcd. for $[C_{18}H_{36}O_4+H]^+$: 317.2686; obsd.: 317.2686.

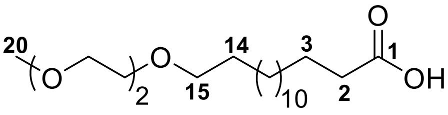

9e

15-(2-(2-Methoxyethoxy)ethoxy)pentadecanoic acid (9e): 15-Bromopentadecanoic acid (**6**, 753 mg, 2.34 mmol, co-evaporated with 2 × 6 mL of DMF) and diethylene glycol monomethyl ether (**7b**, 0.28 mL, 2.4 mmol) were subjected to the general procedure for the Williamson ether synthesis in the presence of 90% NaH (160 mg, 5.99 mmol) and TBAI (89.5 mg, 0.242 mmol) at 55 °C. The reaction was deemed complete after 2 days, as gauged by TLC (Pet. Ether:EtOAc, 3:2, *v/v*, and AcOH 0.05 mL/10 mL). Following work-up (extraction with Et_2O) and purification via gradient silica gel flash column chromatography (Pet. Ether:EtOAc, 1:0→7:3, *v/v*), the target product **9e** was obtained as a white solid (345 g, 0.956 mmol, 41%). R_f = 0.39 (Pet. Ether:EtOAc, 1:1, *v/v*); ^{1}H NMR (500 MHz, $CDCl_3$) δ 3.66–3.63 (m, 4H, OCH_2CH_2O), 3.61–3.58 (m, 2H, OCH_2CH_2O), 3.57–3.55 (m, 2H, OCH_2CH_2O), 3.45 (t, $J_{14,15}$ = 6.8 Hz, 2H, H-15), 3.38 (s, 3H, H-20), 2.34 (t, $J_{2,3}$

= 7.5 Hz, 2H, H-2), 1.65–1.54 (m, 4H, H-3 and H-14), 1.36–1.25 (m, 20H, H-4–H-13); ^{13}C NMR (125 MHz, $CDCl_3$) δ 179.3 (C-1), 72.1 (C-19), 71.7 (C-15), 70.8, 70.6 (C-17 and C-18), 70.2 (C-16), 59.2 (C-20), 34.1 (C-2), 29.7, 29.6, 29.5, 29.3, 29.2, 26.2 (C-4–C-14), 24.8 (C-3); IR (film): 2950 (broad), 2915, 2848, 1699, 1471, 1463, 1313, 1295, 1120, 943, 728, 720 cm^{-1}; HRMS (ESI) *m/z* calcd. for $[C_{20}H_{40}O_5+NH_4]^+$: 378.3214; obsd.: 378.3219.

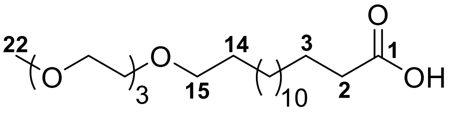

9f

15-(2-(2-(2-Methoxyethoxy)ethoxy)ethoxy)pentadecanoic acid (9f): 15-Bromopentadecanoic acid (**6**, 258 mg, 0.803 mmol) was co-evaporated with DMF (2× 3 mL) and subjected to the general procedure for the Williamson ether synthesis with 90% NaH (74 mg, 1.6 mmol), triethylene glycol monomethyl ether (**7c**, 0.23 mL, 1.1 mmol) and TBAI (39.7 mg, 0.107 mmol). The reaction was deemed complete after 4 days, as gauged by TLC (Pet. Ether:EtOAc, 3:2, *v/v*, and AcOH 0.05 mL/10 mL). Following work-up (extraction with Et_2O) and purification by gradient silica gel flash column chromatography (eluted with Pet. Ether:EtOAc, 1:0→3:7, *v/v*), the title compound **9f** was obtained as a white solid (202 mg, 0.500 mmol, 62%); R_f = 0.35 (Pet. Ether:EtOAc, 11:9, *v/v*); ^{1}H NMR (500 MHz, $CDCl_3$) δ 3.68–3.62 (m, 8H, OCH_2CH_2O), 3.58–3.53 (m, 4H, OCH_2CH_2O), 3.44 (t, $J_{14,15}$ = 6.8 Hz, 2H, H-15), 3.37 (s, 3H, H-22), 2.32 (t, $J_{2,3}$ = 7.5 Hz, 2H, H-2), 1.64–1.53 (m, 4H, H-3 and H-14), 1.35–1.20 (m, 20H, H-4–H-13); ^{13}C NMR (125 MHz, $CDCl_3$) δ 179.4 (C-1), 71.7 (C-15), 72.0, 70.73, 70.69, 70.61 (C-17–C-21), 70.1 (C-16), 59.1 (C-22), 34.1 (C-2), 29.72, 29.69, 29.66, 29.59, 29.52, 29.3, 29.2, 26.2 (C-4–C-14), 24.8 (C-3); IR (film): 3100 (broad), 2914, 2868, 2848, 1707, 1689, 1471, 1309, 1227, 1208, 1143, 1125, 947, 718 cm^{-1}; HRMS (ESI) *m/z* calcd. for $[C_{22}H_{44}O_6+NH_4]^+$: 422.3476; obsd.: 422.3475.

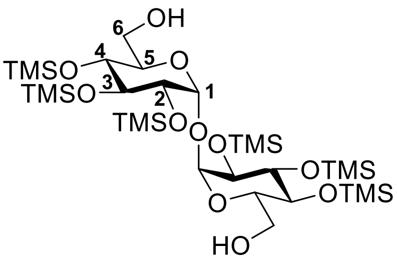

10

2,2´,3,3´,4,4´-Hexa-*O*-trimethylsilyl-α,α´-D-trehalose (10): α,α′-D-Trehalose dihydrate (2.05 g, 5.42 mmol) was co-evaporated with DMF (2 x 16 mL), and the third time with excess DMF to reduce volume to half (8 mL). To this was added BSA (11.5 mL, 47 mmol), followed by TBAF (0.33 mL, 1.1 mmol). After being stirred at room temperature for 2 h, the reaction mixture was then quenched with isopropanol (2 mL) and diluted with 100 mL of MeOH. To this, was added K_2CO_3 (730 mg, 5.28 mmol) at 0 °C and the reaction stirred for a further 2 h. Upon completion (as gauged by TLC in Pet. Ether:EtOAc, 3:1, *v/v*), the reaction mixture was neutralised with AcOH and concentrated under reduced pressure. The residue was partitioned between diethyl ether (2 x 90 mL) and brine (90 mL) and the combined organic layers were dried over $MgSO_4$ and the solvent evaporated *in vacuo*. The resulting crude product was purified via gradient silica gel flash column chromatography (Pet. Ether:EtOAc, 1:0→4:1, *v/v*) to give the product as a white solid (3.17 g, 4.09 mmol, 76%). Data obtained for the compound matched that in the literature [2,3].

General procedure for esterification: Carboxylic acid (4 equiv.) and diol (1 equiv.) were co-evaporated with dry toluene (2 × 30-40 mL/mmol) and then dissolved in dry toluene (25-30 mL/mmol). EDCI (6 equiv.) and DMAP (1 equiv.) were added to the reaction mixture, which was then stirred at 70 °C overnight. Additional reagents were added as necessary and as detailed in the individual procedures. Once the reaction was completed, as gauged by TLC analysis, the reaction mixture was cooled to room temperature, diluted with EtOAc (30-40 mL),

washed with water (30-40 mL) and a saturated aqueous solution of NaCl (30-40 mL), dried over $MgSO_4$, filtered, and concentrated under reduced pressure.

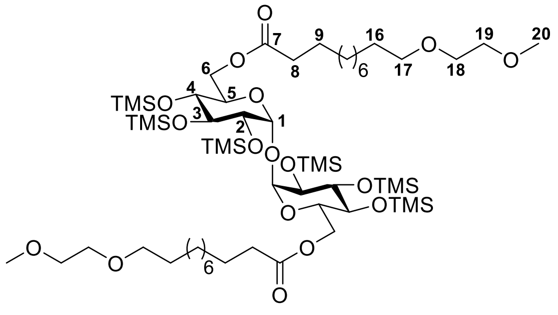

11a

6,6′-Di-*O*-(11-(2-methoxyethoxy)undecanoyl)-2,2′,3,3′,4,4′-hexa-*O*-trimethylsilyl-1,1′-α,α′-D-trehalose (11a): Diol **10** (77.0 mg, 0.099 mmol) was subjected to the general procedure for esterification with acid **9a** (94.0 mg, 0.361 mmol), EDCI (111 mg, 0.579 mmol) and DMAP (17.7 mg, 0.145 mmol), and stirred for 54 h. Following work-up and purification using gradient silica gel flash chromatography (eluting with Pet. Ether:EtOAc, 1:0→7:3, *v/v*), the title compound **11a** was obtained as a colourless viscous oil (87.9 mg, 0.070 mmol, 72%). R_f = 0.47 (Pet. Ether:EtOAc, 7:3, *v/v*); $[\alpha]_D^{23.3}$ = +60 (*c* = 0.1, CH_2Cl_2); ^1H NMR (500 MHz, $CDCl_3$) δ 4.91 (d, $J_{1,2}$ = 3.1 Hz, 2H, H-1), 4.27 (dd, $J_{6a,b}$ = 11.9 Hz, $J_{5,6a}$ = 2.3 Hz, 2H, H-6a), 4.05 (dd, $J_{6a,b}$ = 11.9 Hz, $J_{5,6b}$ = 4.4 Hz, 2H, H-6b), 3.99 (ddd, $J_{4,5}$ = 9.5 Hz, $J_{5,6b}$ = 4.4 Hz, $J_{5,6a}$ = 2.3 Hz, 2H, H-5), 3.90 (t, $J_{2,3}$ = $J_{3,4}$ = 9.0 Hz, 2H, H-3), 3.58–3.52 (m, 8H, OCH_2CH_2O), 3.49–3.41 (m, 8H, H-2, H-4 and H-17), 3.38 (s, 6H, H-20), 2.38–2.28 (m, 4H, H-8), 1.64–1.55 (m, 8H, H-9 and H-16), 1.34–1.26 (m, 24H, H-10–H-15), 0.15, 0.13, 0.12 (3s, 54H, CH_3, TMS); ^{13}C NMR (125 MHz, $CDCl_3$) δ 173.9 (C-7), 94.6 (C-1), 73.6 (C-3), 72.8 (C-2), 72.0 (C-4), 71.8 (C-17), 70.9 (C-5), 72.2, 70.1 (C-18 and C-19), 63.4 (C-6), 59.2 (C-20), 34.3 (C-8), 29.73, 29.68, 29.59, 29.55, 29.4, 29.3, 26.2 (C10–C-16), 24.9 (C-9), 1.2, 1.0, 0.3 (TMS); IR (film): 2926, 2855, 1740, 1250, 1163, 1109, 1075, 1045, 1008, 897, 870, 836, 747 cm^{-1}; HRMS (ESI) *m/z* calcd. for $[C_{58}H_{122}O_{17}Si_6+NH_4]^+$: 1276.7636; obsd.: 1276.7659.

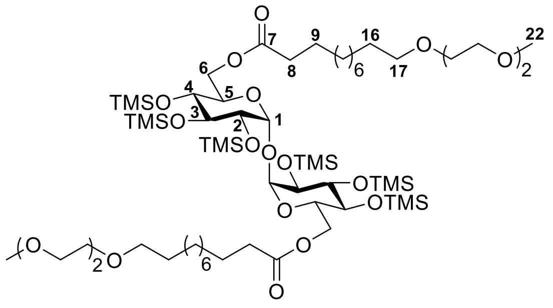

11b

6,6′-Di-*O*-(11-(2-(2-methoxyethoxy)ethoxy)undecanoyl)-2,2′,3,3′,4,4′-hexa-*O*-trimethylsilyl-1,1′-α,α′-D-trehalose (11b): TMS-protected diol **10** (100 mg, 0.129 mmol) was subjected to the general esterification procedure with glycol functionalised carboxylic acid **9b** (157 mg, 0.516 mmol), EDCI (148 mg, 0.772 mmol) and DMAP (15.8 mg, 0.129 mmol), and the reaction was stirred for 54 h. Following work-up and purification via gradient silica gel flash chromatography (eluting with Pet. Ether:EtOAc, 1:0→3:2, *v/v*), the title compound **11b** was obtained a colourless viscous oil (142 mg, 0.105 mmol, 82%). R_f = 0.55 (Pet. Ether:EtOAc, 1:1, *v/v*); $[\alpha]_D^{22.2}$ = +60 (*c* = 0.1, CH_2Cl_2); ^1H NMR (500 MHz, $CDCl_3$) δ 4.91 (d, $J_{1,2}$ = 3.1 Hz, 2H, H-1), 4.27 (dd, $J_{6a,b}$ = 12.0 Hz, $J_{5,6a}$ = 2.3 Hz, 2H, H-6a), 4.05 (dd, $J_{6a,b}$ = 11.9 Hz, $J_{5,6b}$ = 4.3 Hz, 2H, H-6b), 3.99 (ddd, $J_{4,5}$ = 9.5 Hz, $J_{5,6b}$ = 4.3 Hz, $J_{5,6a}$ = 2.3 Hz, 2H, H-5), 3.90 (t, $J_{2,3}$ = $J_{3,4}$ = 8.9 Hz, 2H, H-3), 3.66–3.63 (m, 8H, OCH_2CH_2O), 3.60–3.58 (m, 4H, OCH_2CH_2O), 3.56–3.54 (m, 4H, OCH_2CH_2O), 3.49–3.42 (m, 8H, H-2, H-4 and H-17), 3.38 (s, 6H, H-22), 2.38–2.28 (m, 4H, H-8), 1.64–1.54 (m, 8H, H-9–H-16), 1.34–1.26 (m, 24H, H-10–H-15), 0.14, 0.13, 0.12 (3s, 54H, CH_3, TMS); ^{13}C NMR (125 MHz, $CDCl_3$) δ 173.9 (C-7), 94.6 (C-1), 73.6 (C-3), 72.8 (C-2), 72.02 (C-4), 71.7 (C-17), 70.85 (C-5), 72.09, 70.80, 70.7, 70.2 (C-18–C-21), 63.4 (C-6), 59.2 (C-22), 34.3 (C-8), 29.8, 29.69, 29.60, 29.55, 29.45, 29.3, 26.2, 24.9 (C-10–

C-16), 1.2, 1.0, 0.3 (TMS); IR (film): 2927, 2856, 1740, 1250, 1145, 1109, 1075, 1045, 1008, 897, 871, 837, 747 cm^{-1}; HRMS (ESI) *m/z* calcd. for $[C_{62}H_{130}O_{19}Si_6+NH_4]^+$: 1364.8160; obsd.: 1364.8179.

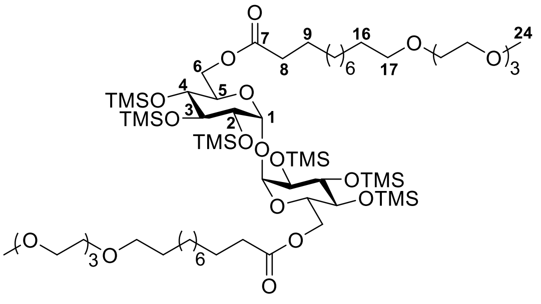

11c

6,6´-Di-*O*-(11-(2-(2-(2-methoxyethoxy)ethoxy)-ethoxy)undecanoyl)-2,2´,3,3´,4,4´-hexa-*O*-trimethylsilyl-1,1´-*α*,*α*´-D-trehalose (11c): Diol **10** (150 mg, 0.193 mmol) was subjected to the general esterification procedure with acid **9c** (245 mg, 0.702 mmol), EDCI (223 mg, 1.16 mmol) and DMAP (23.6 mg, 0.193 mmol), and the reaction stired for 36 h. Following work-up and purification via gradient silica gel flash chromatography (eluting with Pet. Ether:EtOAc, 1:0→2:3, *v/v*), the title compound **11c** was obtained as a colourless viscous oil (277 mg, 0.193 mmol, quantitative). R_f = 0.19 (Pet. Ether:EtOAc, 1:1, *v/v*); $[\alpha]_D^{21.4}$ = +58 (c = 1.0, CH_2Cl_2); ^{1}H NMR (500 MHz, $CDCl_3$) δ 4.91 (d, $J_{1,2}$ = 3.1 Hz, 2H, H-1), 4.26 (dd, $J_{6a,b}$ = 12.0 Hz, $J_{5,6a}$ = 2.3 Hz, 2H, H-6a), 4.05 (dd, $J_{6a,b}$ = 12.0 Hz, $J_{5,6b}$ = 4.4 Hz, 2H, H-6b), 3.99 (ddd, $J_{4,5}$ = 9.7 Hz, $J_{5,6b}$ = 4.4 Hz, $J_{5,6a}$ = 2.3 Hz, 2H, H-5), 3.89 (t, $J_{2,3}$ = $J_{3,4}$ = 8.9 Hz, 2H, H-3), 3.66–3.62 (m, 16H, OCH_2CH_2O), 3.58–3.53 (m, 8H, OCH_2CH_2O), 3.49–3.42 (m, 8H, H-2, H-4 and H-17), 3.37 (s, 6H, H-24), 2.38–2.28 (m, 4H, H-8), 1.63–1.53 (m, 8H, H-9 and H-16), 1.32–1.25 (m, 24H, H-10–H-15), 0.14, 0.13, 0.12 (3s, 54H, CH_3, TMS); ^{13}C NMR (125 MHz, $CDCl_3$) δ 173.9 (C-7), 94.6 (C-1), 73.6 (C-3), 72.8 (C-2), 72.0 (C-4), 71.7 (C-17), 70.84 (C-5), 72.1, 70.75, 70.72, 70.65, 70.2 (C-18–C-23), 63.4 (C-6), 59.2 (C-24), 34.3 (C-8), 29.8, 29.7, 29.60, 29.56, 29.4, 29.3, 26.2, 24.9 (C-9–C-16), 1.2, 1.0, 0.3 (TMS); IR (film): 2926, 2856, 1740, 1250, 1145, 1108, 1075, 1044, 1008, 965, 897, 871, 837, 747, 684 cm^{-1}; HRMS (ESI) *m/z* calcd. for $[C_{66}H_{138}O_{21}Si_6+NH_4]^+$: 1452.8684; obsd.: 1452.8693.

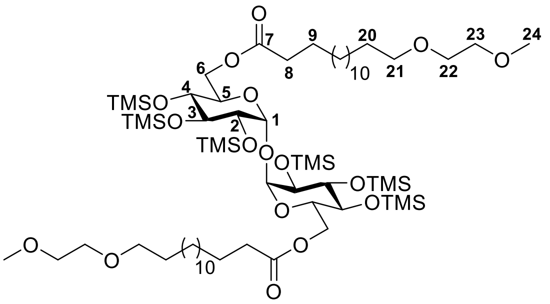

11d

6,6´-Di-*O*-(15-(2-methoxyethoxy)pentadecanoyl)-2,2´,3,3´,4,4´-hexa-*O*-trimethylsilyl-1,1´-*α*,*α*´-D-trehalose (11d): TMS-protected diol **10** (82.8 mg, 0.011 mmol) was subject to the general esterification procedure with carboxylic acid **9d** (202 mg, 0.64 mmol), DMAP (20.3 mg, 0.17 mmol), and EDCI (127 mg, 0.66 mmol). After 20 h, additional portions of DMAP (7.3 mg, 0.06 mmol) and EDCI (20.6 mg, 0.107 mmol) were added and the solution reacted for a further 24 h. Once complete (44 h), as gauged by TLC, the reaction was worked-up, and the residue purified by gradient silica gel column chromatography (Pet. Ether: EtOAc, 1:0→4:1, *v/v*) to give the title compound **11d** as a colourless viscous oil (95.9 mg, 0.070 mmol, 65%). R_f = 0.47 (Pet. Ether:EtOAc, 4:1, *v/v*); $[\alpha]_D^{22.4}$ = +60 (c = 0.1, CH_2Cl_2); ^{1}H NMR (500 MHz, $CDCl_3$) δ 4.92 (d, $J_{1,2}$ = 3.1 Hz, 2H, H-1), 4.27 (dd, $J_{6a,b}$ = 12.0 Hz, $J_{5,6a}$ = 2.3 Hz, 2H, H-6a), 4.05 (dd, $J_{6a,b}$ = 12.0 Hz, $J_{5,6b}$ = 4.4 Hz, 2H, H-6b), 4.01–3.98 (m, 2H, H-5), 3.90 (t, $J_{2,3}$ = $J_{3,4}$ = 8.9 Hz, 2H, H-3), 3.58–3.53 (m, 8H, OCH_2CH_2O), 3.49–3.42 (m, 8H, H-2, H-4 and H-21), 3.39 (s, 6H, H-24), 2.39–2.28 (m, 4H, H-8), 1.64–1.56 (m, 8H, H-9 and H-20), 1.33–1.24 (m, 40H, H-10–H-19), 0.15, 0.13, 0.13 (3s, 54H, CH_3, TMS); ^{13}C NMR (125 MHz, $CDCl_3$) δ 173.9 (C-7), 94.5 (C-1), 73.6 (C-3), 72.7 (C-2), 72.1 (C-23), 72.0 (C-4), 71.7 (C-21), 70.8 (C-5), 70.1 (C-22), 63.4 (C-6), 59.2 (C-24), 34.2 (C-8), 29.74, 29.70, 29.6, 29.4, 29.3, 26.2 (C-10–C-20), 24.9 (C-9), 1.1, 1.0, 0.3 (TMS); IR (film): 2924, 2854, 1741, 1250, 1163, 1110, 1076, 1045, 1009, 897,

871, 840, 748 cm^{-1}; HRMS (ESI) *m/z* calcd. for $[C_{66}H_{138}O_{17}Si_6+NH_4]^+$: 1388.8888; obsd.: 1388.8891.

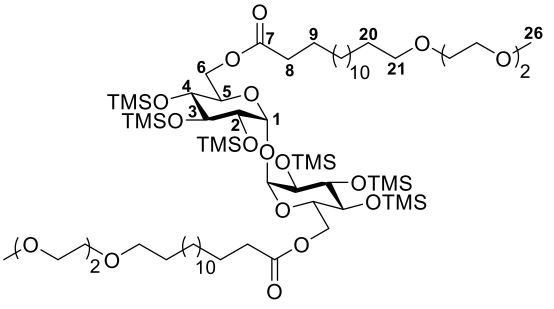

11e

6,6´-Di-*O*-(15-(2-(2-methoxyethoxy)ethoxy)-pentadecanoyl)-2,2´,3,3´,4,4´-hexa-*O*-trimethylsilyl-1,1´-α,α´-D-trehalose (11e): TMS-protected trehalose **10** (105 mg, 0.014 mmol) was subject to the general esterification procedure with glycol functionalised carboxylic acid **9e** (304 mg, 0.842 mmol), DMAP (26.9 g, 0.220 mmol), and EDCI (162 mg, 0.847 mmol). After 20 h, additional DMAP (8.55 g, 0.070 mmol) and EDCI (27.4 g, 0.143 mmol) were added, and the solution left to react for a further 24 h. Following work-up and purification by gradient silica gel flash column chromatography (Pet. Ether: EtOAc, 1:0→3:1, *v/v*), the title compound **11e** was obtained as a colourless viscous oil (174 mg, 0.118 mmol, 87%). R_f = 0.63 (Pet. Ether:EtOAc, 1:1, *v/v*); $[\alpha]_D^{22.9}$ = +60 (*c* = 0.1, CH_2Cl_2); ^{1}H NMR (500 MHz, $CDCl_3$) δ 4.92 (d, $J_{1,2}$ = 3.1 Hz, 2H, H-1), 4.27 (dd, $J_{6a,b}$ = 11.9 Hz, $J_{5,6a}$ = 2.3 Hz, 2H, H-6a), 4.05 (dd, $J_{5,6b}$ = 11.9 Hz, $J_{5,6b}$ = 4.4 Hz, 2H, H-6b), 4.01–3.98 (m, 2H, H-5), 3.90 (t, $J_{2,3}$ = $J_{3,4}$ = 8.9 Hz, 2H, H-3), 3.66–3.63 (m, 8H, OCH_2CH_2O), 3.60–3.58 (m, 4H, OCH_2CH_2O), 3.56–3.54 (m, 4H, OCH_2CH_2O), 3.50–3.42 (m, 8H, H-2, H-4 and H-21), 3.38 (s, 6H, H-26), 2.39–2.27 (m, 4H, H-8), 1.63–1.54 (m, 8H, H-20 and H-9), 1.33–1.24 (m, 40H, H-10–H-19), 0.15, 0.13, 0.13 (3s, 54H, CH_3, TMS); ^{13}C NMR (125 MHz, $CDCl_3$) δ 173.9 (C-7), 94.6 (C-1), 73.6 (C-3), 72.8 (C-2), 72.1 (C-25), 72.0 (C-4), 71.7 (C-21), 70.84 (C-5), 70.83, 70.7 (C-23 and C-24), 70.2 (C-22), 63.4 (C-6), 59.2 (C-26), 34.3 (C-8), 29.8, 29.7, 29.5, 29.3, 26.2 (C-10–C-20), 24.9 (C-9), 1.2, 1.0, 0.3 (TMS); IR (film): 2925, 2855, 1741, 1251, 1164, 1146, 1111, 1077, 1045, 1010, 898, 872, 843, 749 cm^{-1}; HRMS (ESI) *m/z* calcd. for $[C_{70}H_{146}O_{19}Si_6+NH_4]^+$: 1476.9412; obsd.: 1476.9411.

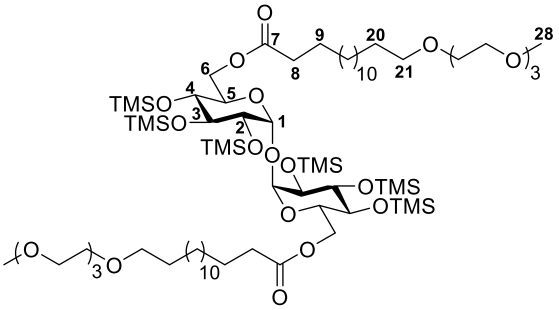

11f

6,6´-Di-*O*-(15-(2-(2-(2-methoxyethoxy)-ethoxy)ethoxy)pentadecanoyl)-2,2´,3,3´,4,4´-hexa-*O*-trimethylsilyl-1,1´-α,α´-D-trehalose (11f): TMS-protected diol **10** (35.2 mg, 0.045 mmol) was subjected to the general esterification procedure with carboxylic acid **9f** (55.1 mg, 0.14 mmol), DMAP (5.5 mg, 0.045 mmol) and EDCI (52.2 mg, 0.272 mmol), and the reaction mixture stirred for 72 h Following work-up and purification by gradient silica gel flash chromatography (eluting with Pet. Ether:EtOAc, 1:0→3:1, *v/v*), the title compound **11f** was obtained as a clear viscous oil (49.5 mg, 0.032 mmol, 71%). R_f = 0.38 (Pet. Ether:EtOAc, 2:3, *v/v*); $[\alpha]_D^{22.9}$ = +40 (*c* = 0.1, CH_2Cl_2); ^{1}H NMR (500 MHz, $CDCl_3$) δ 4.92 (d, $J_{1,2}$ = 3.1 Hz, 2H, H-1), 4.27 (dd, $J_{6a,b}$ = 12.0 Hz, $J_{5,6a}$ = 2.3 Hz, 2H, H-6a), 4.05 (dd, $J_{6a,b}$ = 12.0 Hz, $J_{5,6b}$ = 4.4 Hz, 2H, H-6b), 4.00 (ddd, $J_{4,5}$ = 9.6 Hz, $J_{5,6b}$ = 4.4 Hz, $J_{5,6a}$ = 2.3 Hz, 2H, H-5), 3.90 (t, $J_{2,3}$ = $J_{3,4}$ = 8.9 Hz, 2H, H-3), 3.67–3.63 (m, 16H, OCH_2CH_2O), 3.59–3.54 (m, 8H, OCH_2CH_2O), 3.50–3.42 (m, 8H, H-2, H-4 and H-21), 3.38 (s, 6H, H-28), 2.39–2.28 (m, 4H, H-8), 1.63–1.54 (m, 8H, H-9 and H-20), 1.32–1.24 (m, 40H, H-10–H-19), 0.15, 0.13, 0.13 (3s, 54H, CH_3, TMS); ^{13}C NMR (125 MHz, $CDCl_3$) δ 173.9 (C-7), 94.6 (C-1), 73.6 (C-3), 72.8 (C-2), 72.1 (C-27), 72.0 (C-4), 71.7 (C-21), 70.9 (C-5), 70.78, 70.74, 70.67 (C-23–C-26), 70.2 (C-22), 63.4 (C-6), 59.2 (C-28), 34.3 (C-8), 29.83, 29.79, 29.66, 29.5, 29.3, 26.3 (C-10–C-20), 24.9 (C-9),

1.2, 1.0, 0.3 (TMS); IR (film): 2924, 2854, 1741, 1250, 1144, 1109, 1076, 1045, 1008, 897, 871, 838, 748 cm^{-1}; HRMS (ESI) *m/z* calcd. for $[C_{74}H_{154}O_{21}Si_6+NH_4]^+$: 1564.9937; obsd.: 1564.9911.

General procedure for desilylation: To a solution of TMS-protected trehalose diester **11a-f** in 1:1 (*v/v*) CH_2Cl_2:MeOH (20 mL/mmol), was added Dowex-H$^+$ until the pH of the reaction mixture was ~2-3. The mixture was then stirred at room temperature for 1.5-2.5 h until the reaction was deemed complete, as gauged by TLC. The reaction mixture was then filtered, washed with 1:1 (*v/v*) CH_2Cl_2:MeOH followed by 100% MeOH, and concentrated under reduced pressure.

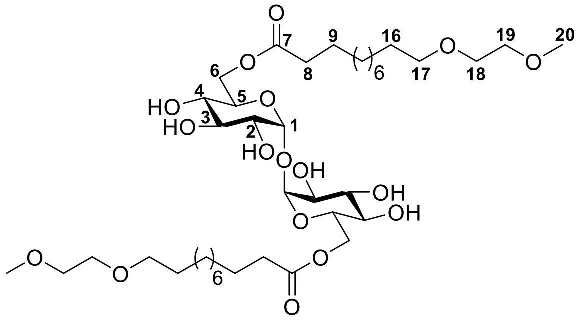

4a

6,6′-Di-*O*-(11-(2-methoxyethoxy)undecanoyl)-1,1′-α,α′-D-trehalose (4a): By subjecting protected trehalose diester **11a** (40.0 mg, 0.032 mmol) to the general procedure for desilylation followed by purification of the resulting residue via gradient silica gel flash column chromatography (eluted with CH_2Cl_2:MeOH, 1:0→9:1, *v/v*), the title compound **4a** was obtained as a white thin film (19.9 mg, 0.024 mmol, 76%). R_f = 0.39 (CH_2Cl_2:MeOH, 17:3, *v/v*); $[\alpha]_D^{22.5}$ = +60 (*c* = 0.1, CH_3OH); ^{1}H NMR (500 MHz, CD_3OD) δ 5.04 (d, $J_{1,2}$ = 3.7 Hz, 2H, H-1), 4.35 (dd, $J_{6a,b}$ = 11.8 Hz, $J_{5,6a}$ = 2.2 Hz, 2H, H-6a), 4.20 (dd, $J_{6a,b}$ = 11.8 Hz, $J_{5,6b}$ = 5.3 Hz, 2H, H-6b), 4.01 (ddd, $J_{4,5}$ = 10.1 Hz, $J_{5,6b}$ = 5.3 Hz, $J_{5,6a}$ = 2.2 Hz, 2H, H-5), 3.78 (dd, $J_{2,3/3,4}$ = 9.7 Hz, $J_{2,3/3,4}$ = 8.9 Hz, 2H, H-3), 3.57–3.52 (m, 8H, OCH_2CH_2O), 3.48–3.45 (m, 6H, H-2 and H-17), 3.36 (s, 6H, H-20), 3.35–3.32 (m, 2H, H-4), 2.34 (t, $J_{8,9}$ = 7.4 Hz, 4H, H-8), 1.66–1.52 (m, 8H, H-9 and H-16), 1.37–1.29 (m, 24H, H-10–H-15); ^{13}C NMR (125 MHz, CD_3OD) δ 175.5 (C-7), 95.2 (C-1), 74.6 (C-3), 73.2 (C-2), 72.4 (C-17), 71.9 (C-4), 71.5 (C-5), 72.97, 70.9 (C-18 and C-19), 64.4 (C-6), 59.1 (C-20), 35.1 (C-8), 30.7, 30.6, 30.4, 30.2, 27.2 (C-10–C-16), 26.1 (C-9); IR (film): 3330 (broad), 2926, 2855, 1738, 1455, 1356, 1284, 1250, 1150, 1110, 1082, 1055, 987, 938 cm^{-1}; HRMS (ESI) *m/z* calcd. for $[C_{40}H_{74}O_{17}+NH_4]^+$: 844.5264; obsd.: 844.5265.

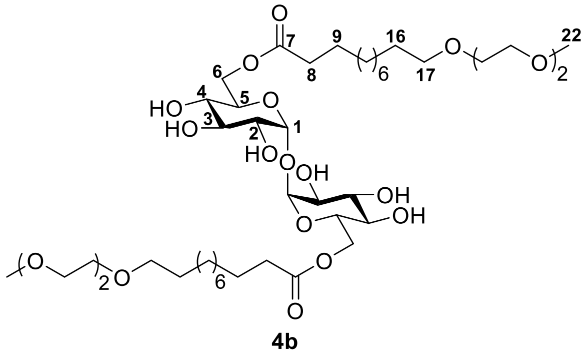

4b

6,6′-Di-*O*-(11-(2-(2-methoxyethoxy)-ethoxy)undecanoyl)-1,1′-α,α′-D-trehalose (4b): Protected trehalose diester **11b** (20.9 mg, 0.016 mmol) was subjected to the general procedure for desilylation. The resulting residue was then purified via gradient silica gel flash column chromatography (eluting with CH_2Cl_2:MeOH, 1:0→22:3, *v/v*) to give the title compound **4b** as a white thin film (12.3 mg, 0.013 mmol, 87%). R_f = 0.52 (CH_2Cl_2:MeOH, 4:1, *v/v*); $[\alpha]_D^{23.4}$ = +40 (*c* = 0.1, CH_3OH);^{1}H NMR (500 MHz, CD_3OD) δ 5.04 (d, $J_{1,2}$ = 3.9 Hz, 2H, H-1), 4.35 (dd, $J_{6a,b}$ = 11.9 Hz, $J_{5,6a}$ = 2.2 Hz, 2H, H-6a), 4.20 (dd, $J_{6a,b}$ = 11.9 Hz, $J_{5,6b}$ = 5.3 Hz, 2H, H-6b), 4.02 (ddd, $J_{4,5}$ = 10.1 Hz, $J_{5,6b}$ = 5.3 Hz, $J_{5,6a}$ = 2.2 Hz, 2H, H-5), 3.78 (t, $J_{2,3}$ = $J_{3,4}$ = 9.3 Hz, 2H, H-3), 3.64–3.61 (m, 8H, OCH_2CH_2O), 3.58–3.56 (m, 4H, OCH_2CH_2O), 3.55–3.53 (m, 4H, OCH_2CH_2O), 3.48–3.45 (m, 6H, H-2 and H-17), 3.36 (s, 6H, H-22), 3.35–3.31 (m, 2H, H-4), 2.34 (t, $J_{8,9}$ = 7.4 Hz, 4H, H-8), 1.65–1.54 (m, 8H, H-9 and H-16), 1.37–1.32 (m, 24H, H-10–H-15); ^{13}C NMR (125 MHz,

CD_3OD) δ 175.4 (C-7), 95.2 (C-1), 74.6 (C-3), 73.2 (C-2), 73.0 (C-21), 72.4 (C-17), 71.9 (C-4), 71.5 (C-5), 71.6, 71.4 (C-19 and C-20), 71.1 (C-18), 64.4 (C-6), 59.1 (C-22), 35.1, 30.73, 30.69, 30.56, 30.4, 30.2, 27.2 (C-10–C-16), 26.1 (C-9); IR (film): 3314 (broad), 2927, 2856, 1737, 1455, 1351, 1290, 1250, 1115, 1081, 1054, 1030, 988, 937 cm^{-1}; HRMS (ESI) *m/z* calcd. for $[C_{44}H_{82}O_{19}+NH_4]^+$: 932.5789; obsd.: 932.5789.

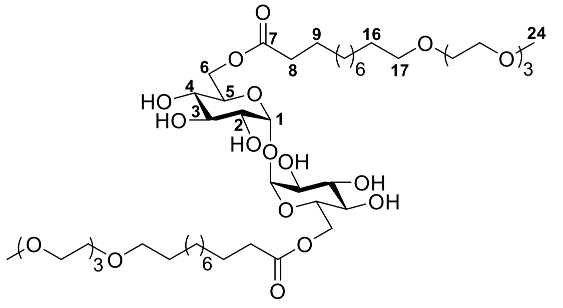

4c

6,6′-Di-*O*-(11-(2-(2-(2-methoxyethoxy)-ethoxy)ethoxy)undecanoyl)-1,1′-α,α′-D-trehalose (4c): TMS protected diester **11c** (46.6 mg, 0.032 mmol) was subjected to the general procedure for desilylation, followed by gradient silica gel flash column chromatography (eluted with CH_2Cl_2:MeOH, 1:0→4:1, *v/v*) to give the title compound **4c** as a white solid (28.5 mg, 0.028 mmol, 88%). R_f = 0.55 (CH_2Cl_2:MeOH, 4:1, *v/v*); $[\alpha]_D^{21.7}$ = +50 (*c* = 0.08, CH_3OH); ^1H NMR (500 MHz, CD_3OD) δ 5.04 (d, $J_{1,2}$ = 3.7 Hz, 2H, H-1), 4.35 (dd, $J_{6a,b}$ = 11.9 Hz, $J_{5,6a}$ = 2.2 Hz, 2H, H-6a), 4.20 (dd, $J_{6a,b}$ = 11.9 Hz, $J_{5,6b}$ = 5.3 Hz, 2H, H-6b), 4.01 (ddd, $J_{4,5}$ = 10.2 Hz, $J_{5,6b}$ = 5.3, $J_{5,6a}$ = 2.2 Hz, 2H, H-5), 3.78 (t, $J_{2,3}$ = $J_{3,4}$ = 9.3 Hz, 2H, H-3), 3.64–3.61 (m, 16H, OCH_2CH_2O), 3.59–3.53 (m, 8H, OCH_2CH_2O), 3.48–3.46 (m, 6H, H-2 and H-17), 3.36 (s, 6H, H-24), 3.34 (m, 2H, H-4), 2.34 (t, $J_{8,9}$ = 7.4 Hz, 4H, H-8), 1.63–1.56 (m, 8H, H-9 and H-16), 1.37–1.32 (m, 24H, H-10–H-15); ^{13}C NMR (125 MHz, CD_3OD) δ 175.5 (C-7), 95.2 (C-1), 74.5 (C-3), 73.2 (C-2), 72.4 (C-17), 71.9 (C-4), 71.5 (C-5), 73.0, 71.6, 71.4, 71.1 (C-18–C-23), 64.4 (C-6), 59.1 (C-24), 35.1 (C-8), 30.73, 30.69, 30.57, 30.55, 30.41, 30.2, 27.2 (C-10–C-16), 26.1 (C-9); IR (film): 3303 (broad), 2924, 2855, 1734, 1654, 1455, 1351, 1251, 1197, 1106, 1049, 1028, 982, 851, 805, 723, 578, 530 cm^{-1}; HRMS (ESI) *m/z* calcd. for $[C_{48}H_{90}O_{21}+NH_4]^+$: 1020.6313; obsd.: 1020.6314.

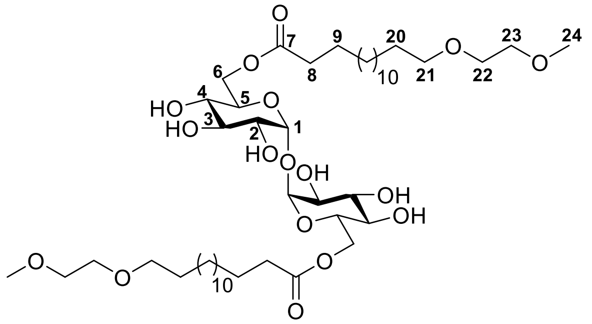

4d

6,6′-Di-*O*-(15-(2-methoxyethoxy)pentadecanoyl)-1,1′-α,α′-D-trehalose (4d): By subjecting protected trehalose diester **11d** (12.6 mg, 0.0092 mmol) to the general procedure for desilylation, followed by purification of the resulting residue using gradient silica gel flash column chromatography (eluting with CH_2Cl_2:MeOH, 1:0→4:1, *v/v*), the title compound **4d** was obtained as a white thin film (8.1 mg, 0.0086 mmol, 94%). R_f = 0.55 (CH_2Cl_2:MeOH, 4:1, *v/v*); $[\alpha]_D^{22.0}$ = +75 (*c* = 0.08, CH_3OH); ^1H NMR (600 MHz, CD_3OD) δ 5.05 (d, $J_{1,2}$ = 3.9 Hz, 2H, H-1), 4.35 (dd, $J_{6a,b}$ = 11.9 Hz, $J_{5,6a}$ = 2.2 Hz, 2H, H-6a), 4.20 (dd, $J_{6a,b}$ = 11.9 Hz, $J_{5,6b}$ = 5.3 Hz, 2H, H-6b), 4.01 (ddd, $J_{4,5}$ = 10.0 Hz, $J_{5,6b}$ = 5.3 Hz, $J_{5,6a}$ = 2.2 Hz, 2H, H-5), 3.78 (t, $J_{2,3}$ = $J_{3,4}$ = 9.3 Hz, 2H, H-3), 3.57–3.52 (m, 8H, OCH_2CH_2O), 3.48–3.45 (m, 6H, H-2 and H-21), 3.36 (s, 6H, H-24), 3.34–3.33 (m, 2H, H-4), 2.34 (t, $J_{8,9}$ = 7.4 Hz, 4H, H-8), 1.65–1.54 (m, 8H, H-9 and H-20), 1.37–1.30 (m, 40H, H-10–H-19); ^{13}C NMR (150 MHz, CD_3OD) δ 175.5 (C-7), 95.2 (C-1), 74.6 (C-3), 73.2 (C-2), 73.0 (C-23), 72.4 (C-21), 71.9 (C-4), 71.5 (C-5), 70.9 (C-22), 64.4 (C-6), 59.1 (C-24), 35.1 (C-8), 30.79, 30.75, 30.70, 30.6, 30.4, 30.2, 27.2 (C-10–C-20), 26.1 (C-9); IR (film): 3352 (broad), 2918, 2851, 1736, 1612, 1467, 1412, 1103, 1073, 1022, 989 cm^{-1}; HRMS (ESI) *m/z* calcd. for $[C_{48}H_{90}O_{17}+NH_4]^+$: 956.6516; obsd.: 956.6511.

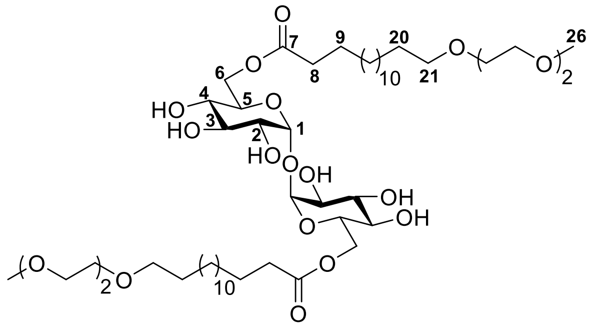

4e

6,6´-Di-*O*-(15-(2-(2-methoxyethoxy)-ethoxy)pentadecanoyl)-1,1´-α,α´-D-trehalose (4e): TMS-trehalose diester **11e** (5.9 mg, 0.0039 mmol) was subjected to the general desilylation procedure. Following completion of the reaction and work-up, the resulting residue was purified by silica gel flash chromatography (CH_2Cl_2:MeOH, 1:0→17:3, *v/v*) to give trehalose glycolipid **4e** as a white thin film (3.9 mg, 0.0038 mmol, 95%). R_f = 0.35 (CH_2Cl_2:MeOH, 17:3, *v/v*); $[\alpha]_D^{23.7}$ = +54 (*c* = 1, CH_3OH); ^{1}H NMR (600 MHz, CD_3OD) δ 5.04 (d, $J_{1,2}$ = 3.8 Hz, 2H, H-1), 4.35 (dd, $J_{6a,b}$ = 11.8 Hz, $J_{5,6a}$ = 2.0 Hz, 2H, H-6a), 4.20 (dd, $J_{6a,b}$ = 11.8 Hz, $J_{5,6b}$ = 5.3 Hz, 2H, H-6b), 4.01 (ddd, $J_{4,5}$ = 10.3 Hz, $J_{5,6b}$ = 5.3 Hz, $J_{5,6a}$ = 2.0 Hz, 2H, H-5), 3.77 (t, $J_{2,3}$ = $J_{3,4}$ = 9.3 Hz, 2H, H-3), 3.64–3.61 (m, 8H, OCH_2CH_2O), 3.58–3.56 (m, 4H, OCH_2CH_2O), 3.55–3.53 (m, 4H, OCH_2CH_2O), 3.48–3.45 (m, 6H, H-2 and H-21), 3.36 (s, 6H, H-26), 3.35–3.33 (m, 2H, H-4), 2.34 (t, $J_{8,9}$ = 7.4 Hz, 4H, H-8), 1.63–1.54 (m, 8H, H-9 and H-20), 1.37–1.27 (m, 40H, H-10–H-19); ^{13}C NMR (150 MHz, CD_3OD) δ 175.4 (C-7), 95.2 (C-1), 74.6 (C-3), 73.2 (C-2), 73.0 (C-25), 72.4 (C-21), 71.9 (C-4), 71.5 (C-5), 71.6, 71.4 (C-23–C-24), 71.2 (C-22), 64.4 (C-6), 59.1 (C-26), 35.1 (C-8), 30.79, 30.75, 30.6, 30.4, 30.2, 27.2 (C-10–C-20), 26.1 (C-9); IR (film): 3340 (broad), 2923, 2854, 1738, 1454, 1352, 1249, 1111, 1082, 1057, 987, 939 cm^{-1}; HRMS (ESI) *m/z* calcd. for $[C_{52}H_{98}O_{19}+NH_4]^+$: 1044.7041; obsd.: 1044.7048.

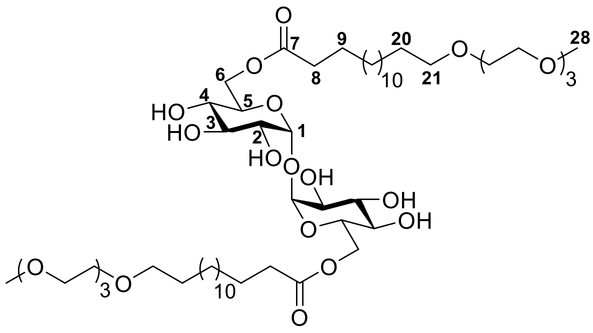

4f

6,6´-Di-*O*-(15-(2-(2-(2-methoxyethoxy)-ethoxy)ethoxy)pentadecanoyl)-1,1´-α,α´-D-trehalose (4f): By subjecting diester **11f** (29.3 mg, 0.019 mmol) to the general procedure for desilylation and purification via gradient silica gel flash column chromatography (eluted with CH_2Cl_2:MeOH, 1:0→4:1, *v/v*), the title compound **4f** was obtained as a white thin film (17.5 mg, 0.016 mmol, 83%). R_f = 0.45 (CH_2Cl_2:MeOH, 17:3, *v/v*); $[\alpha]_D^{22.6}$ = +60 (*c* = 0.1, CH_3OH); ^{1}H NMR (500 MHz, CD_3OD) δ 5.05 (d, $J_{1,2}$ = 3.8 Hz, 2H, H-1), 4.60 (bs, 1H, OH), 4.36 (dd, $J_{6a,b}$ = 11.9 Hz, $J_{5,6a}$ = 2.2 Hz, 2H, H-6a), 4.20 (dd, $J_{6a,b}$ = 11.9 Hz, $J_{5,6b}$ = 5.4 Hz, 2H, H-6b), 4.01 (ddd, $J_{4,5}$ = 10.1 Hz, $J_{5,6b}$ = 5.4 Hz, $J_{5,6a}$ = 2.2 Hz, 2H, H-5), 3.78 (t, $J_{2,3}$ = $J_{3,4}$ = 9.3 Hz, 2H, H-3), 3.64–3.61 (m, 16H, OCH_2CH_2O), 3.59–3.53 (m, 8H, OCH_2CH_2O), 3.48–3.46 (m, 6H, H-2 and H-21), 3.36 (s, 6H, H-28), 3.35–3.31 (m, 2H, H-4), 2.34 (t, $J_{8,9}$ = 7.4 Hz, 4H, H-8), 1.63–1.54 (m, 8H, H-9 and H-20), 1.37–1.30 (m, 40H, H-10–H-19); ^{13}C NMR (150 MHz, CD_3OD) δ 175.5 (C-7), 95.2 (C-1), 74.5 (C-3), 73.2 (C-2), 73.0 (C-27), 72.4 (C-21), 71.9 (C-4), 71.47 (C-5), 71.6, 71.54, 71.36 (C-23–C-26), 71.2 (C-22), 64.4 (C-6), 59.1 (C-28), 35.1 (C-8), 30.81, 30.79, 30.76, 30.74, 30.6, 30.5, 30.2, 27.2 (C-10–C-20), 26.1 (C-9); IR (film): 3326 (broad), 2923, 2853, 1737, 1458, 1351, 1250, 1110, 1081, 1054, 988 cm^{-1}; HRMS (ESI) *m/z* calcd. for $[C_{56}H_{106}O_{21}+NH_4]^+$: 1132.7565; obsd.: 1132.7561.

2 ^{1}H and ^{13}C NMR data

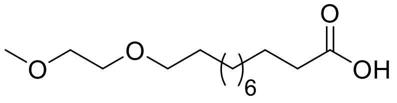

9a

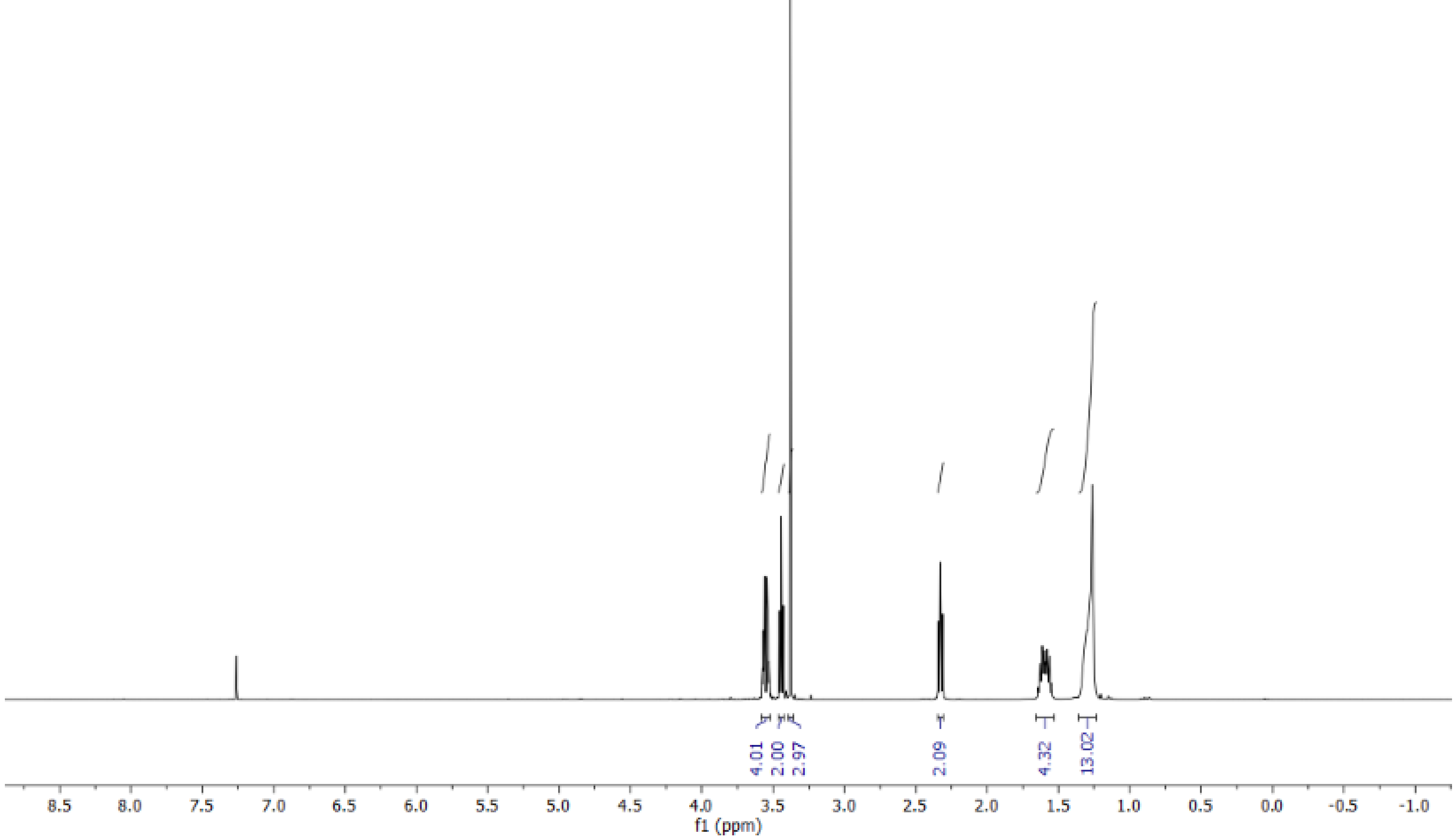

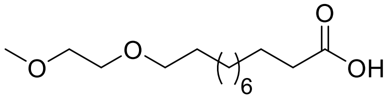

9a

^{13}C NMR (125 MHz, $CDCl_3$)

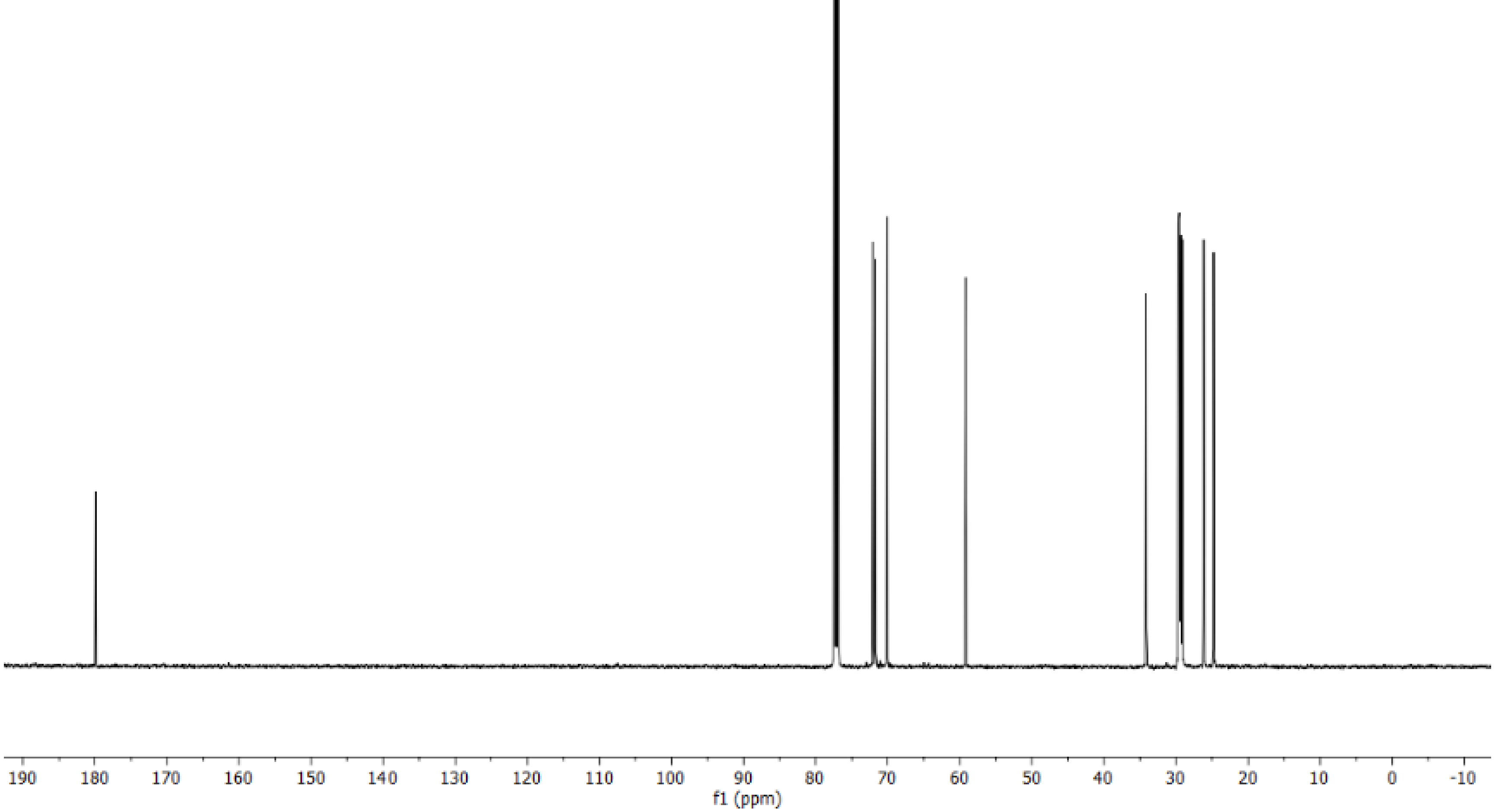

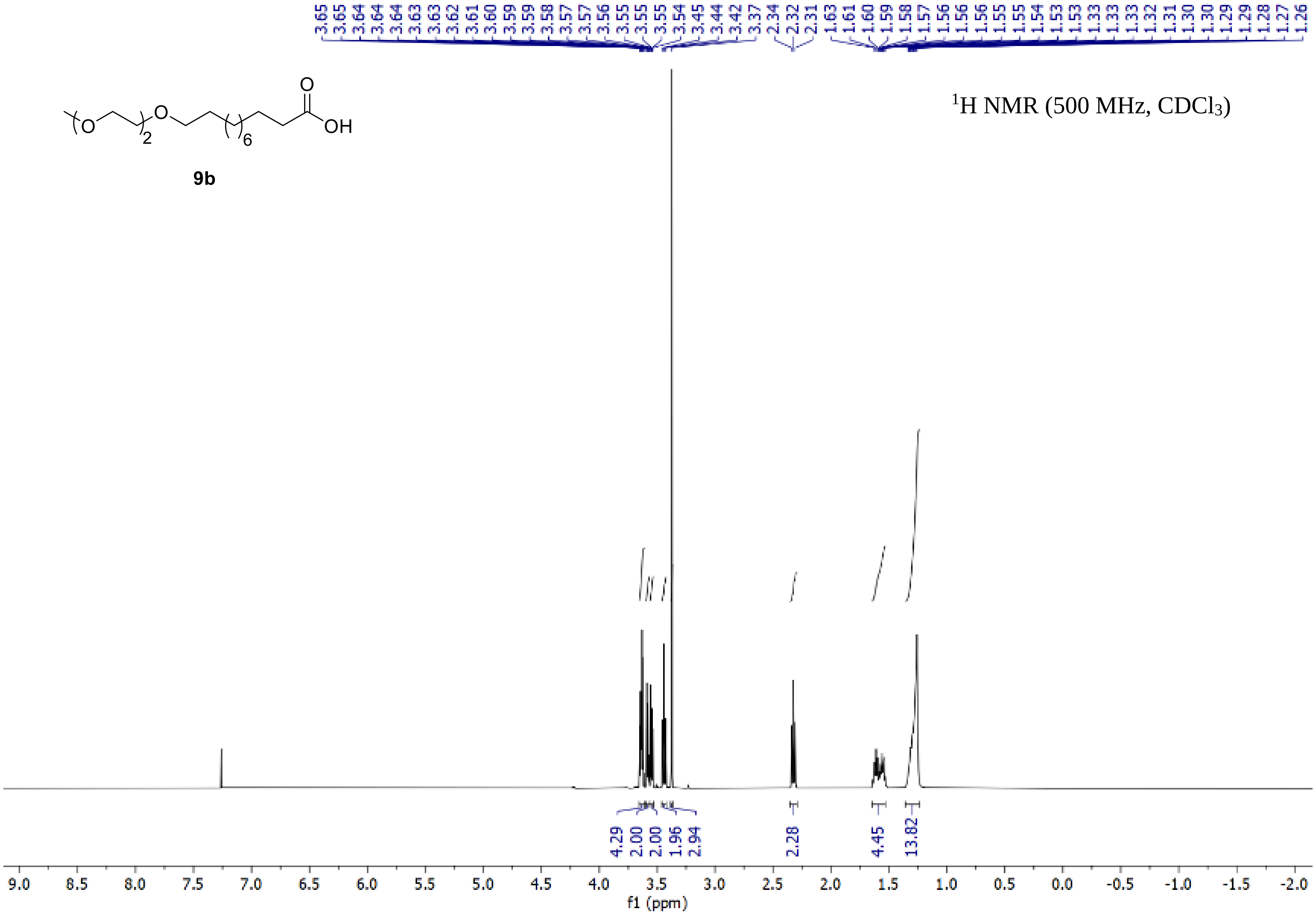

9b

^{1}H NMR (500 MHz, $CDCl_3$)

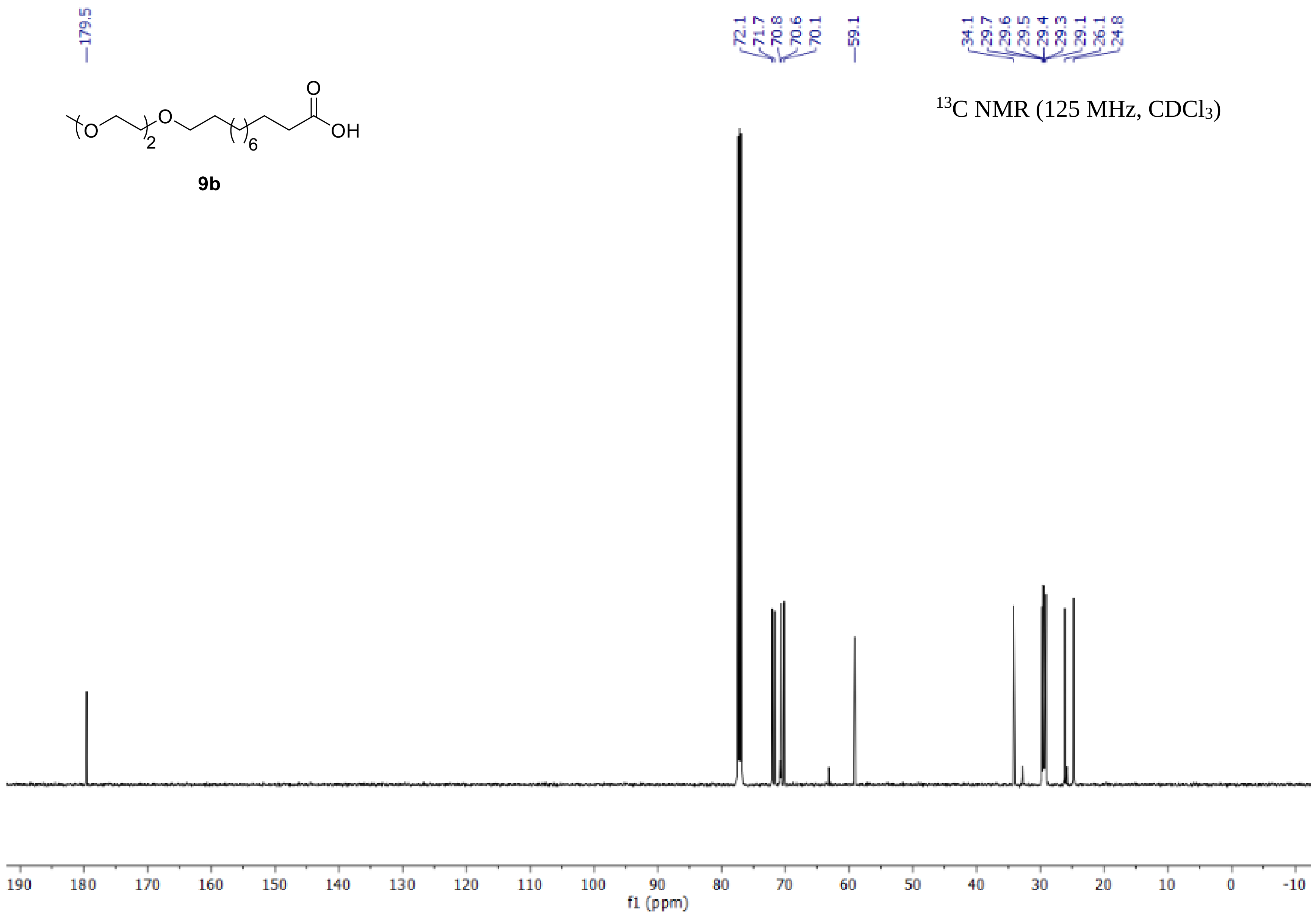

179.5
72.1
71.7
70.8
70.6
70.1
59.1
34.1
29.7
29.6
29.5
29.4
29.3
29.1
26.1
24.8
O
O
OH
O
2
6
9b
13C NMR (125 MHz, CDCl3)
190
180
170
160
150
140
130
120
110
100
90
80
70
60
50
40
30
20
10
0
-10
f1 (ppm)

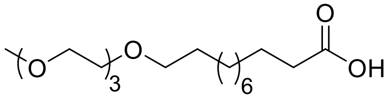

9c

^{1}H NMR (500 MHz, $CDCl_3$)

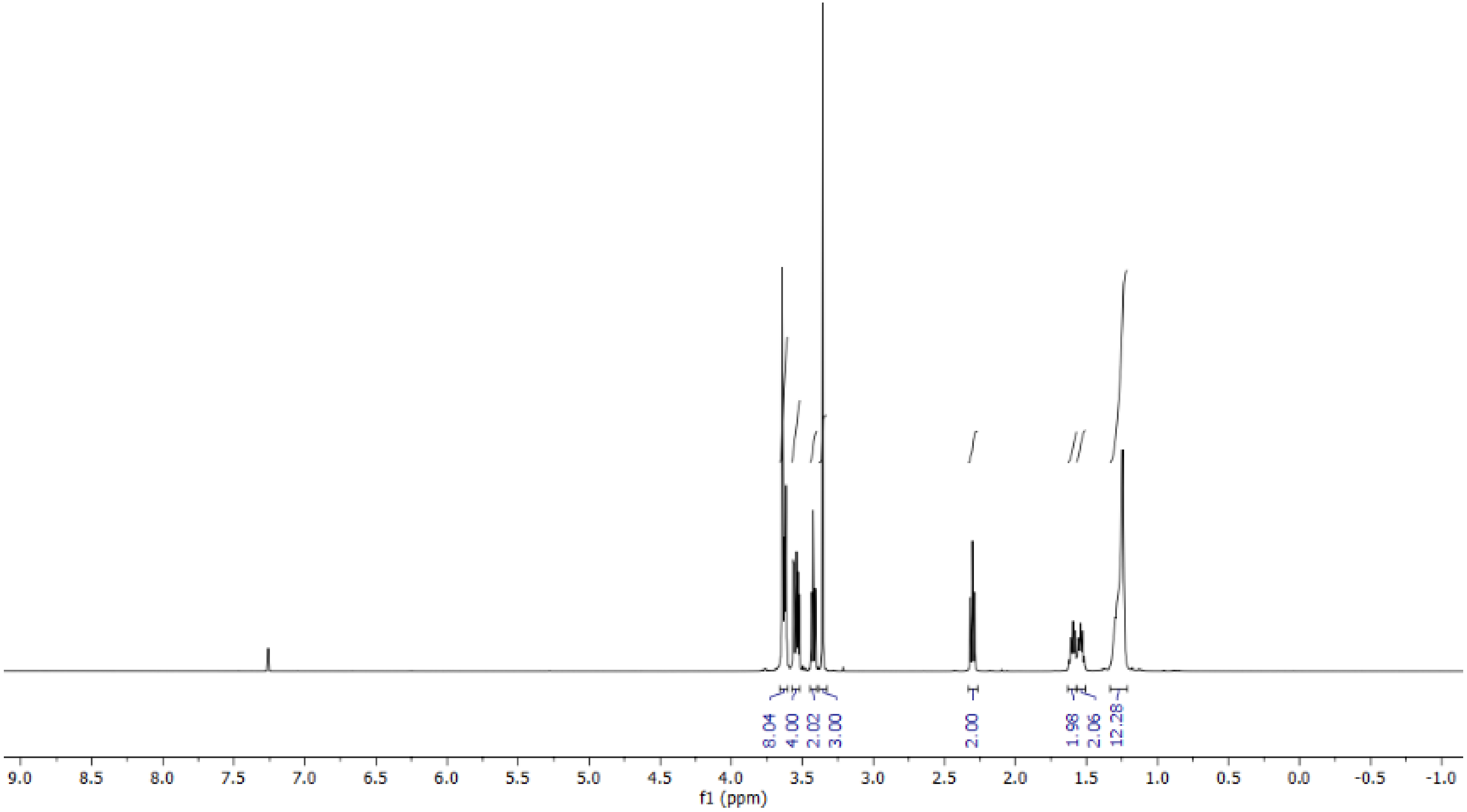

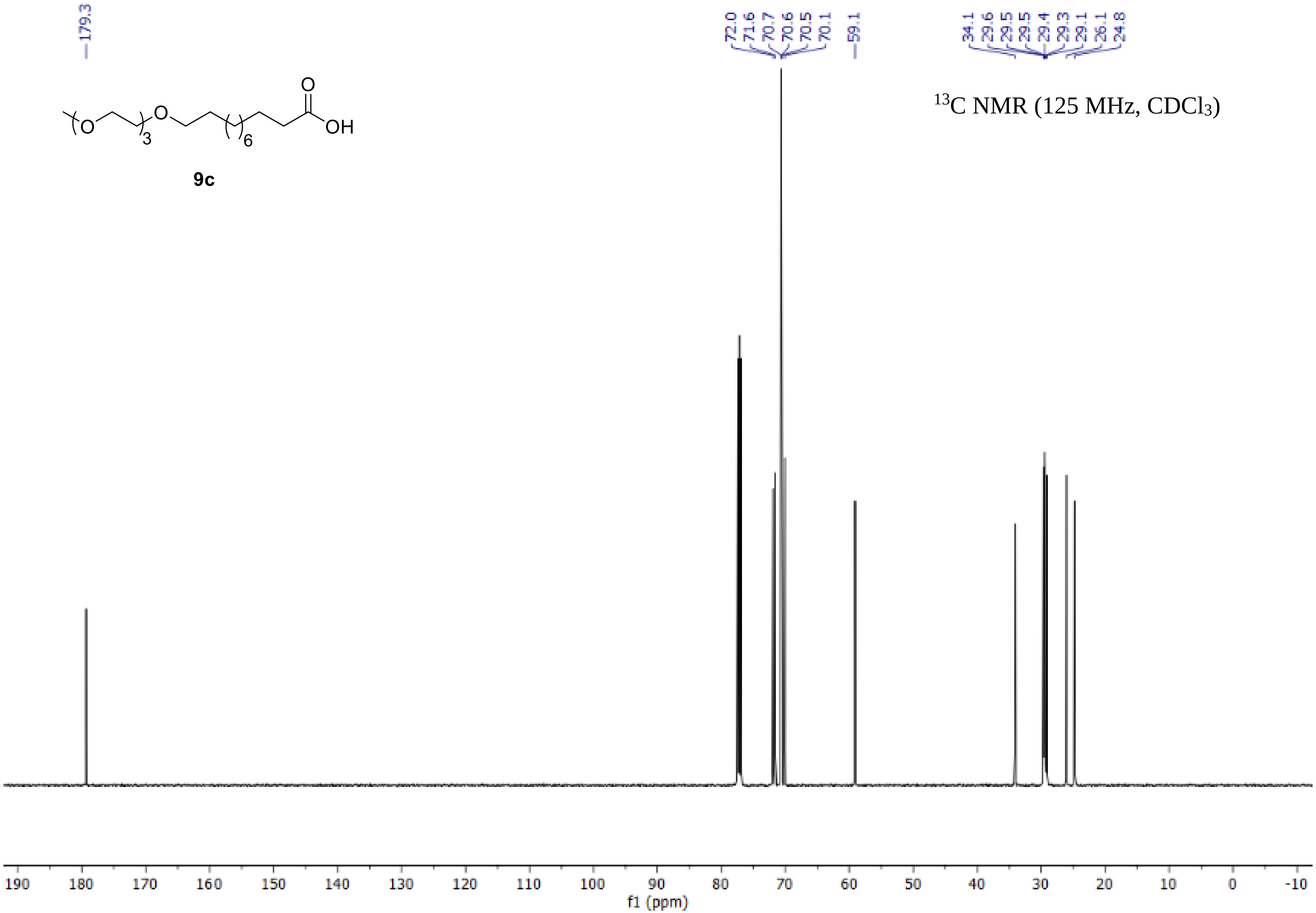

179.3
72.0
71.6
70.7
70.6
70.5
70.1
59.1
34.1
29.6
29.5
29.5
29.4
29.3
29.1
26.1
24.8
O
O
3
O
6
O
OH
9c
^{13}C NMR (125 MHz, $CDCl_3$)
190
180
170
160
150
140
130
120
110
100
90
80
70
60
50
40
30
20
10
0
-10
f1 (ppm)

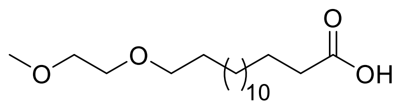

9d

¹H NMR (500 MHz, $CDCl_3$)

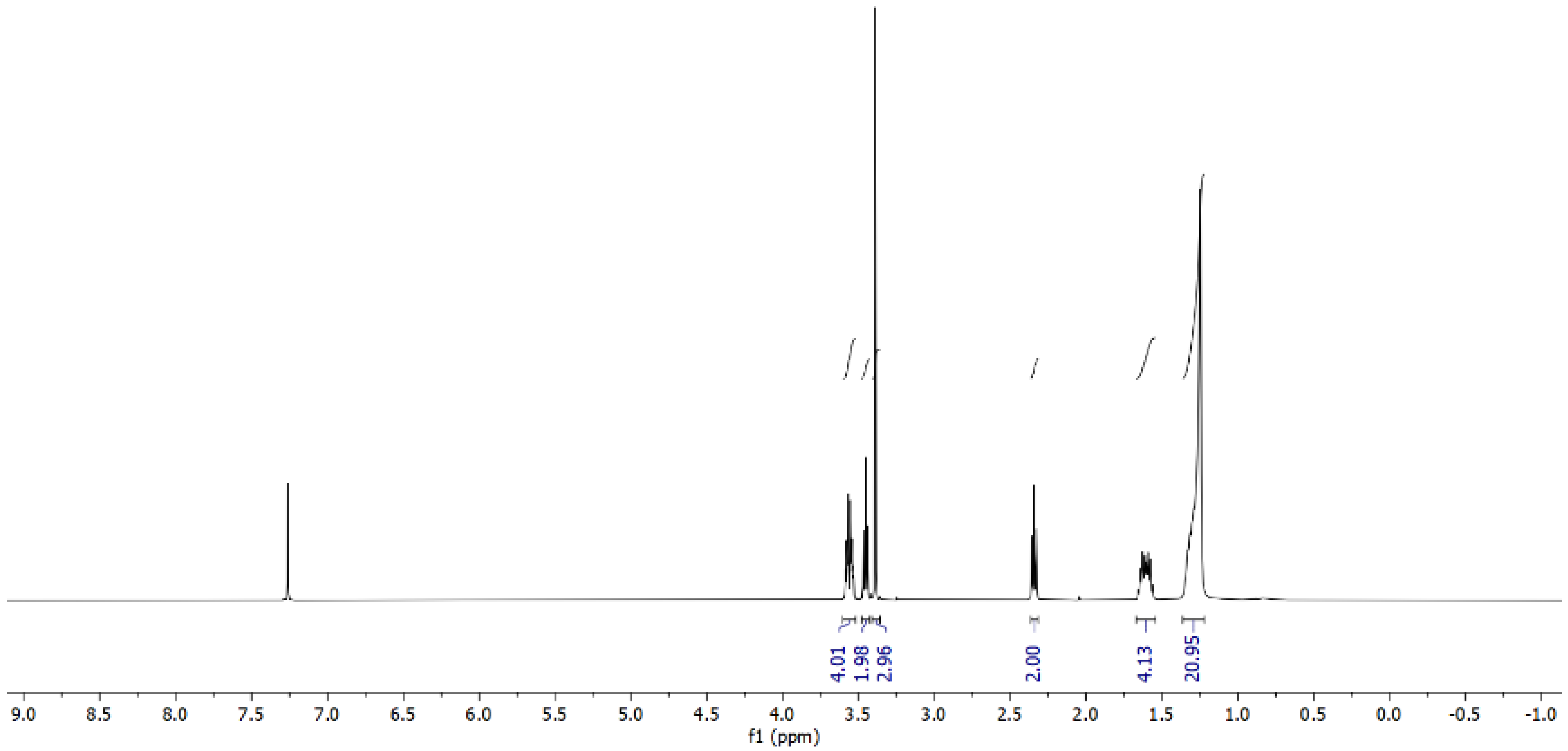

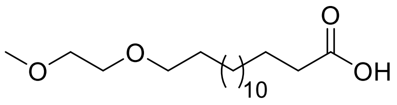

9d

^{13}C NMR (125 MHz, $CDCl_3$)

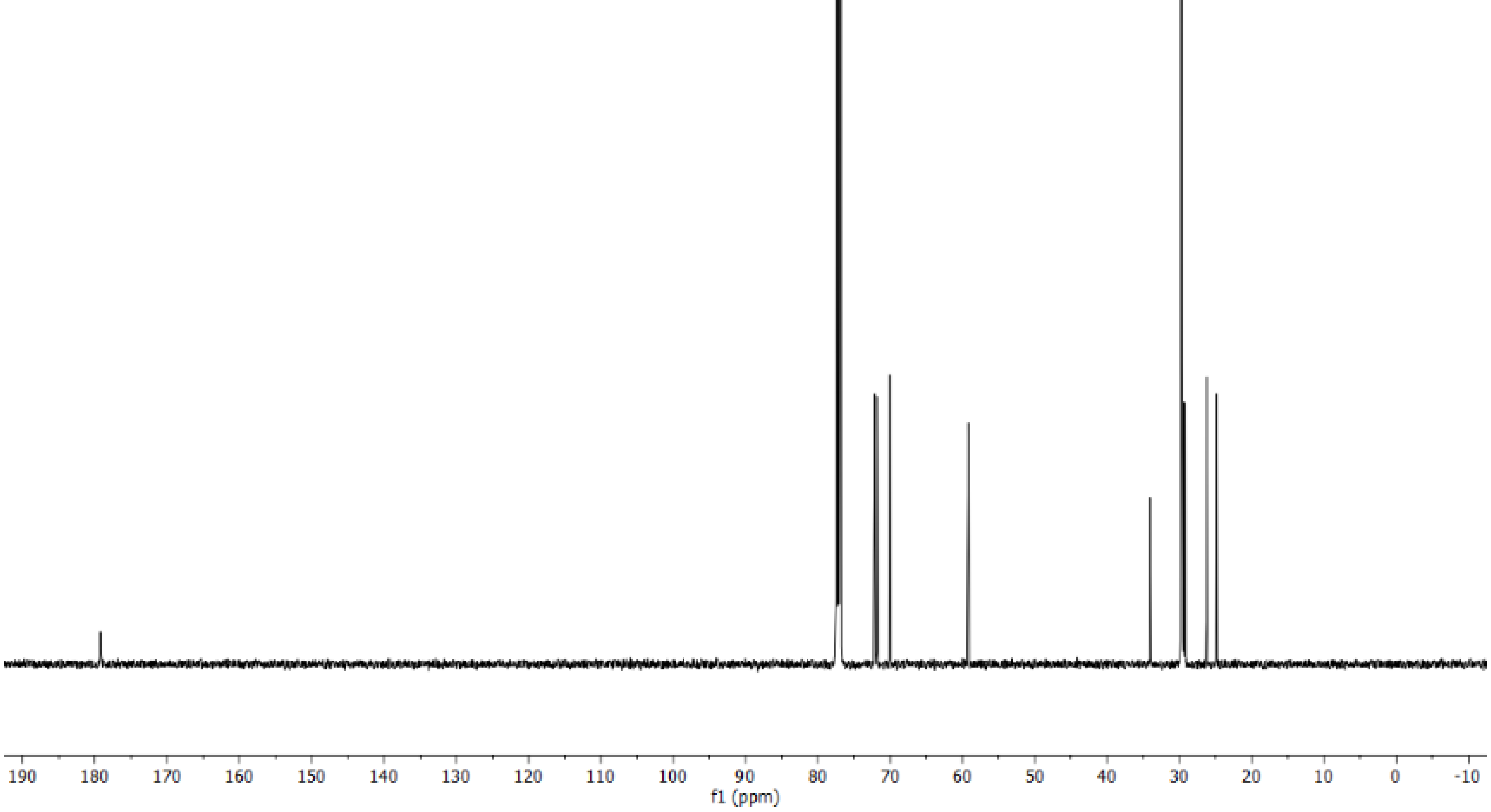

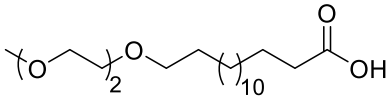

9e

^{1}H NMR (500 MHz, $CDCl_3$)

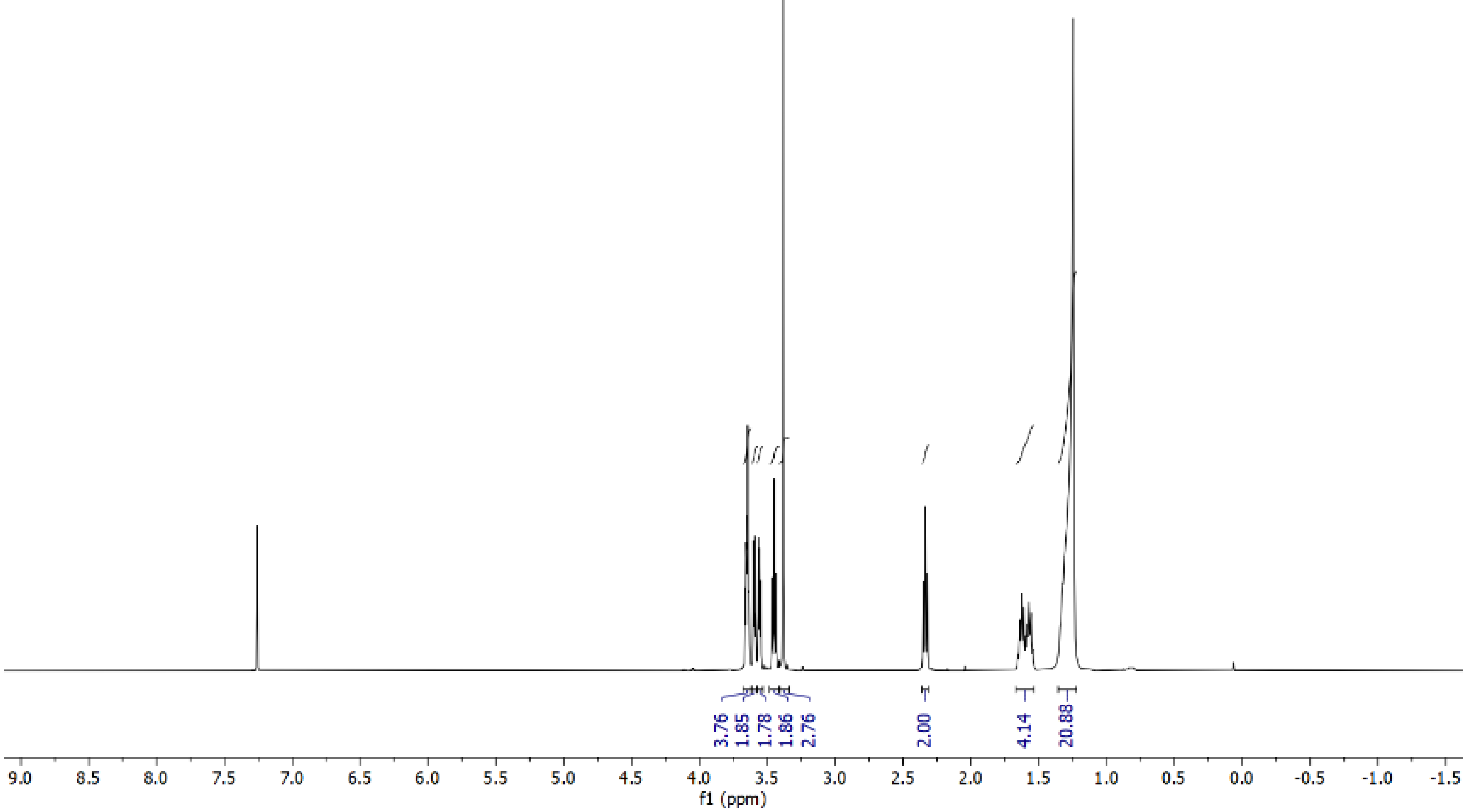

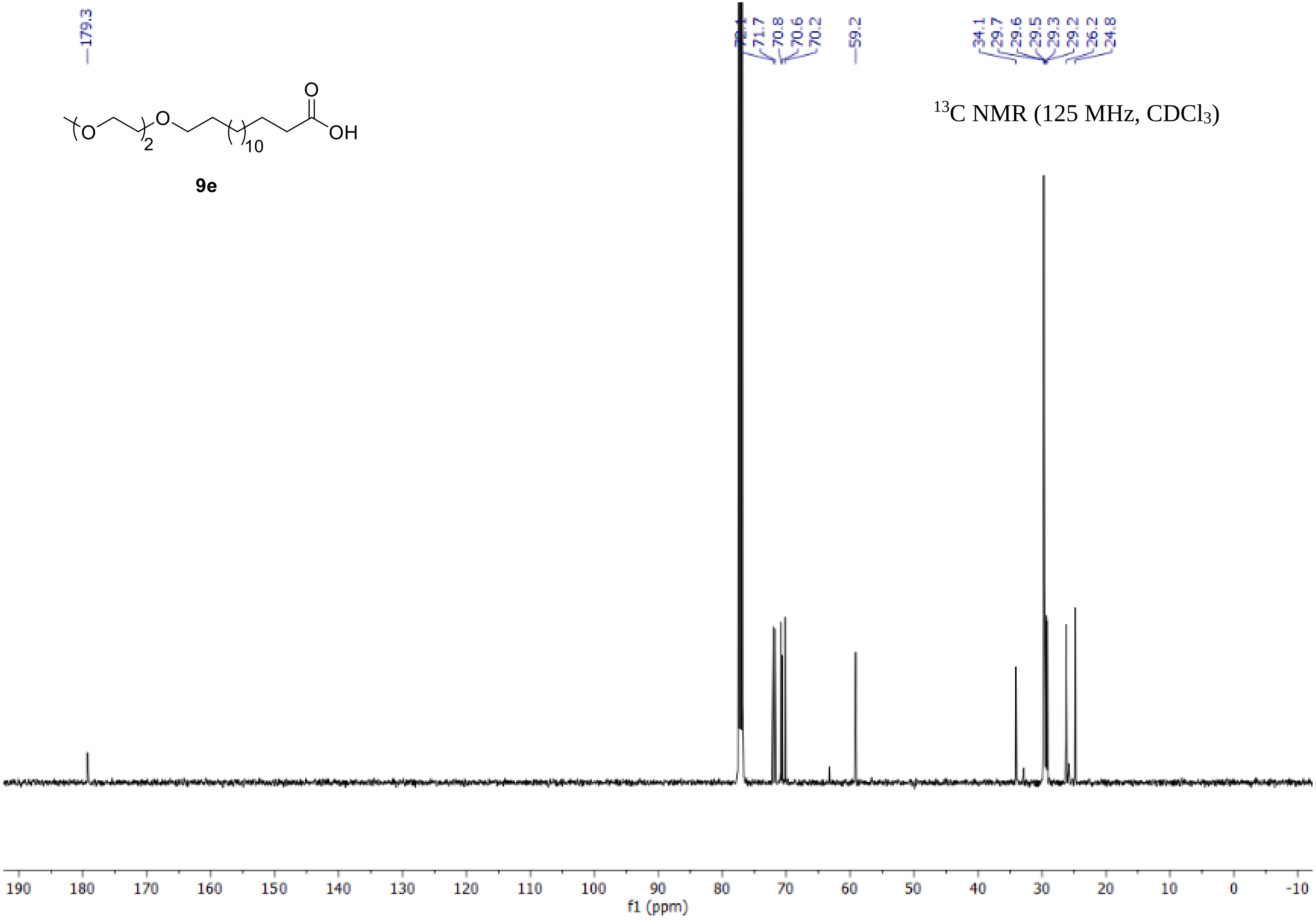
179.3
77.1
71.7
70.8
70.6
70.2
59.2
34.1
29.7
29.6
29.5
29.3
29.2
26.2
24.8
O
O
OH
O
2
10
9e
13C NMR (125 MHz, CDCl3)
190
180
170
160
150
140
130
120
110
100
90
80
70
60
50
40
30
20
10
0
-10
f1 (ppm)

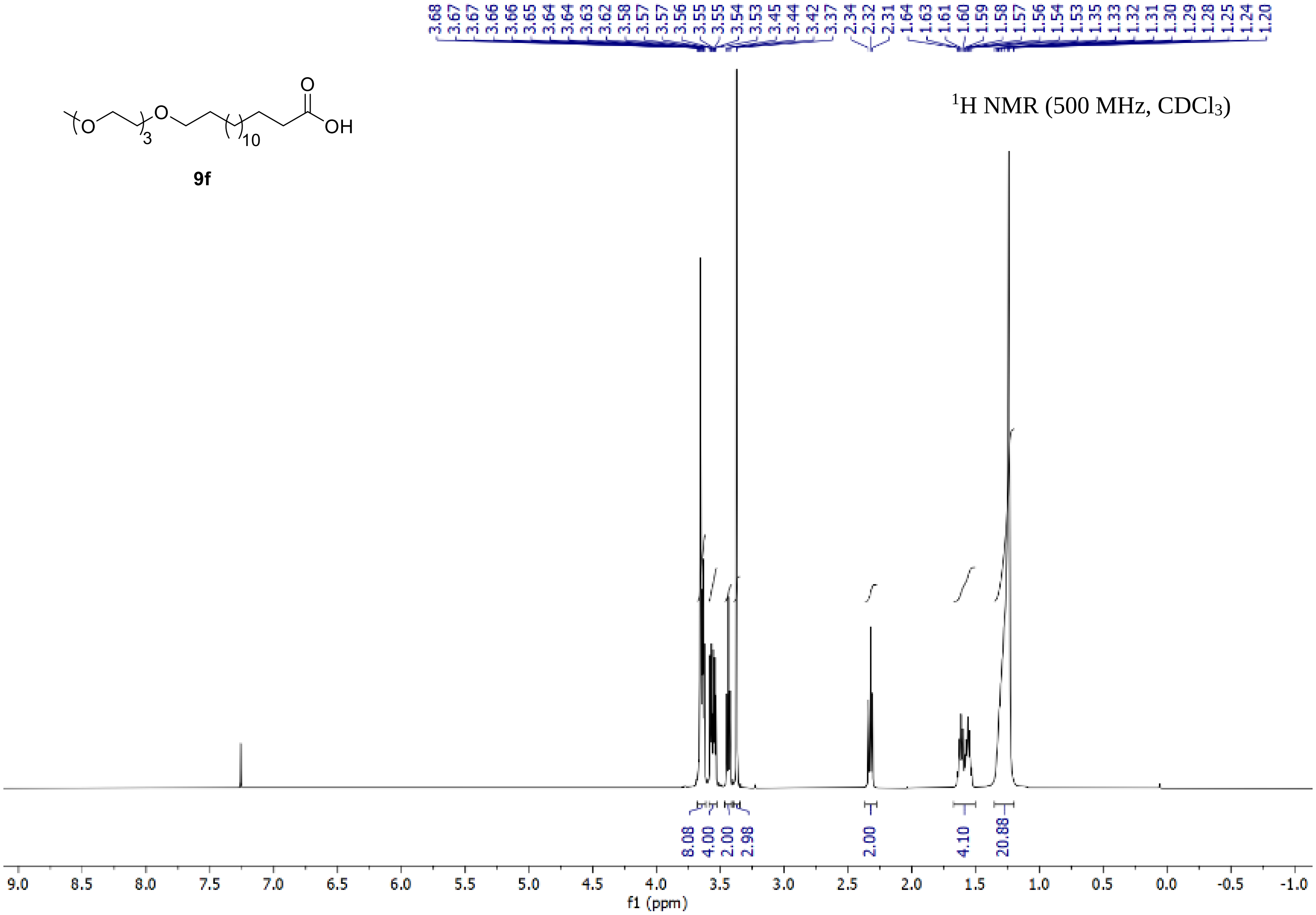
9f
1H NMR (500 MHz, CDCl3)
3.68 3.67 3.67 3.66 3.66 3.65 3.64 3.64 3.63 3.62 3.58 3.57 3.57 3.56 3.55 3.55 3.54 3.53 3.45 3.44 3.42 3.37 2.34 2.32 2.31 1.64 1.63 1.61 1.60 1.59 1.58 1.57 1.56 1.54 1.53 1.35 1.33 1.32 1.31 1.30 1.29 1.28 1.25 1.24 1.20
8.08 4.00 2.00 2.98 2.00 4.10 20.88
9.0 8.5 8.0 7.5 7.0 6.5 6.0 5.5 5.0 4.5 4.0 3.5 3.0 2.5 2.0 1.5 1.0 0.5 0.0 -0.5 -1.0
f1 (ppm)

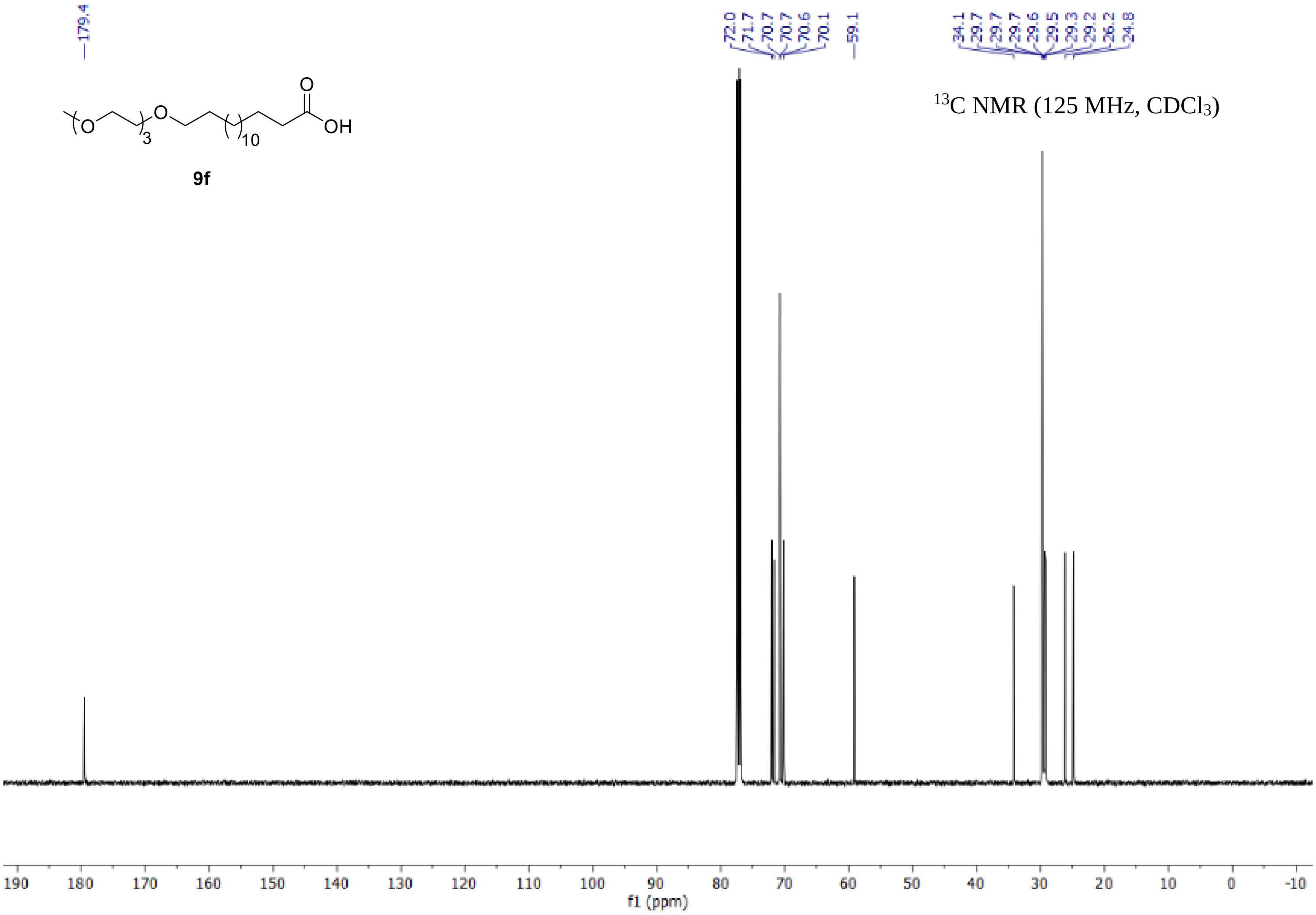

^{13}C NMR (125 MHz, $CDCl_3$)

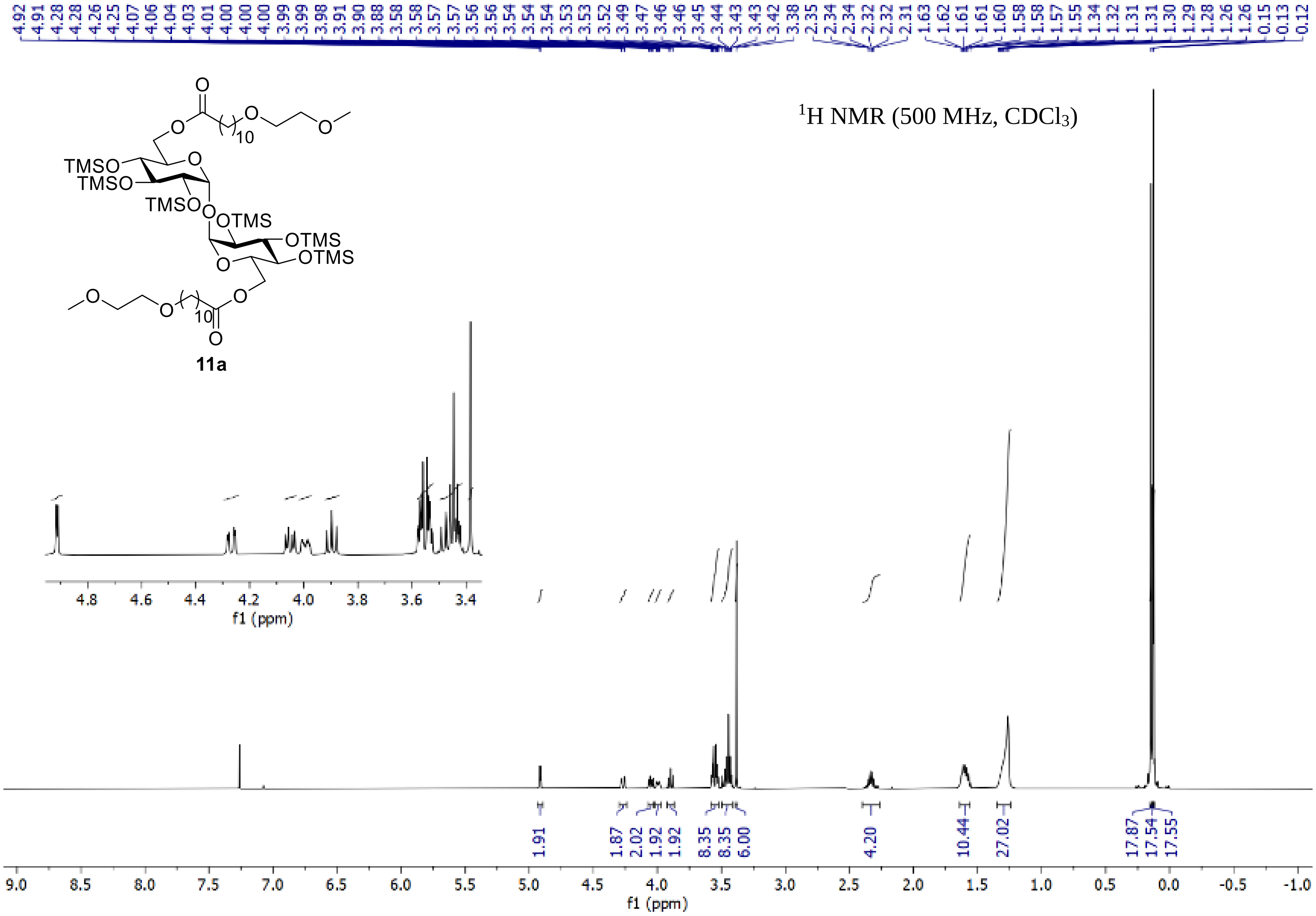

¹H NMR (500 MHz, CDCl₃)
11a
f1 (ppm)

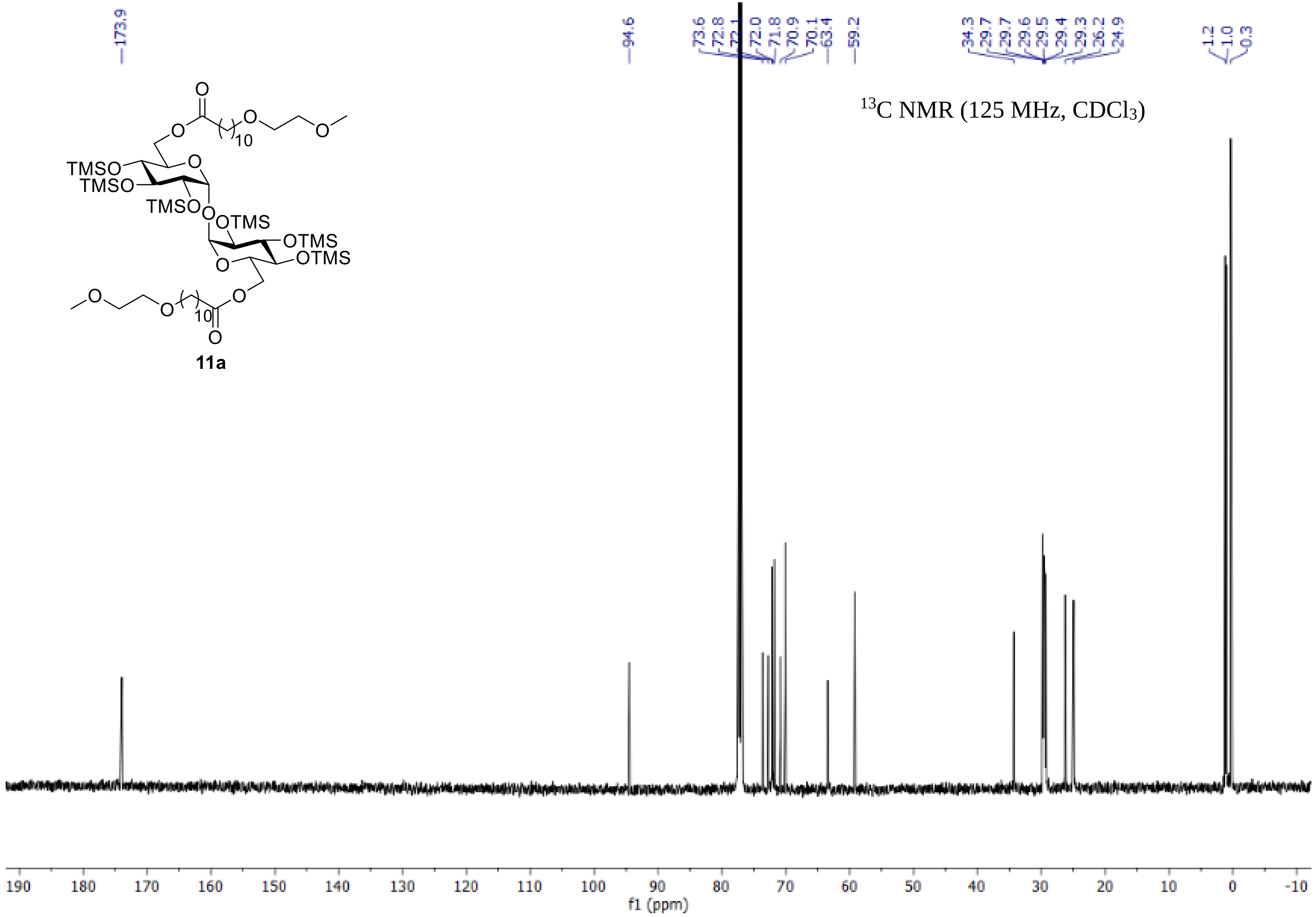

13C NMR (125 MHz, CDCl3)
11a
f1 (ppm)

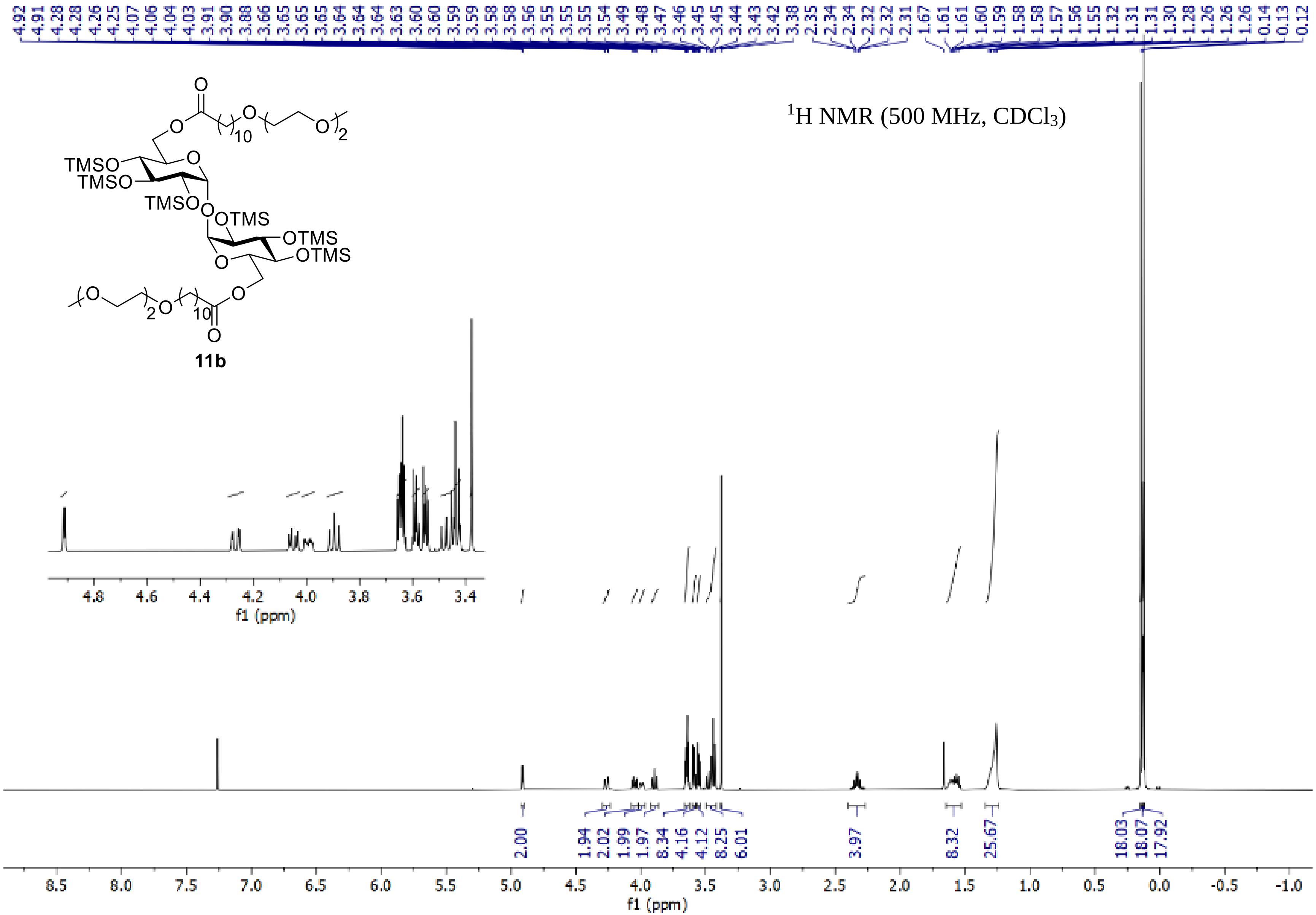
¹H NMR (500 MHz, CDCl₃)
11b
f1 (ppm)

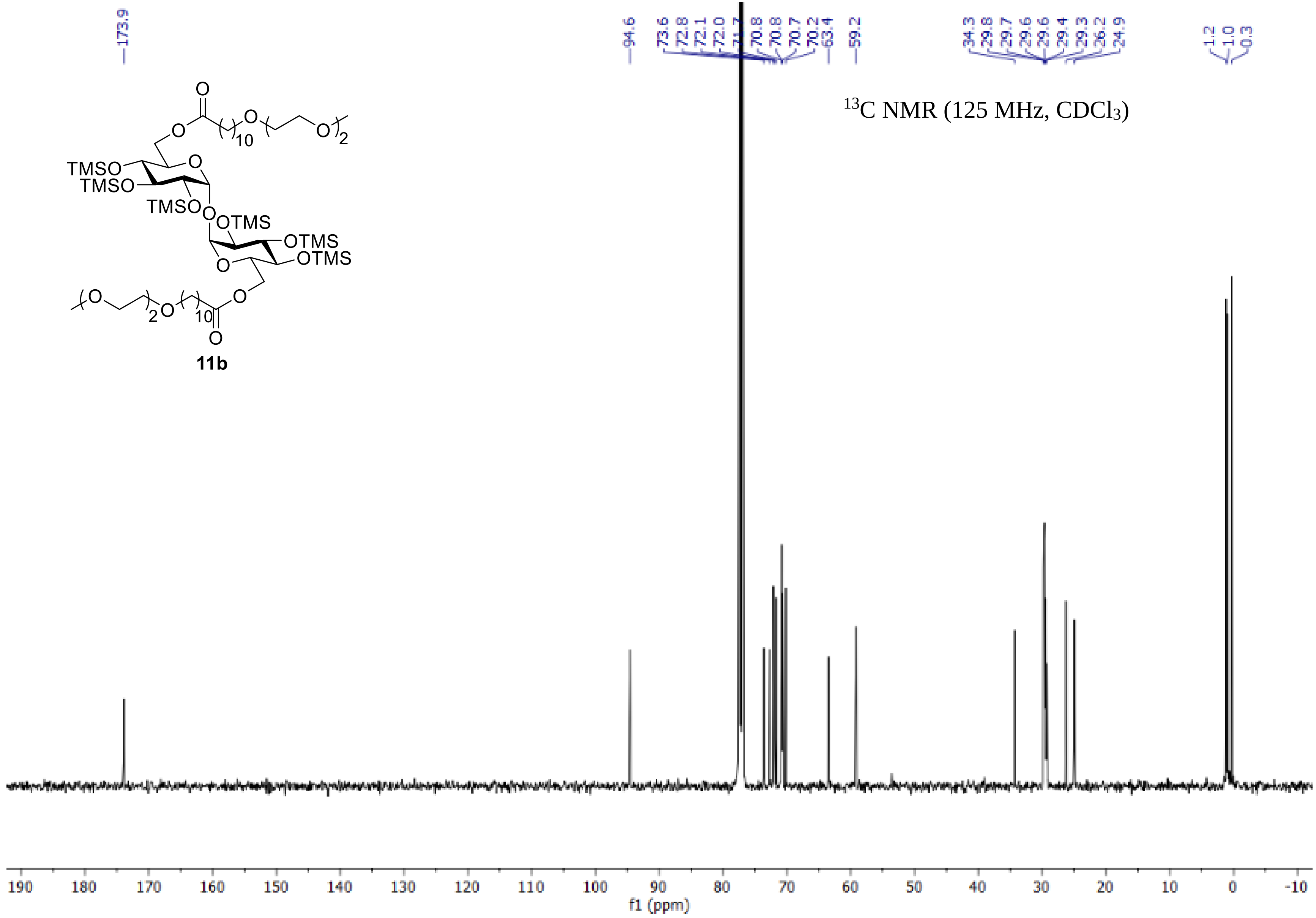
173.9
94.6
73.6
72.8
72.1
72.0
71.7
70.8
70.8
70.7
70.2
63.4
59.2
34.3
29.8
29.7
29.6
29.6
29.4
29.3
26.2
24.9
1.2
1.0
0.3
13C NMR (125 MHz, CDCl3)
TMSO
TMSO
TMSO
OTMS
OTMS
OTMS
11b
f1 (ppm)

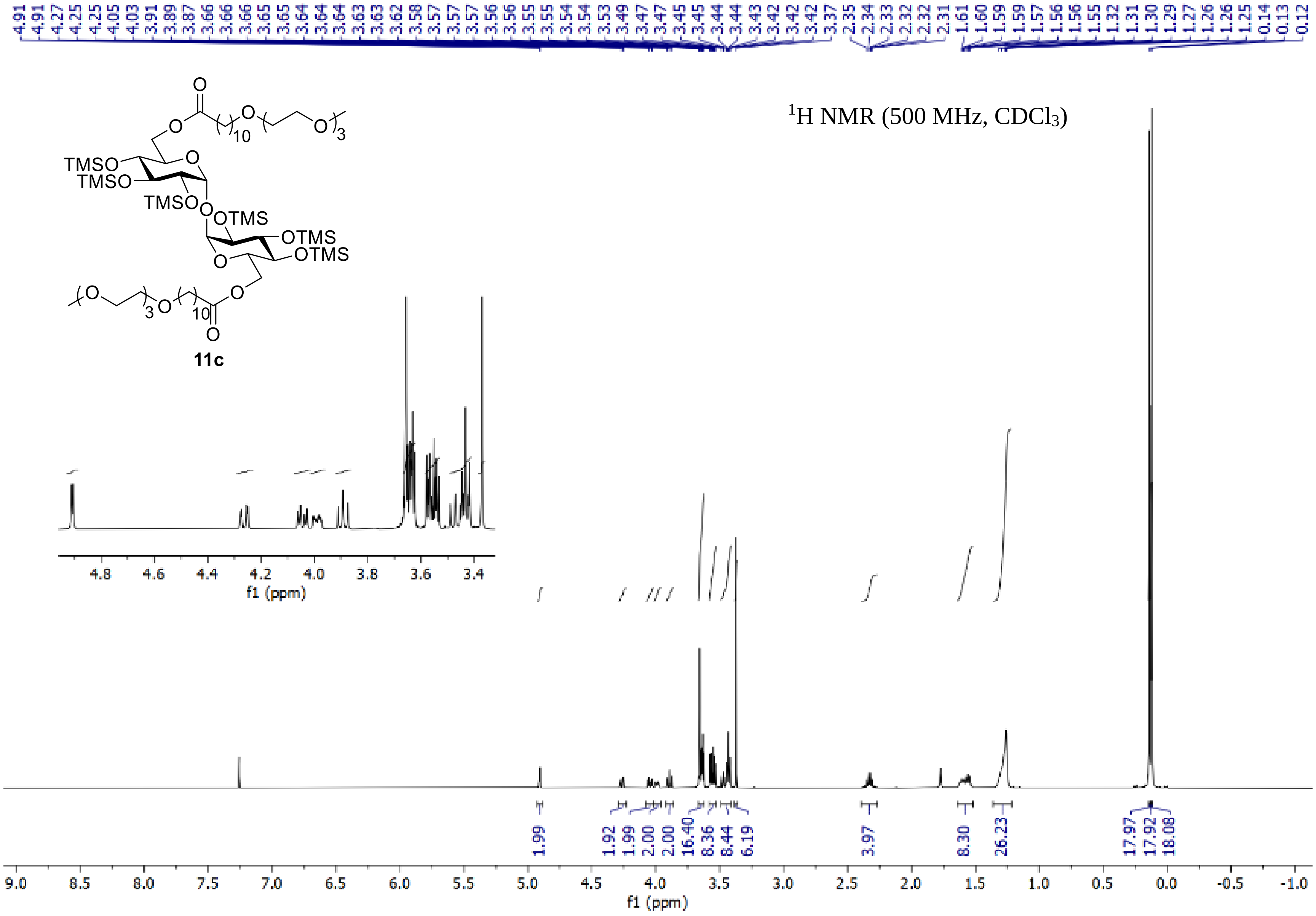

^{1}H NMR (500 MHz, CDCl$_3$)
11c
f1 (ppm)

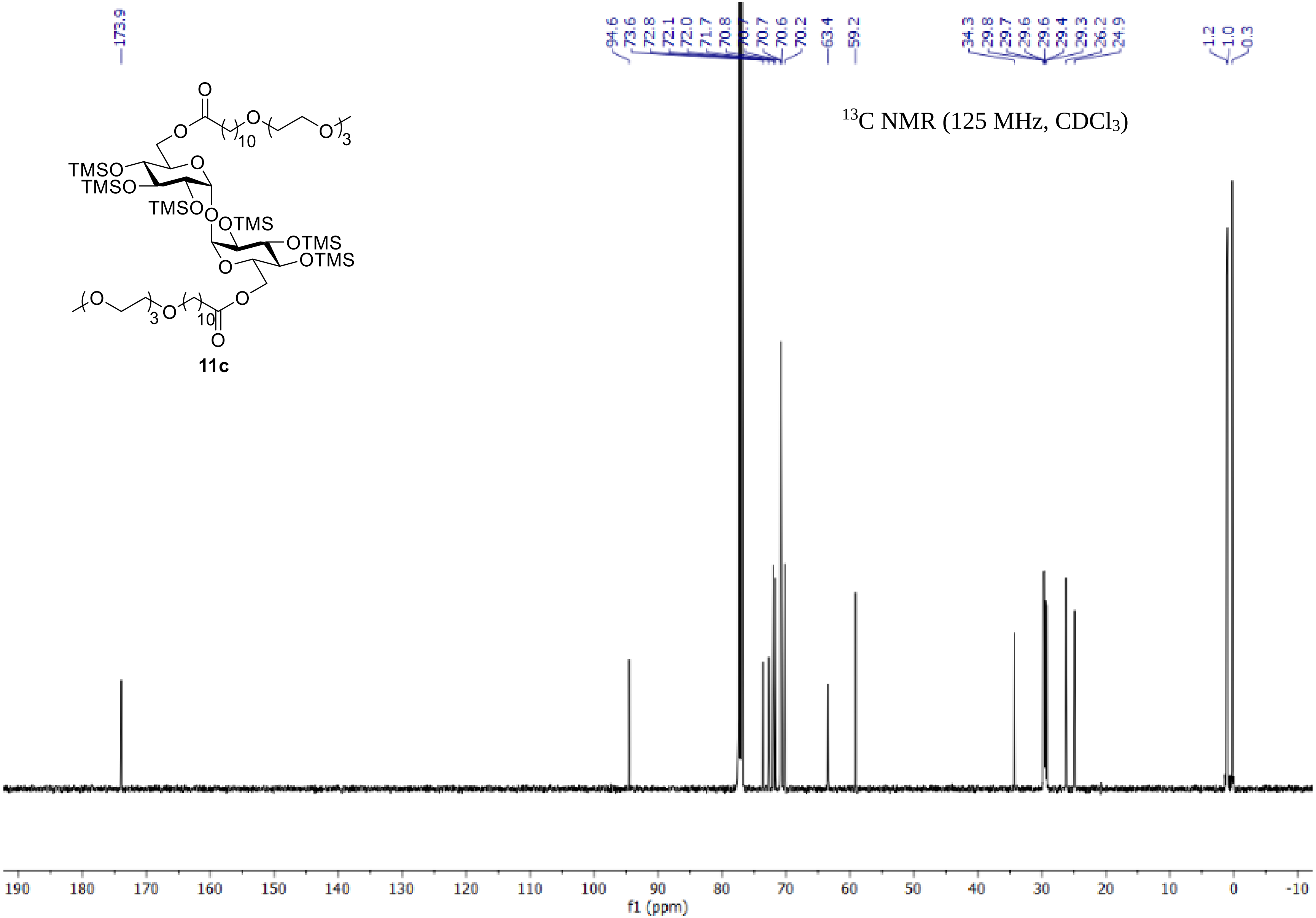

^{13}C NMR (125 MHz, CDCl$_3$)
173.9
94.6
73.6
72.8
72.1
72.0
71.7
70.8
70.7
70.7
70.6
70.2
63.4
59.2
34.3
29.8
29.7
29.6
29.6
29.4
29.3
26.2
24.9
1.2
1.0
0.3
f1 (ppm)

11c

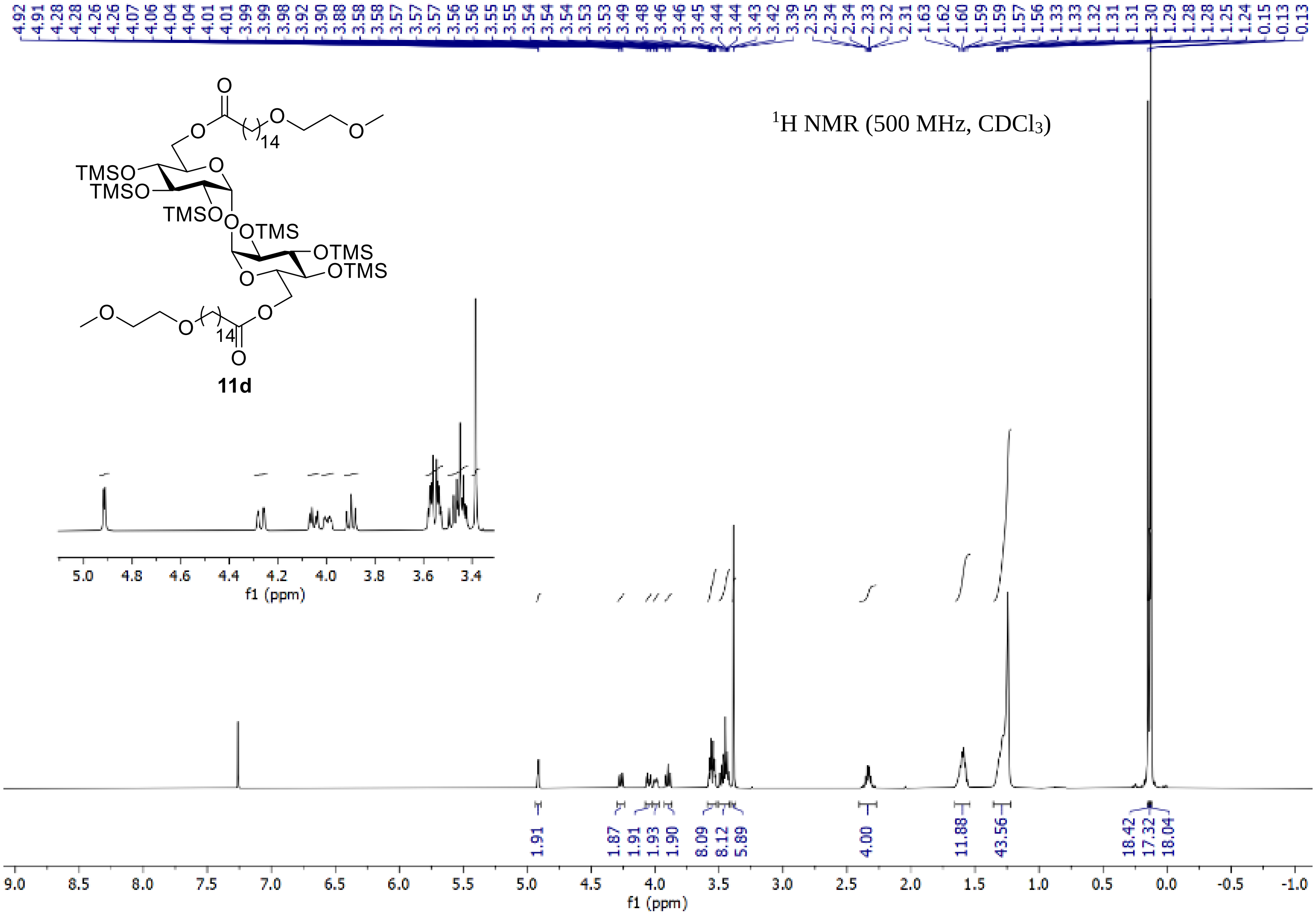

^{1}H NMR (500 MHz, CDCl3)
11d
f1 (ppm)

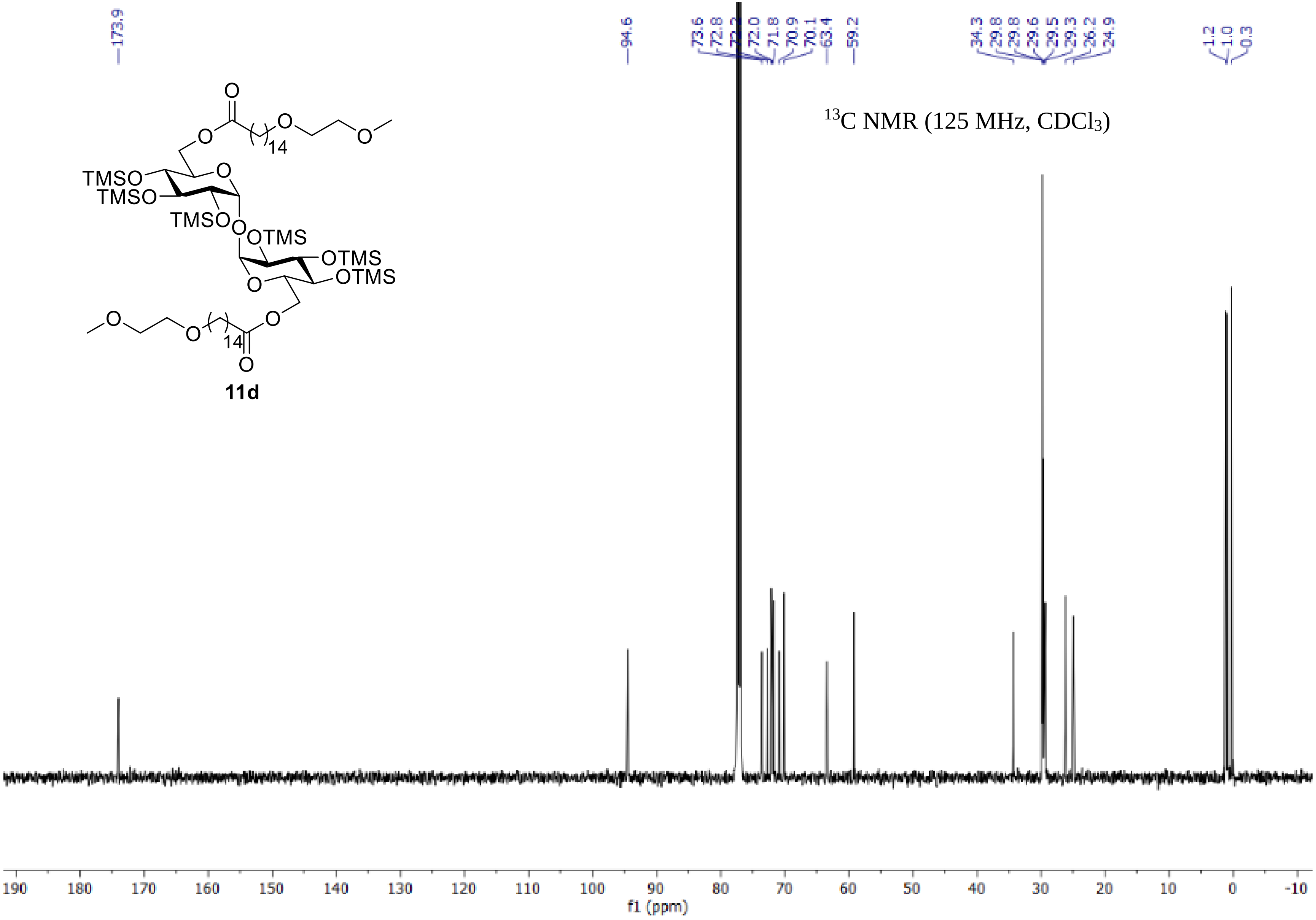
13C NMR (125 MHz, CDCl3)
173.9
94.6
73.6
72.8
72.0
71.8
70.9
70.1
63.4
59.2
34.3
29.8
29.8
29.6
29.5
29.3
26.2
24.9
1.2
1.0
0.3
TMSO
TMSO
TMSO
OTMS
OTMS
OTMS
14
14
11d
190 180 170 160 150 140 130 120 110 100 90 80 70 60 50 40 30 20 10 0 -10
f1 (ppm)

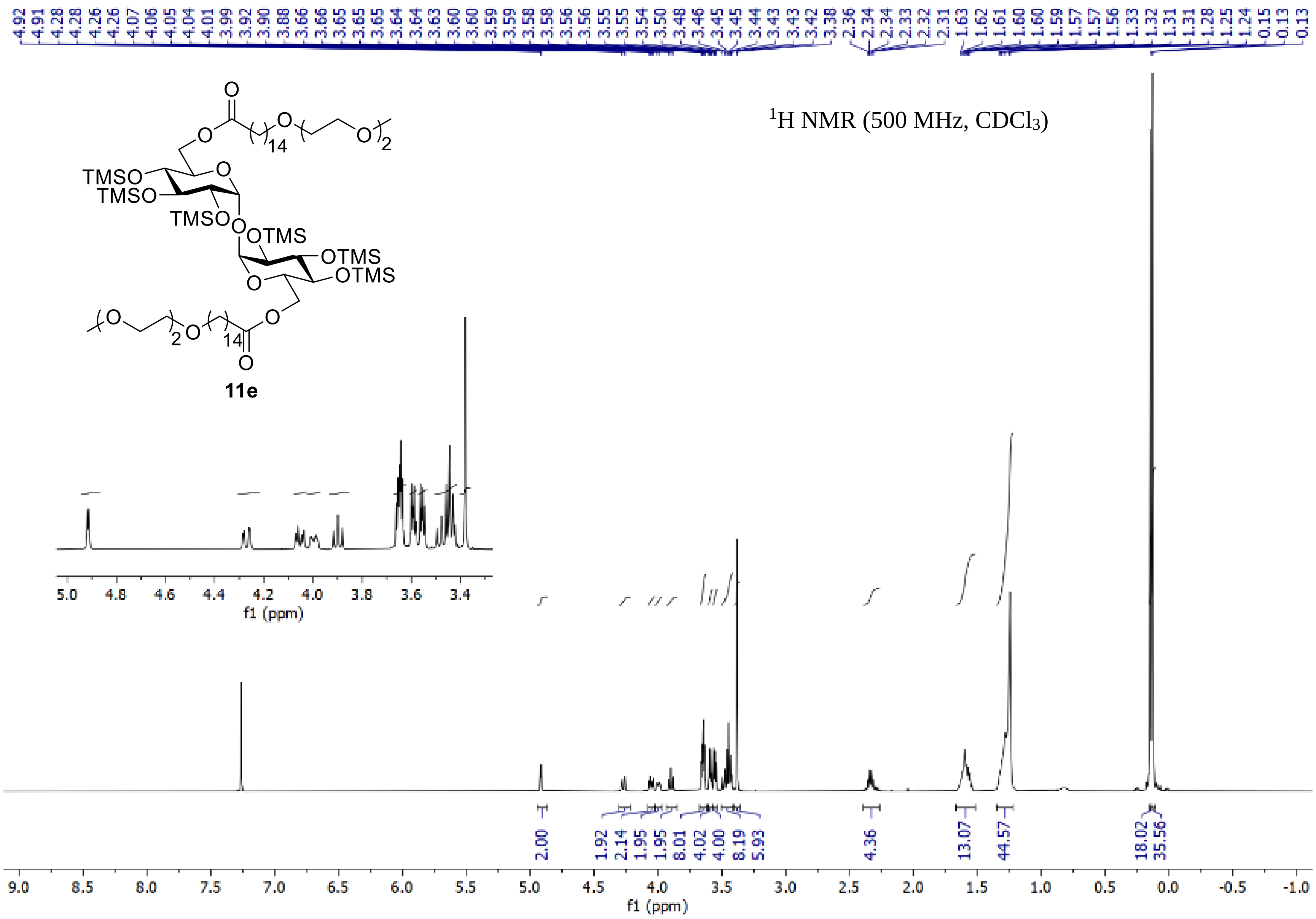

11e

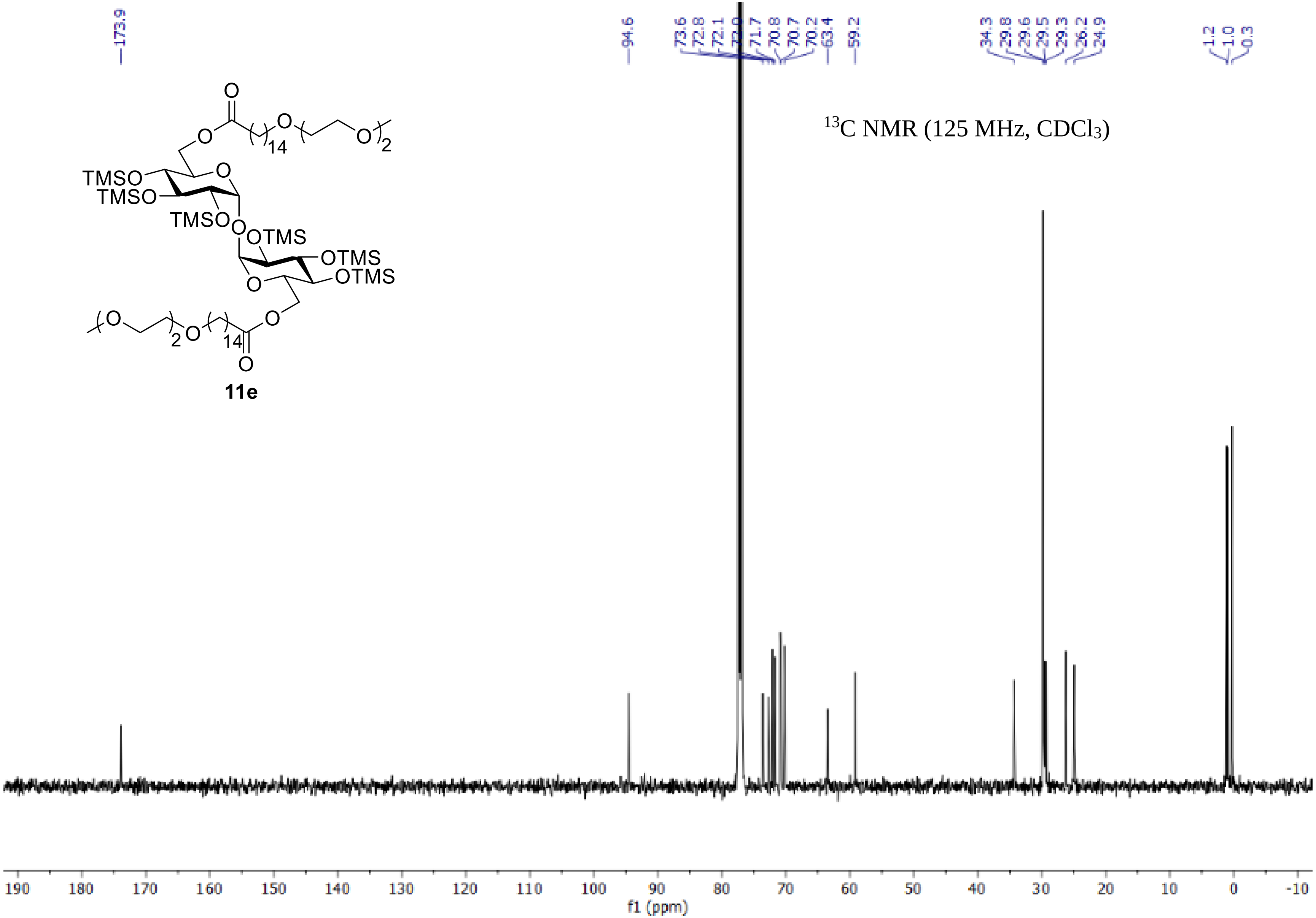

173.9
94.6
73.6
72.8
72.1
71.7
70.8
70.7
70.2
63.4
59.2
34.3
29.8
29.6
29.5
29.3
26.2
24.9
1.2
1.0
0.3
13C NMR (125 MHz, CDCl3)
TMSO
TMSO
TMSO
OTMS
OTMS
OTMS
11e
190 180 170 160 150 140 130 120 110 100 90 80 70 60 50 40 30 20 10 0 -10
f1 (ppm)

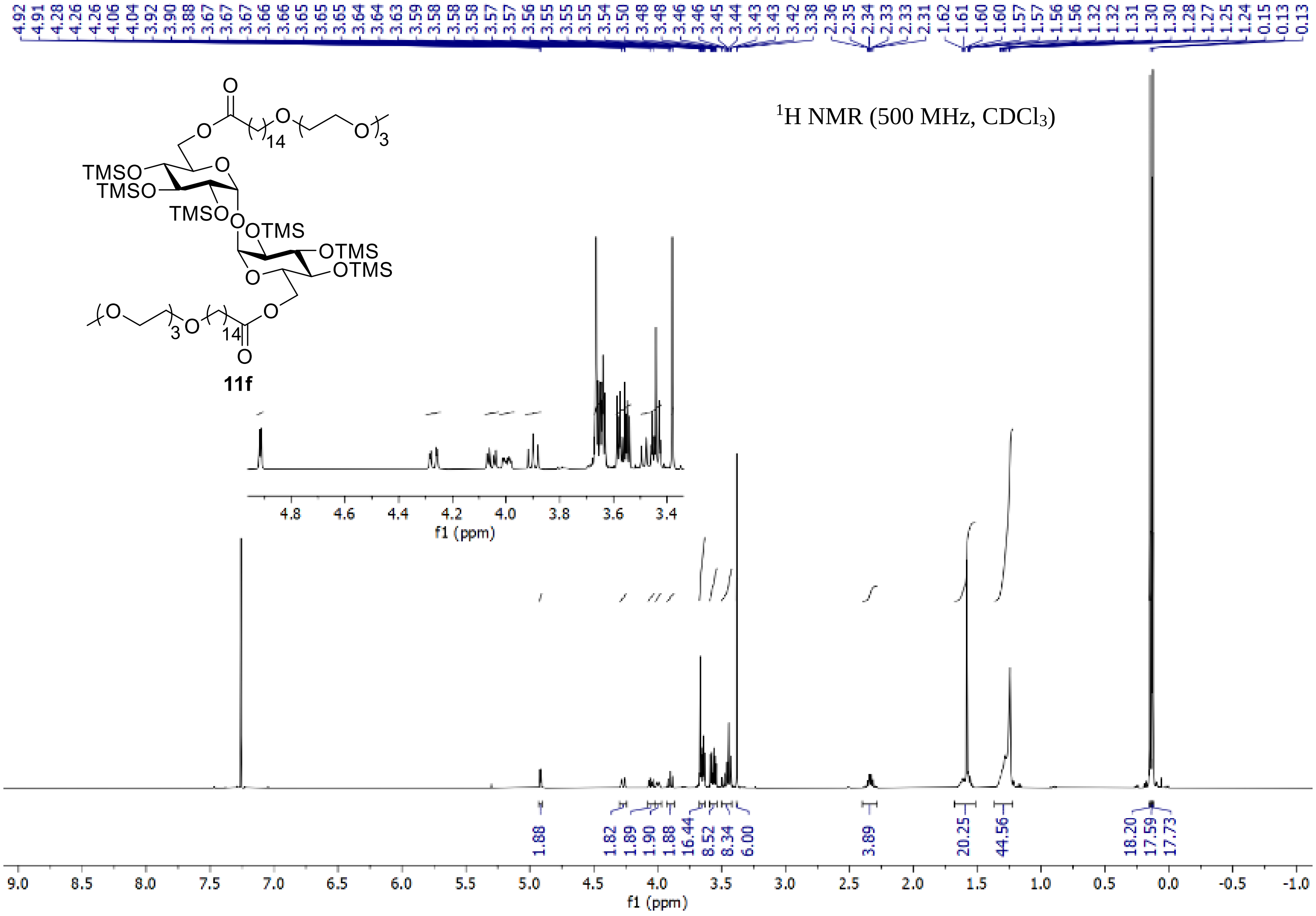

^{1}H NMR (500 MHz, CDCl3)
11f
f1 (ppm)

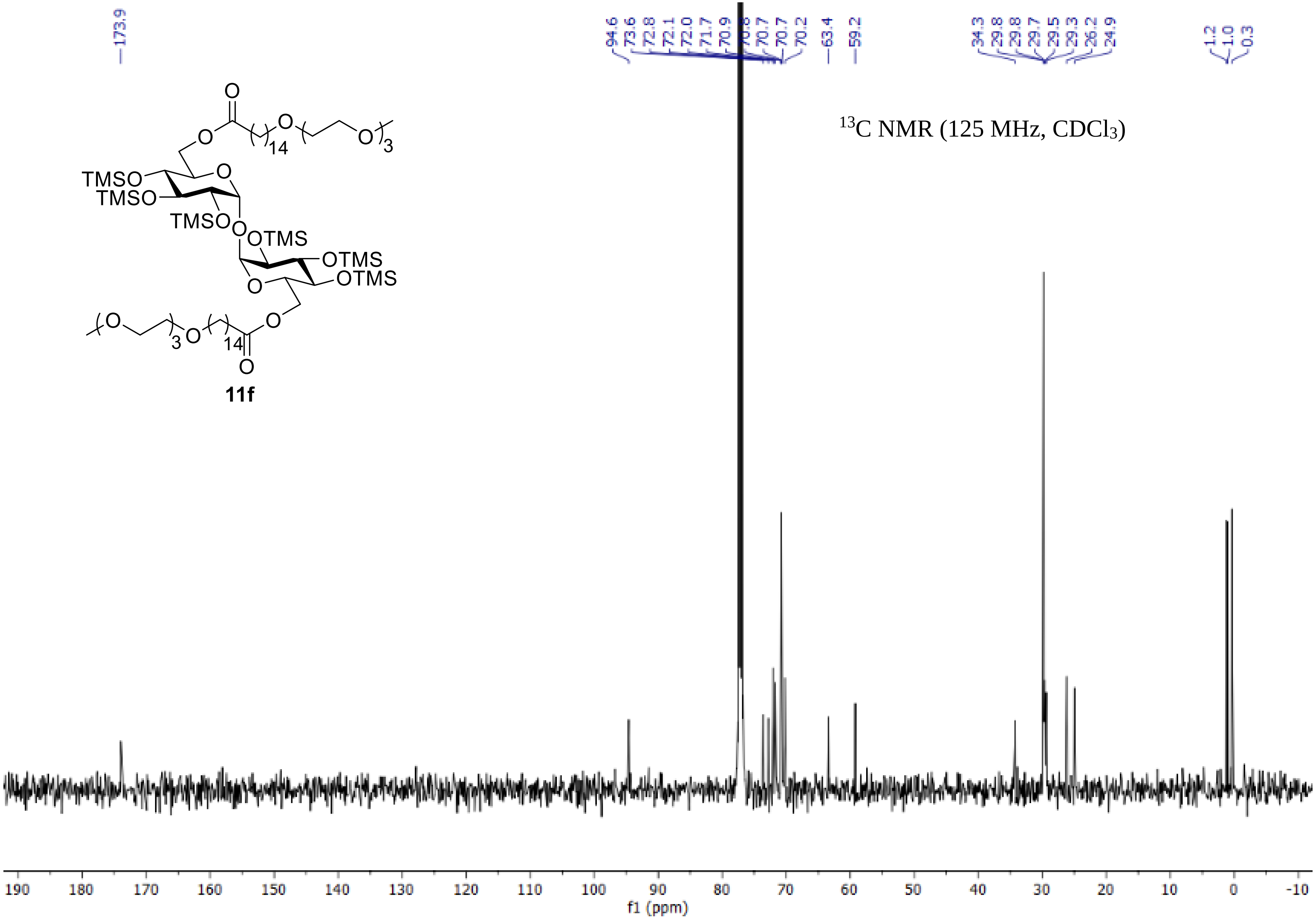

173.9
94.6
73.6
72.8
72.1
72.0
71.7
70.9
70.8
70.7
70.7
70.2
63.4
59.2
34.3
29.8
29.8
29.7
29.5
29.3
26.2
24.9
1.2
1.0
0.3
TMSO
TMSO
TMSO
OTMS
OTMS
OTMS
11f
13C NMR (125 MHz, CDCl3)
f1 (ppm)

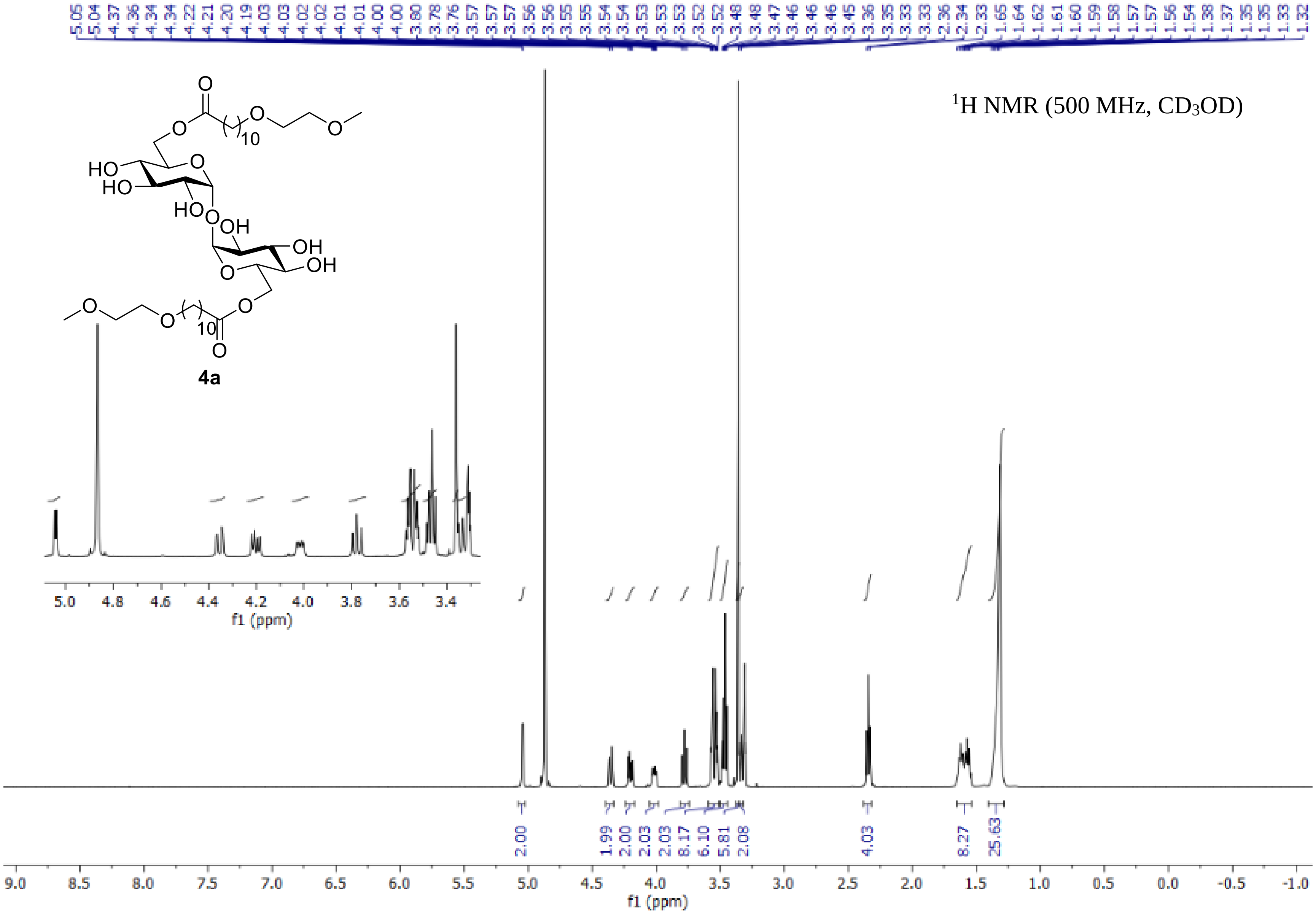

^{1}H NMR (500 MHz, CD3OD)
4a
f1 (ppm)

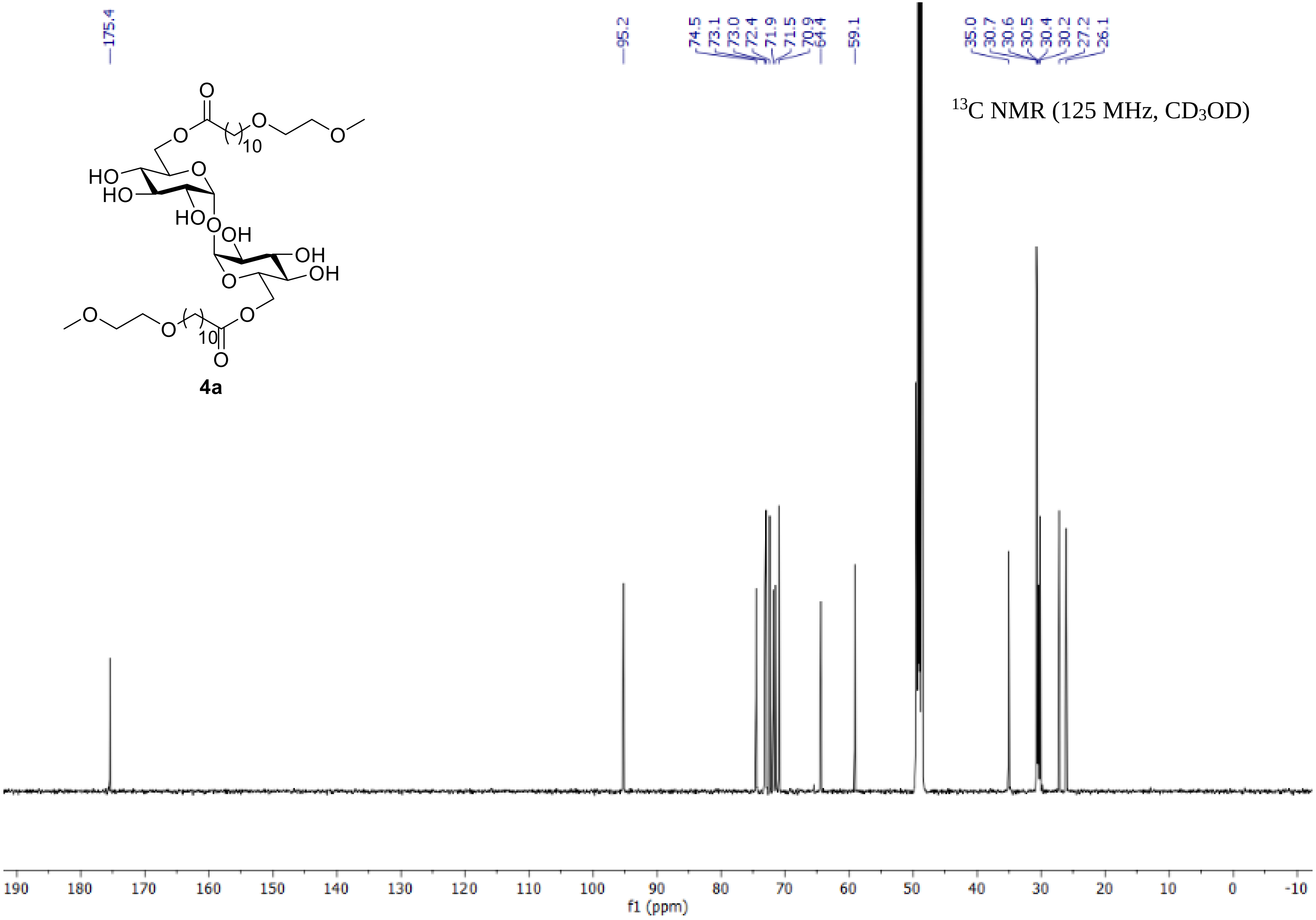
175.4
95.2
74.5 73.1 73.0 72.4 71.9 71.5 70.9 64.4
59.1
35.0 30.7 30.6 30.5 30.4 30.2 27.2 26.1
^{13}C NMR (125 MHz, CD$_3$OD)
4a
190 180 170 160 150 140 130 120 110 100 90 80 70 60 50 40 30 20 10 0 -10
f1 (ppm)

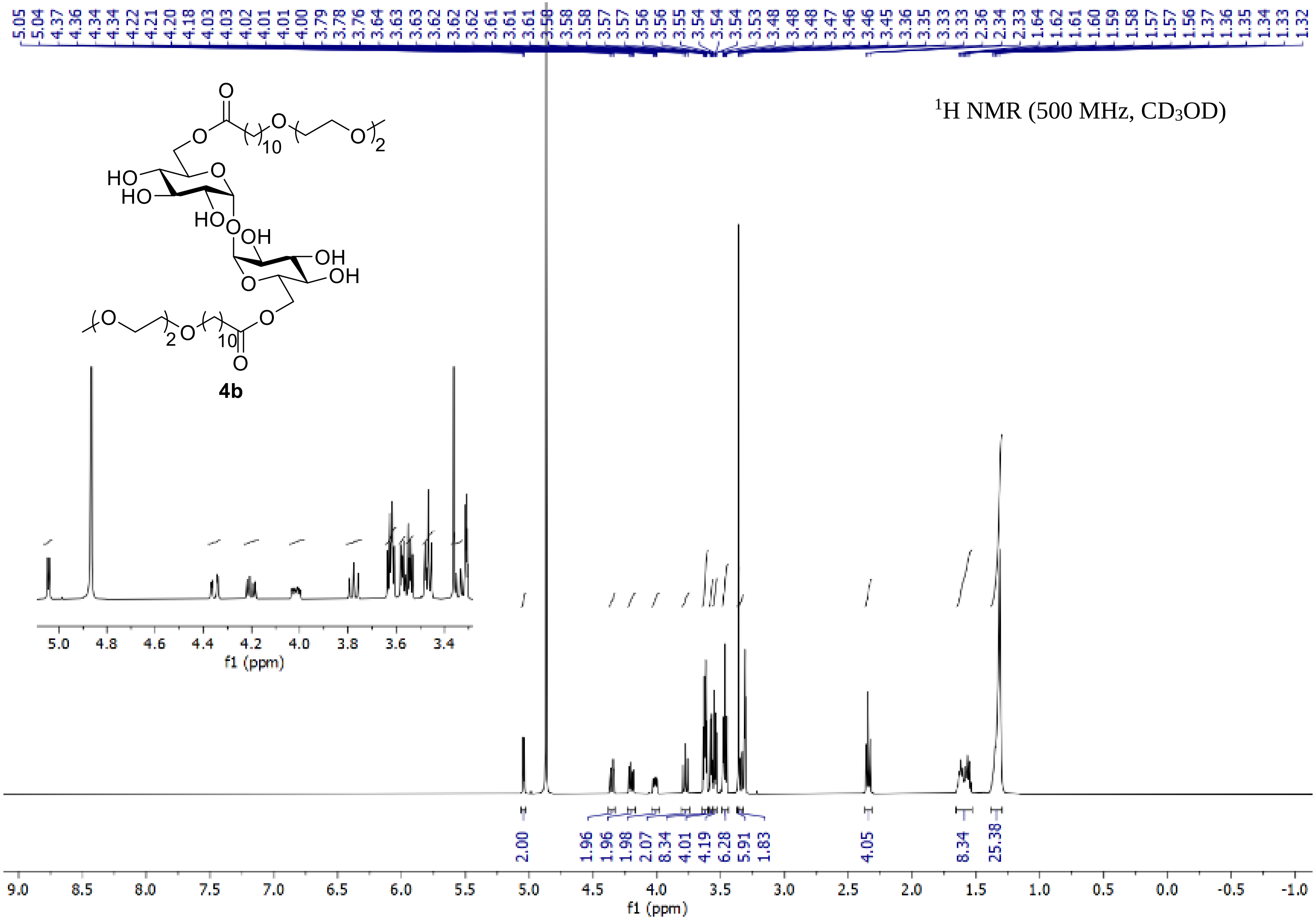

1H NMR (500 MHz, CD3OD)
4b
f1 (ppm)

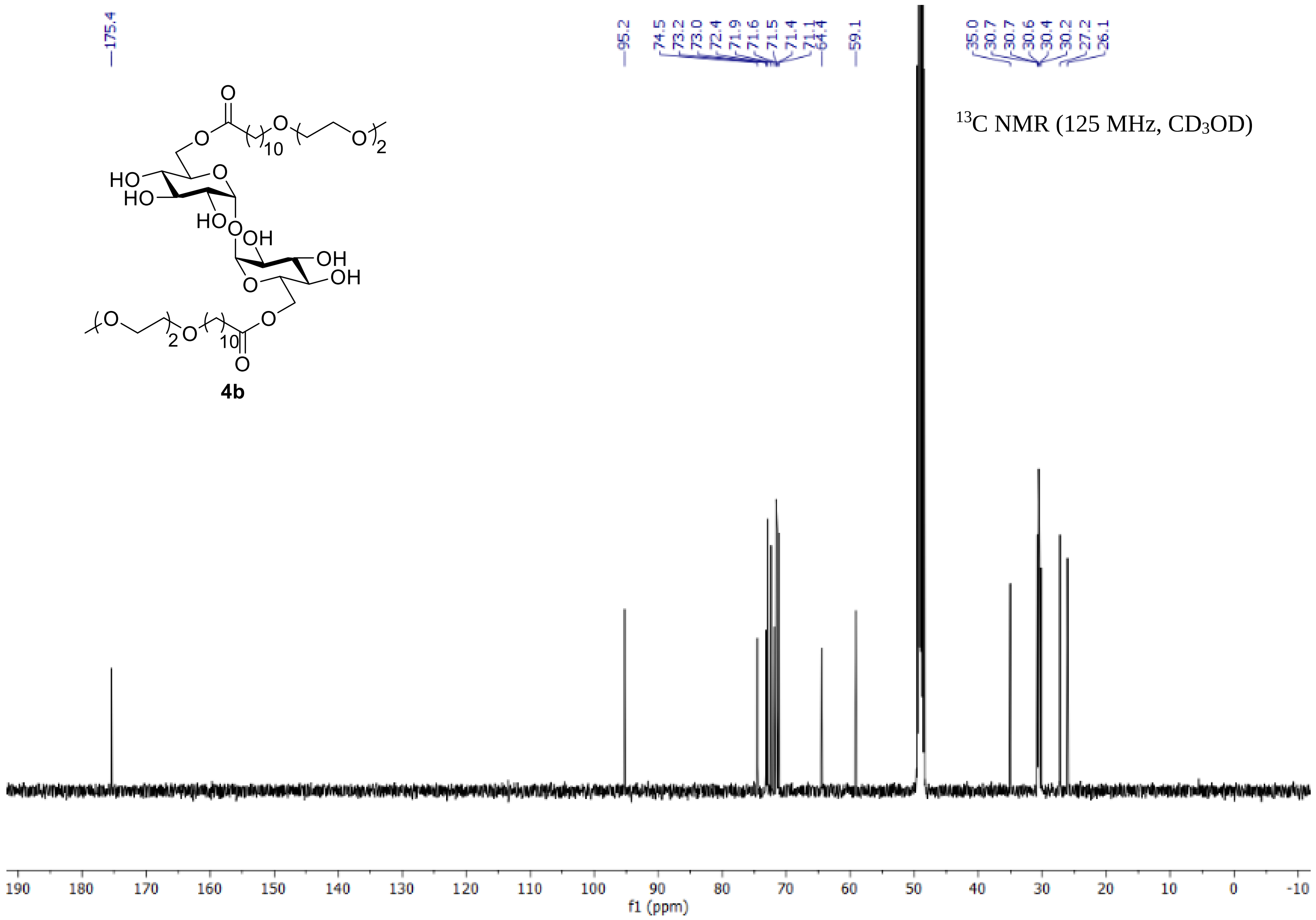
175.4
95.2
74.5
73.2
73.0
72.4
71.9
71.6
71.5
71.4
71.1
64.4
59.1
35.0
30.7
30.7
30.6
30.4
30.2
27.2
26.1
13C NMR (125 MHz, CD3OD)
4b
f1 (ppm)

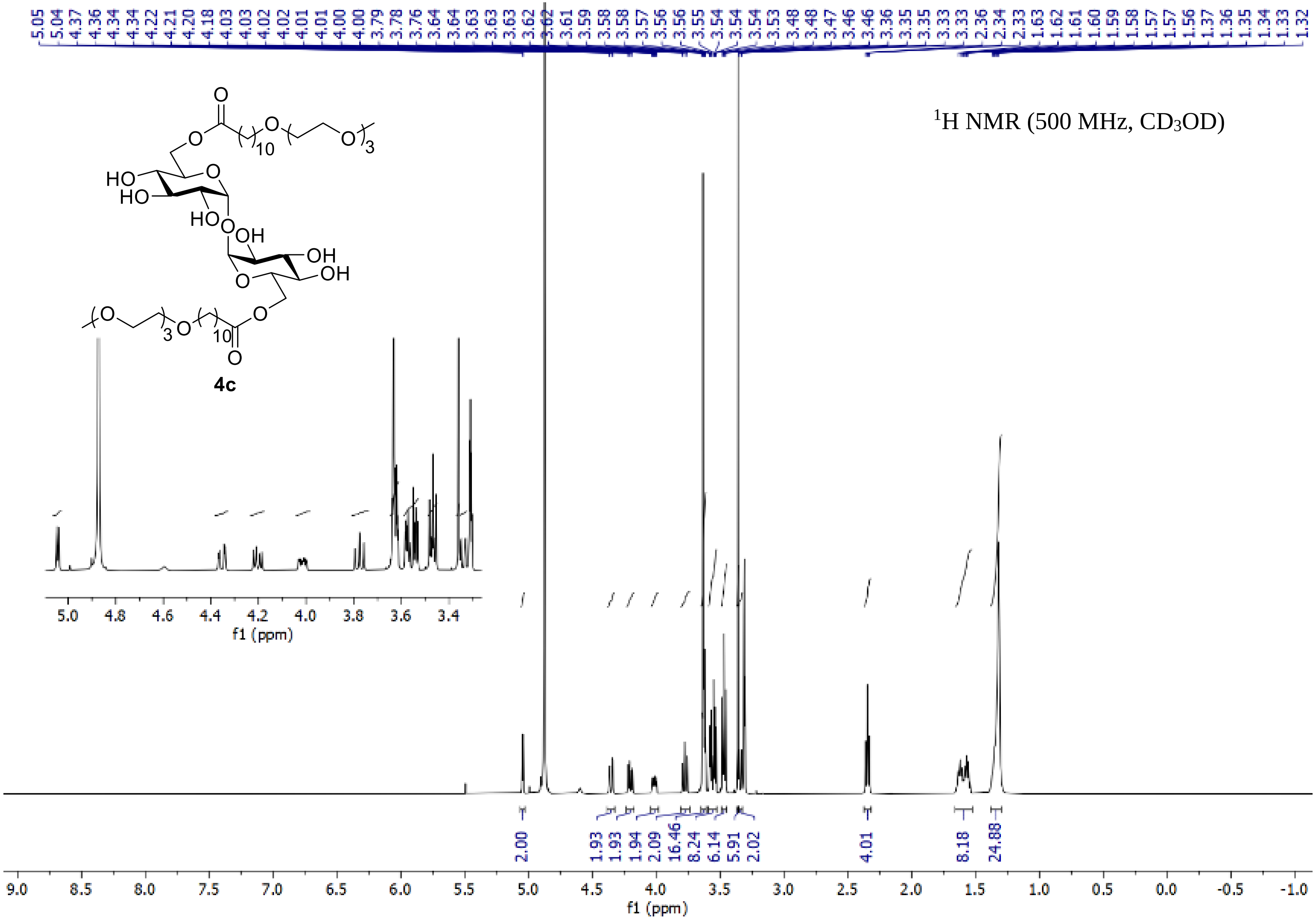

^{1}H NMR (500 MHz, CD_3OD)
4c
f1 (ppm)

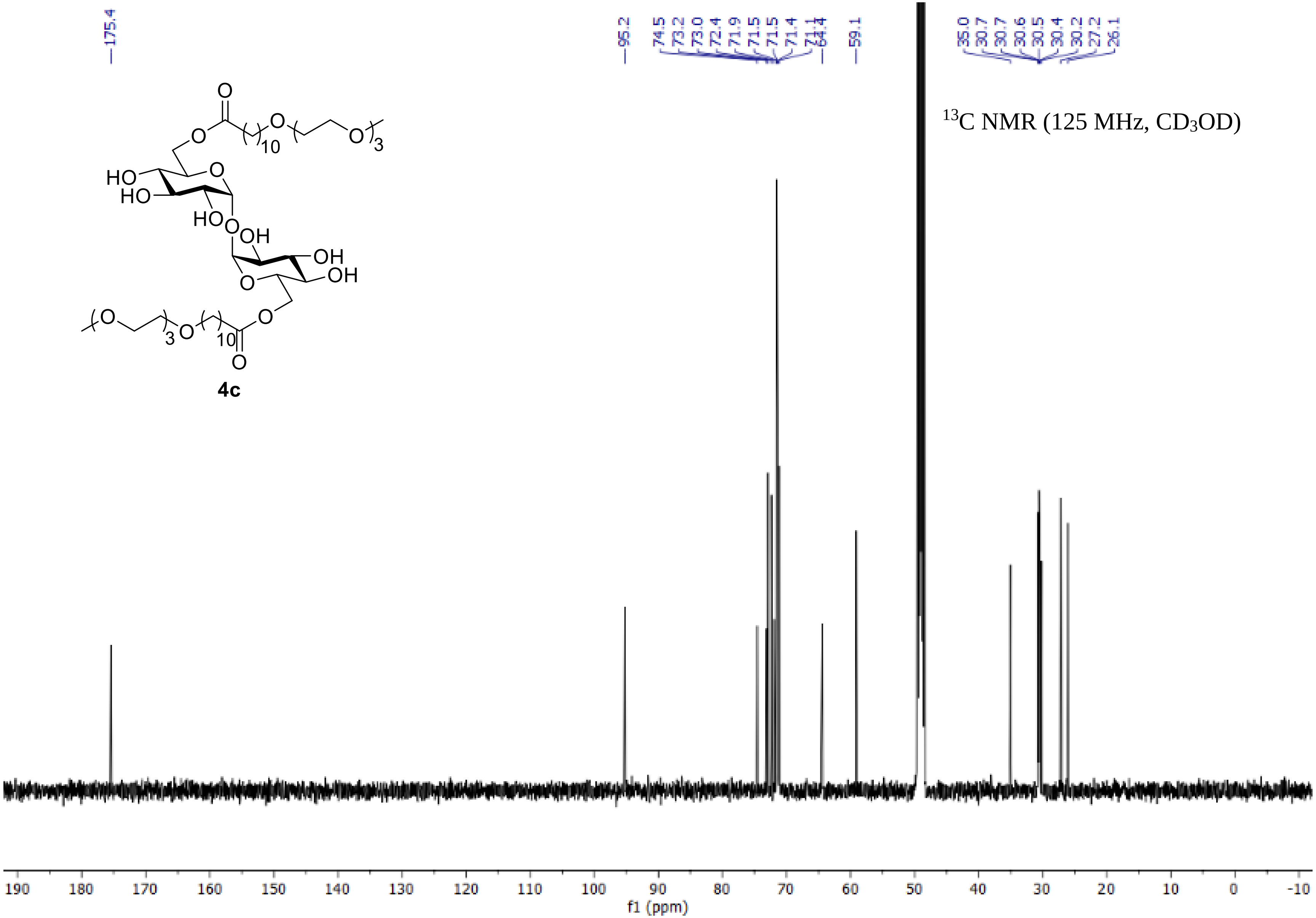

175.4
95.2
74.5
73.2
73.0
72.4
71.9
71.5
71.5
71.4
71.1
64.4
59.1
35.0
30.7
30.7
30.6
30.5
30.4
30.2
27.2
26.1
13C NMR (125 MHz, CD3OD)
4c
f1 (ppm)

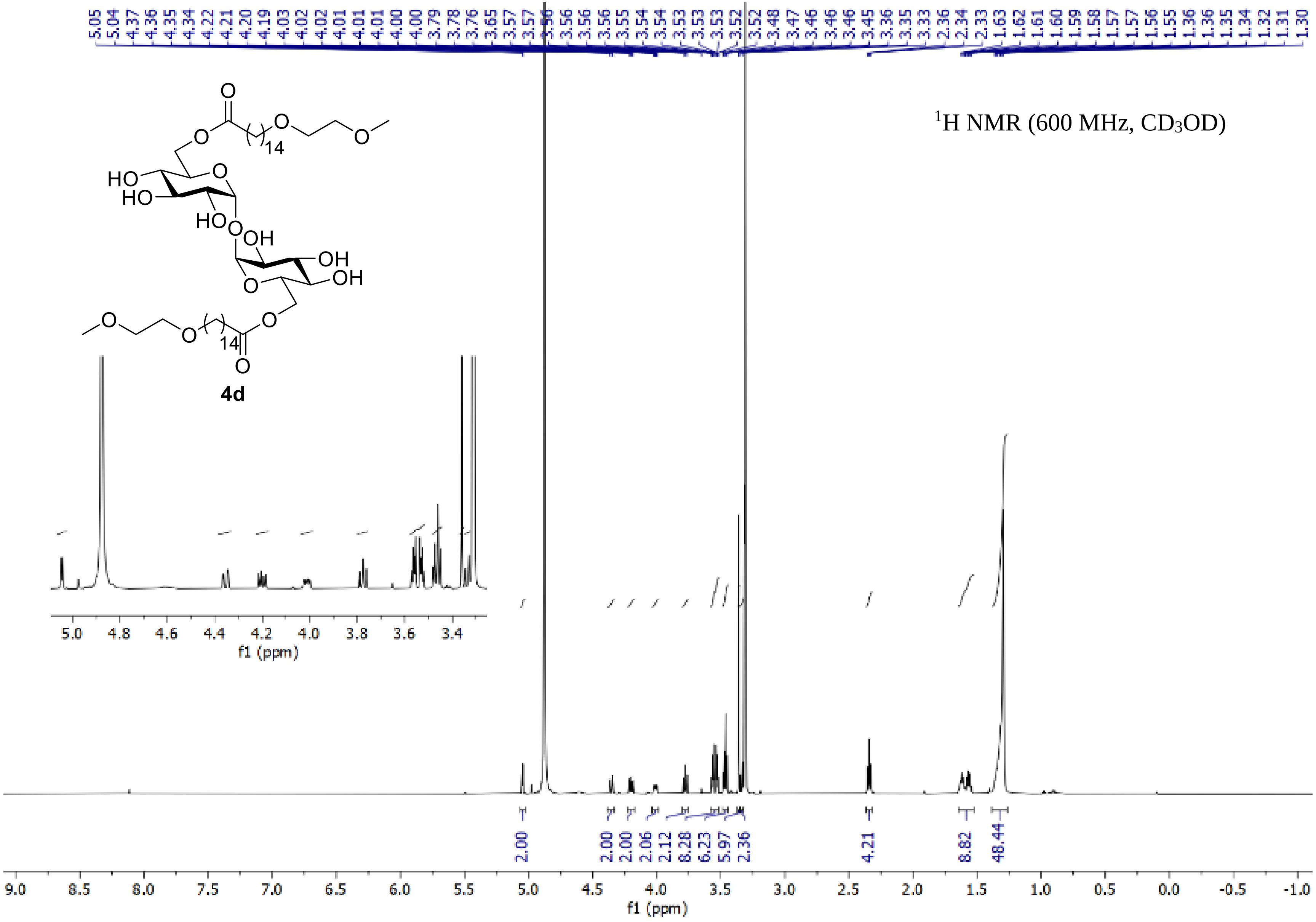

^{1}H NMR (600 MHz, CD_3OD)
4d
f1 (ppm)

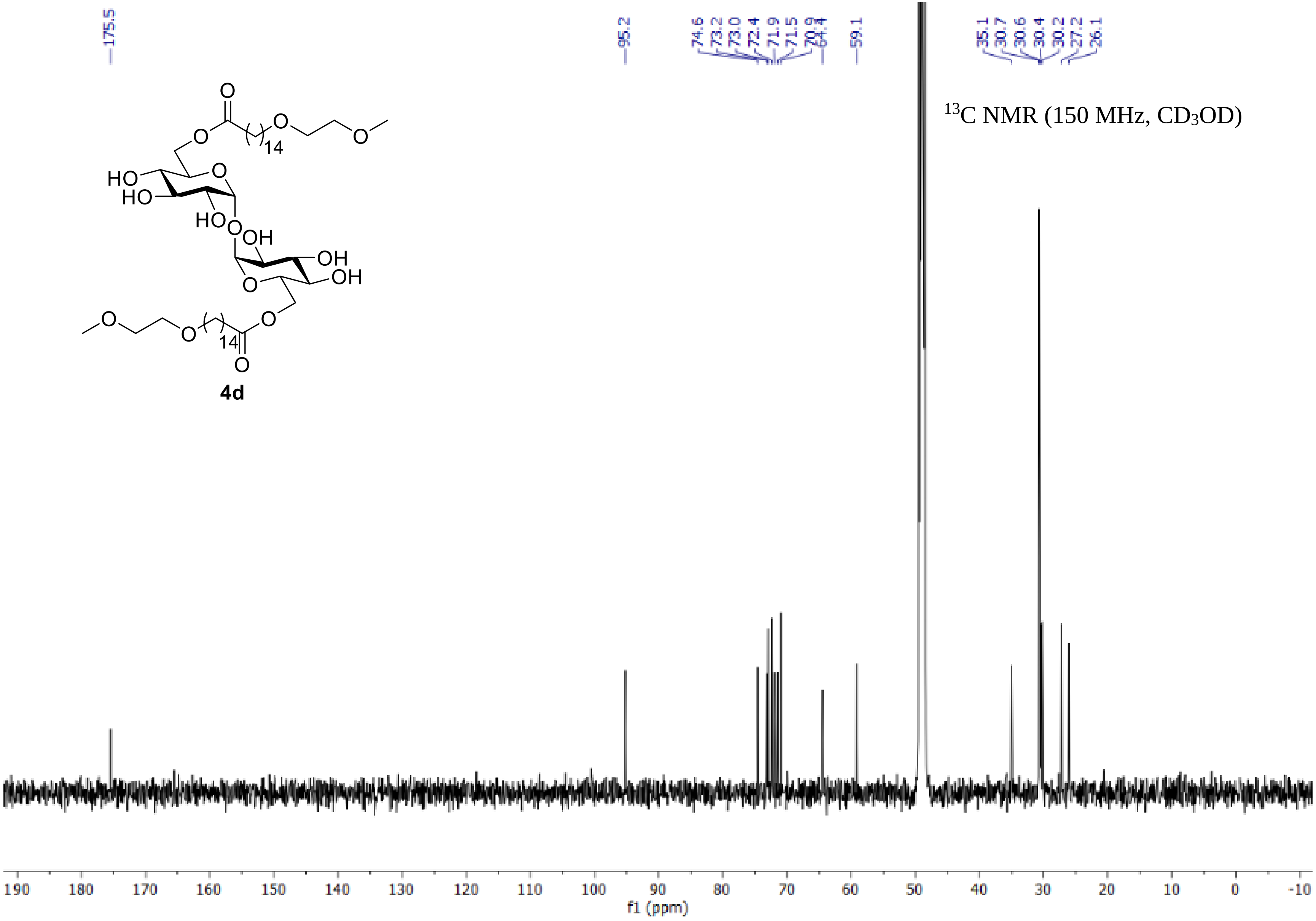

4d

^{13}C NMR (150 MHz, CD_3OD)

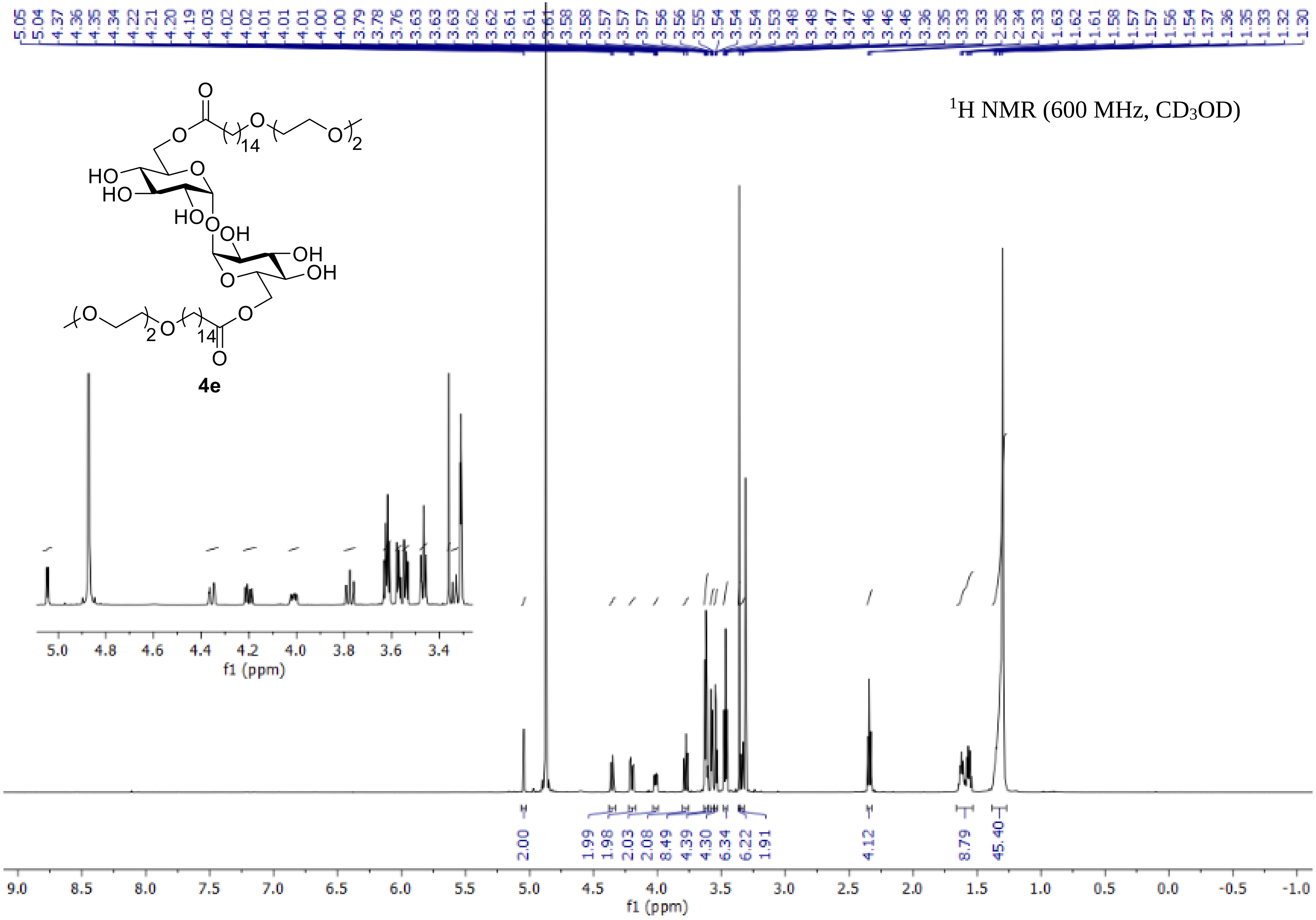

^{1}H NMR (600 MHz, CD3OD)
4e
f1 (ppm)

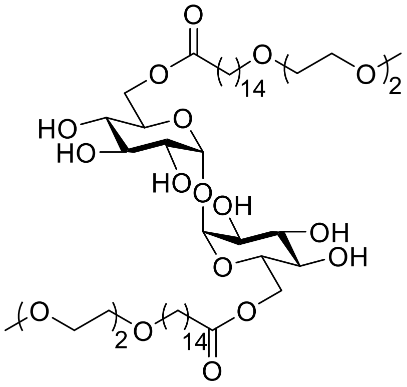

4e

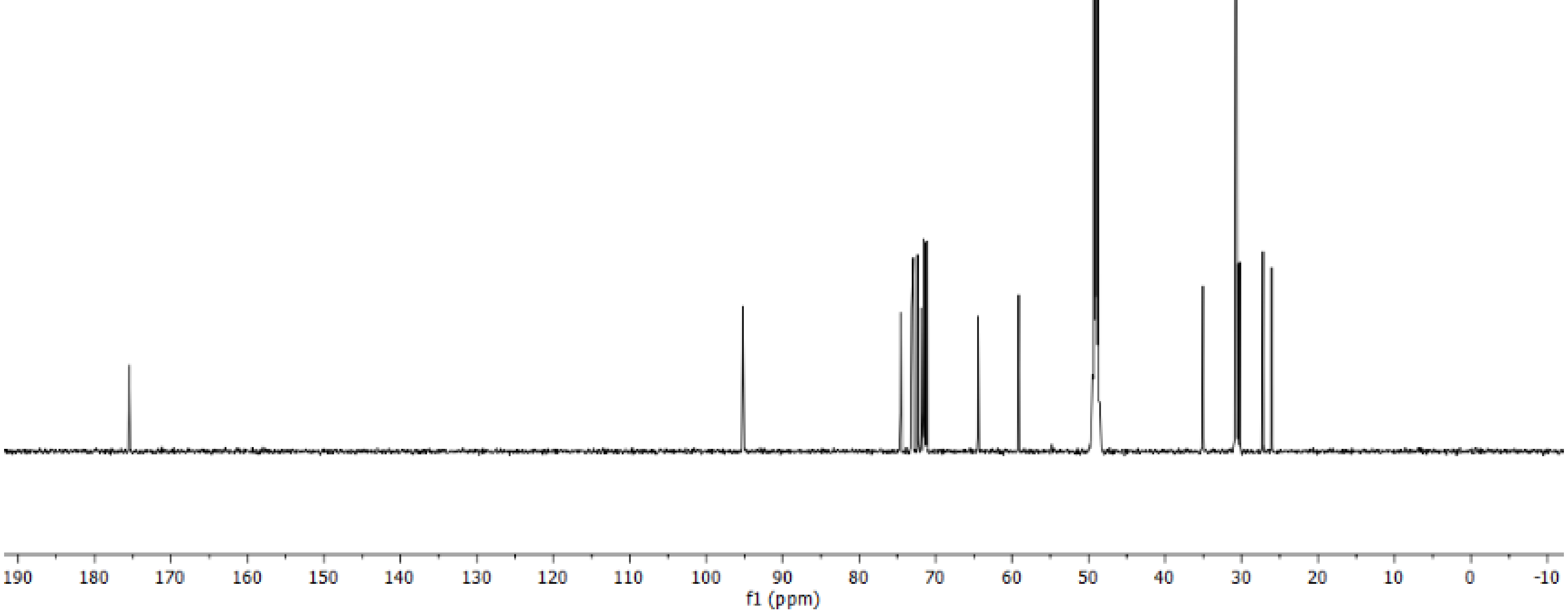

^{13}C NMR (150 MHz, CD_3OD)

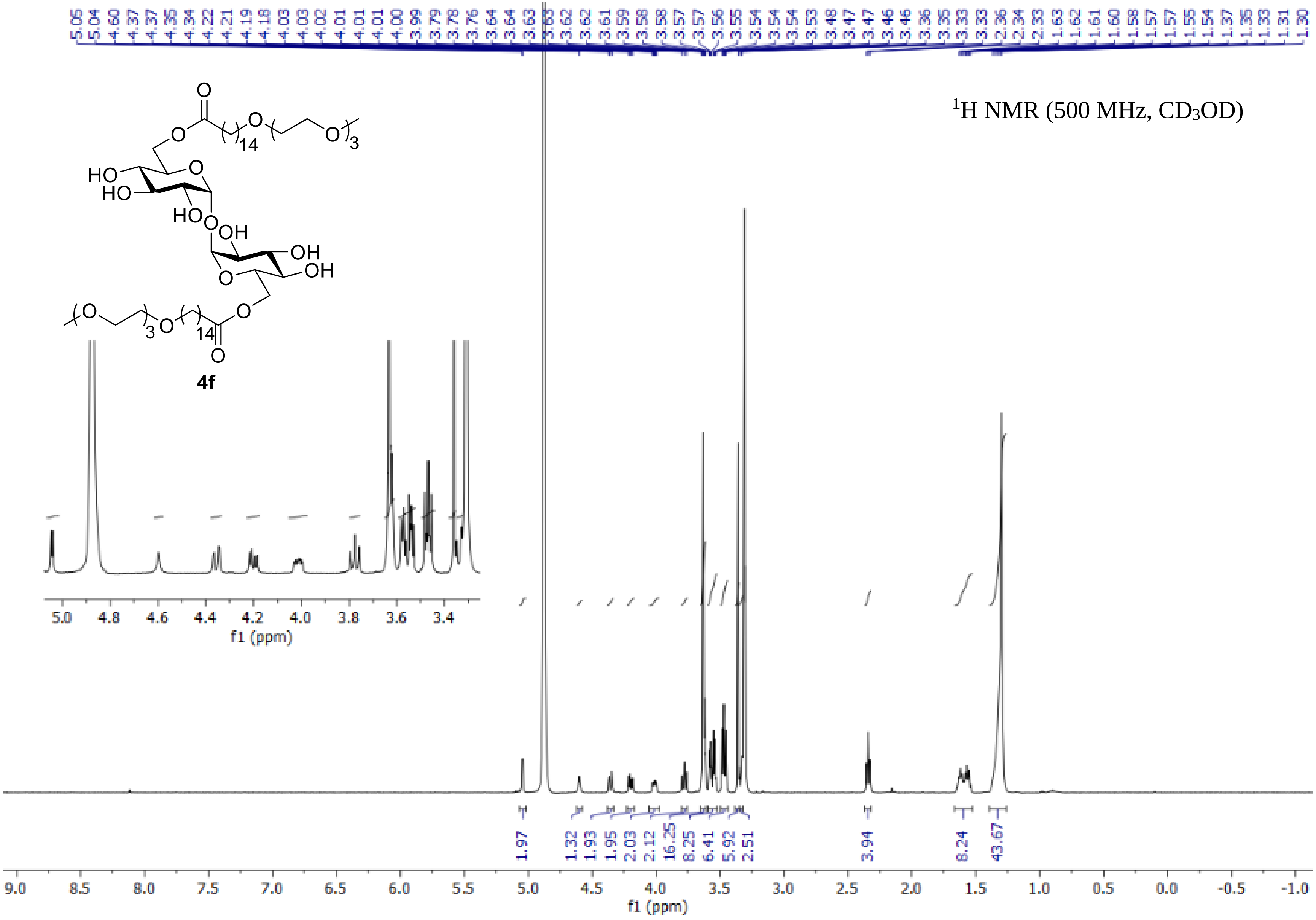

^{1}H NMR (500 MHz, CD3OD)
4f
f1 (ppm)

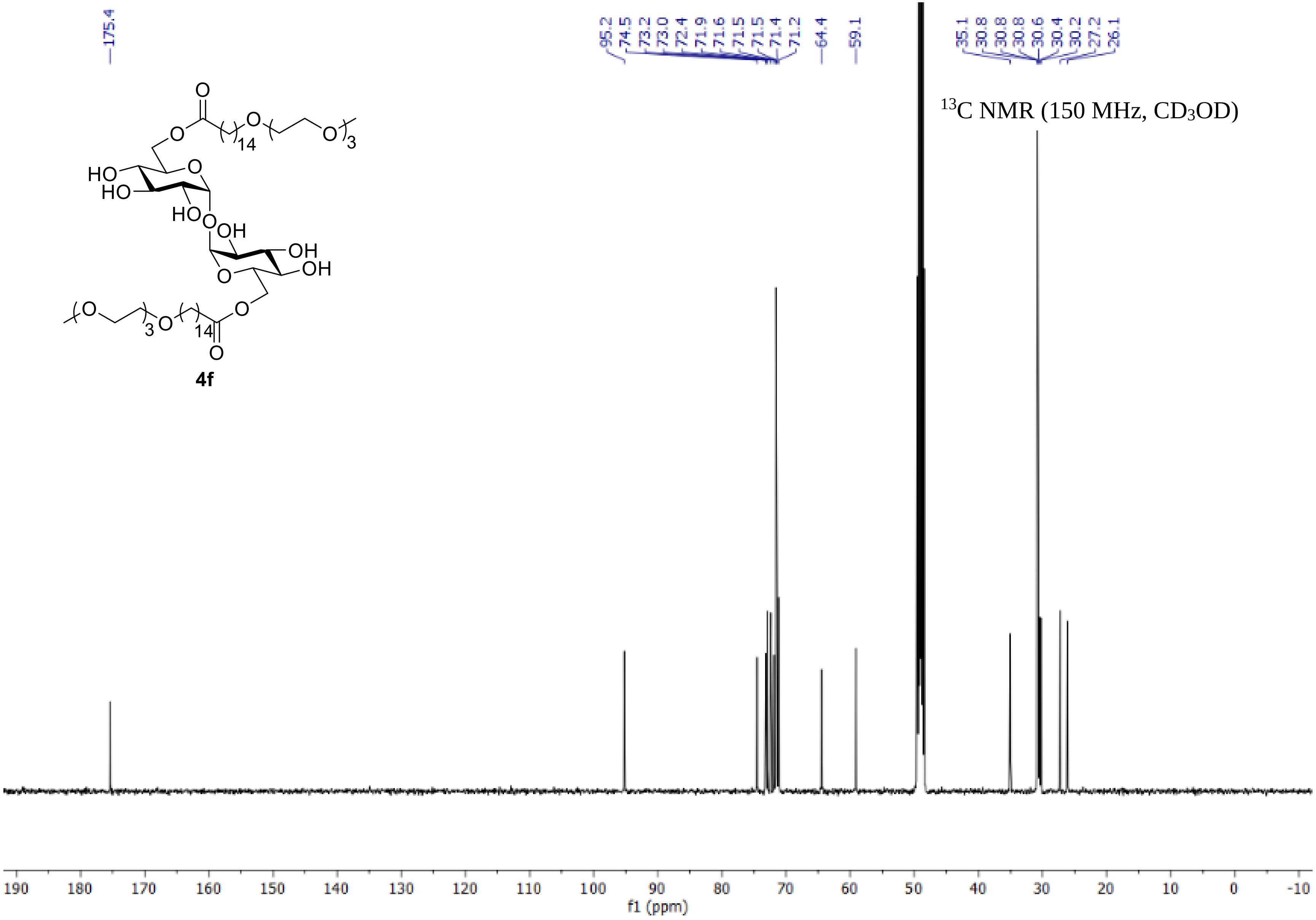
175.4
95.2
74.5
73.2
73.0
72.4
71.9
71.6
71.5
71.5
71.4
71.2
64.4
59.1
35.1
30.8
30.8
30.8
30.6
30.4
30.2
27.2
26.1
13C NMR (150 MHz, CD3OD)
4f
190 180 170 160 150 140 130 120 110 100 90 80 70 60 50 40 30 20 10 0 -10
f1 (ppm)

3 Supplementary Biological Data

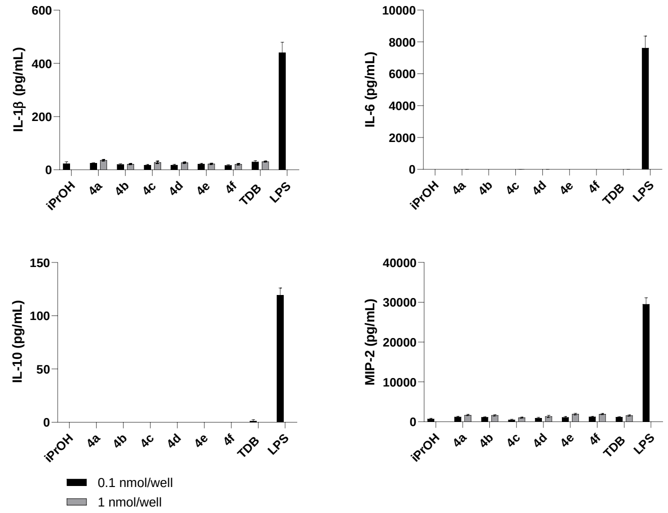

Supplementary Figure 1. Cytokine production by PEG-TGLs (**4a-f**) using Mincle$^{-/-}$ GM-CSF treated bone-marrow cells. Mincle$^{-/-}$ GM-CSF treated bone-marrow cells were stimulated with TDB or **4a-f** (0.1 or 1 nmol/well) coated on plates, or LPS (100 ng/mL), and IL-1β, IL-6, MIP-2, and IL-10 production were measured by ELISA from the supernatants collected after 24 h. Data represents the mean of three independent experiments performed in triplicate (mean ± SEM).

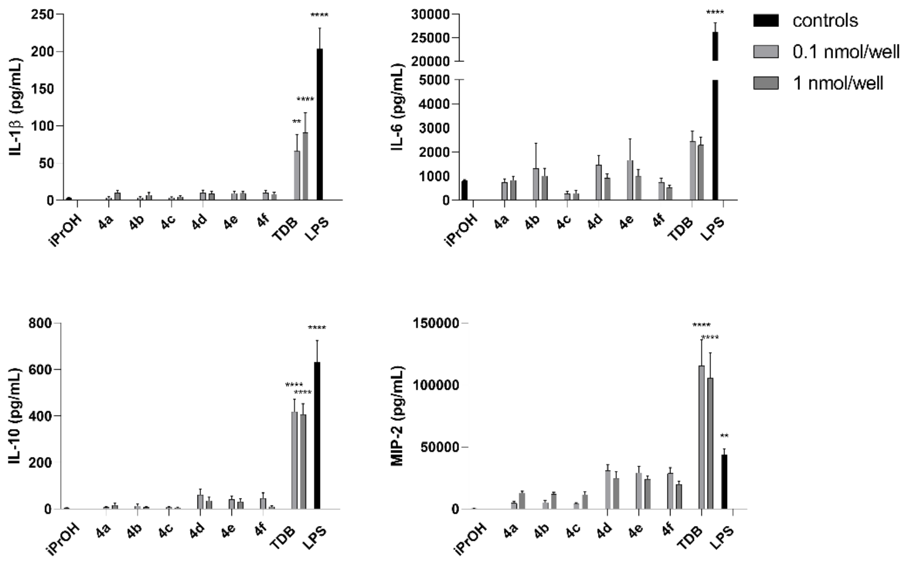

Supplementary Figure 2. WT C57 GM-CSF (20 ng/mL) treated BMCs were stimulated with TDB or **4a-f** (0.1 or 1 nmol/well) coated on plates, or LPS (100 ng/mL), and IL-1β, IL-6, MIP-2, and IL-10 was measured by ELISA from the supernatants collected after 24 h. Data represents the mean of three independent experiments performed in triplicate (mean ± SEM). Statistical significance was calculated in comparison to iPrOH control using two-way ANOVA (Dunnett's multiple comparison test), $*P \leq 0.05$; $**P \leq 0.01$; $***P \leq 0.001$; $****P \leq 0.0001$.

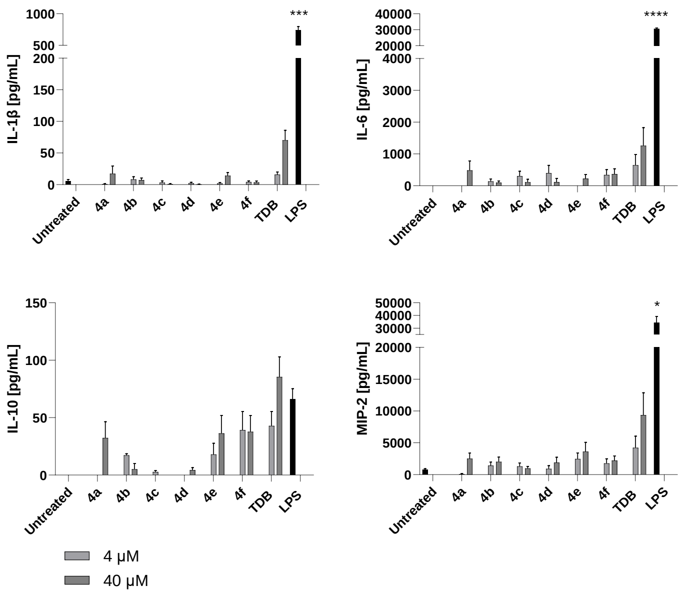

Supplementary Figure 3. Cytokine production for PEG-TGLs (**4a-f**) using non-plate coated assay. C57 GM-CSF treated bone-marrow cells were stimulated with TDB or **4a**-**f** (4 µM or 40 µM; solubilized in 2% DMSO in H_2O), or LPS (100 ng/mL). IL-1β, IL-6, IL-10 and MIP-2 production was measured by ELISA from the supernatants collected after 24 hours. Data are representative of two independent experiments performed in triplicate (mean ± SEM). Statistical significance was calculated in comparison to untreated control, using one-way ANOVA (Dunnett's multiple comparison test), **P*≤ 0.05; ***P*≤ 0.01; ****P*≤ 0.001; *****P*≤ 0.0001.

4 References

1. Davey, T. W.; Hayman, A. R., Synthesis of ω-Hydroxy Quaternary Ammonium Bolaform Surfactants. *Aus. J. Chem.* **1998**, *51* (7), 581-586

2. Johnson, D. A., Simple procedure for the preparation of trimethylsilyl ethers of carbohydrates and alcohols. *Carbohydrate Research* **1992**, *237*, 313-318

3. Khan, A. A.; Chee, S. H.; McLaughlin, R. J.; Harper, J. L.; Kamena, F.; Timmer, M. S. M.; Stocker, B. L., Long-Chain Lipids Are Required for the Innate Immune Recognition of Trehalose Diesters by Macrophages. *ChemBioChem* **2011**, *12* (17), 2572-2576
